# USF2-mediated upregulation of TXNRD1 contributes to hepatocellular carcinoma progression by activating Akt/mTOR signaling

**DOI:** 10.1038/s41419-022-05363-x

**Published:** 2022-11-01

**Authors:** Wen-ya Huang, Zhi-bin Liao, Jia-cheng Zhang, Xin Zhang, Hong-wei Zhang, Hui-fang Liang, Zun-yi Zhang, Tao Yang, Jia Yu, Ke-shuai Dong

**Affiliations:** 1 Department of Integrated Traditional Chinese and Western Medicine, Tongji Hospital, Tongji Medical College, Huazhong University of Science and Technology, Wuhan, China; 2 Hepatic Surgery Center, Tongji Hospital, Tongji Medical College, Huazhong University of Science and Technology, Wuhan, China; 3 Hubei Key Laboratory of Hepato-Pancreato-Biliary Disease, Wuhan, China; 4 Department of General Surgery, Renmin Hospital of Wuhan University, Wuhan, China; 5 Department of Hepatobiliary Surgery, Renmin Hospital of Wuhan University, Wuhan, China

**Keywords:** Liver cancer, Tumour-suppressor proteins

## Abstract

Thioredoxin reductase 1 (TXNRD1) is one of the major redox regulators in mammalian cells, which has been reported to be involved in tumorigenesis. However, its roles and regulatory mechanism underlying the progression of HCC remains poorly understood. In this study, we demonstrated that TXNRD1 was significantly upregulated in HCC tumor tissues and correlated with poor survival in HCC patients. Functional studies indicated TXNRD1 knockdown substantially suppressed HCC cell proliferation and metastasis both in vitro and in vivo, and its overexpression showed opposite effects. Mechanistically, TXNRD1 attenuated the interaction between Trx1 and PTEN which resulting in acceleration of PTEN degradation, thereby activated Akt/mTOR signaling and its target genes which conferred to elevated HCC cell mobility and metastasis. Moreover, USF2 was identified as a transcriptional suppressor of TXNRD1, which directly interacted with two E-box sites in TXNRD1 promoter. USF2 functioned as tumor suppressor through the downstream repression of TXNRD1. Further clinical data revealed negative co-expression correlations between USF2 and TXNRD1. In conclusion, our findings reveal that USF2-mediated upregulation of TXNRD1 contributes to hepatocellular carcinoma progression by activating Akt/mTOR signaling.

## Introduction

Liver cancer is the seventh most frequently occurring cancer and the second leading cause of cancer-related deaths in the world [1, 2]. The majority of liver cancer cases are potentially preventable because most liver cancer risk factors are modifiable (eg, hepatitis B and C viruses, excess alcohol consumption, obesity, and cigarette smoking). However, liver cancer is increasing most rapidly for all cancers, by 2% to 3% annually during 2007 through 2016 [3]. Hepatocellular carcinoma (HCC) is the dominant type of liver cancer, accounting for approximately 75% of the total liver cancer [4, 5]. Nowadays, despite great advances in therapeutic interventions, the prognosis of HCC remain dismal due to postoperative recurrence and metastasis after liver resection [6]. It is therefore urgent to a more comprehensive understanding of HCC progression at the molecular level for developing effective therapies.

Akt signaling is an evolutionarily conserver kinase cascade pathway, whereas dysregulation of Akt signaling contributes to cancer development. Typically, Akt is one of the major downstream effectors of PI3K, and its modification is sufficient to activate the mammalian target of rapamycin complex (mTOR) [7]. Disturbed activation of PI3K/Akt/mTOR pathway is associated with many human malignancies, thus represents important target for development of potential antitumor agents [8–11]. In normal conditions, the Akt signaling pathway is negatively regulated by phosphatase and tension homolog (PTEN), which limits the ability of Akt to bind to the membrane, decreasing its activity [12, 13]. A number of post-translational mechanisms regulate PTEN activity and stability (half-life) and these include oxidation, phosphorylation, acetylation, ubiquitination and SUMOylation [14]. However, the detailed mechanisms by which these factors regulate Akt signaling and affect HCC progression have not yet been fully understood.

The thioredoxin system, which comprises thioredoxin (Trx), thioredoxin reductase (TXNRD), thioredoxin-interacting protein (TXNIP) and NADPH, is indispensable for retaining harmony and regulation for the redox status in cells [15]. Our previous study had demonstrated that PX-12, an inhibitor of thioredoxin 1 (Trx1), had anti-tumor activity and a synergistic effect in combination with 5-fluorouracil in HCC [16]. Thioredoxin reductase 1 (TXNRD1) is one of the major redox regulators in mammalian cells and an essential selenium-containing protein in antioxidant defense, redox regulation, DNA repair, angiogenesis, proliferation and transcription by keeping thioredoxin 1 (Trx1) in the reduced state [15]. TXNRD1 inhibition can lead to an impairment of required antioxidant capacity particularly in cancer cells, while normal cells can survive a loss of TXNRD1 activity, it is thus not far-fetched to propose that TXNRD1 inhibition may be a potential mechanism of action for anticancer drugs [17]. Previous studies have revealed that overexpression of TXNRD1 modulates drug-specific cytotoxic responses [18] and inhibition of the TXNRD1 enhances the efficacy of some chemotherapeutics, such as improving ibrutinib’s anti-EGFR activity in lung cancer [19]. Moreover, high expression level of TXNRD1 has been reported in various cancers, such as breast, prostate and thyroid cancers, and is associated with aggressive tumor and poor prognosis [20–23]. Recent studies demonstrated that TXNRD1 was upregulated in HCC cells and tissues and was an unfavorable prognostic factor for HCC [24, 25]. Driven by the transcriptional activation of NRF2, TXNRD1 counteracts intracellular ROS produced in HCC and enhances the proliferation of HCC cells [23]. It is less clear, however, by which, if any, the mechanisms of TXNRD1 that are required for HCC development, conversely, by which mechanism that is responsible for the regulation of TXNRD1 overexpression in HCC.

This study focuses on the role of TXNRD1 in HCC progression, which also sheds light on the regulatory mechanism of TXNRD1 in HCC. Here, we identified TXNRD1 as a tumor promoter that stimulates HCC proliferation and metastasis through activating AKT/mTOR signaling, and Upstream transcription factor 2 (USF2) was a major transcriptional repressor of TXNRD1 expression.

## Material and methods

### Clinical HCC samples

A total of 112 paired HCC tumor tissues and adjacent normal tissues were collected and subjected to Western blotting analysis. Tissue microarray plates containing another 115 HCC cases were constructed from paraffin-embedded HCC tissues for IHC and clinical analysis. Tissue samples were obtained from resected specimens at Tongji Hospital, Wuhan, China. The diagnosis of HCC was based on the pathological examination. Informed consent for data analysis was obtained from each patient, and the study protocol was authorized by the Medical Ethics Committee of Tongji Hospital and complied with all relevant ethical regulation.

### Mouse tumor model

Male BALB/c nude mice (5 weeks old) were purchased from HUAFUKANG (HUAFUKANG BIOSCIENCE CO. INC. Beijing, China) and fed under the specific pathogen-free condition at optimal temperature and humidity. All animal experiments were conducted in accordance with the National Institutes of Health Guidelines for the Care and Use of Laboratory Animals and approved by the Ethics Committee of Tongji Hospital of Tongji Medical College.

For subcutaneous tumorigenicity assay, 1 × 10^6^ cells were subcutaneously injected into the dorsal part of mice. Tumor size were recorded every three days from day 7 to 9. When tumors grew to 3–5 mm in diameter, the mice were peritoneally treated with Auranofin (10 mg/kg, two times per week). The mice were euthanized 5 weeks after injection, and tumors were excised and weighed. Mice bearing a tumor with a tumor size large than 15 mm in any direction were euthanized. Tumor volume was calculated by length × width ^2^ /2. The excised tumors were embedded in paraffin for IHC analysis.

For the liver in-situ xenograft model, 5 × 10^6^ cells were injected subcutaneously into the flank of nude mice. When the subcutaneous tumor reached approximately 1 cm in length, it was removed, minced into small pieces of equal volume (1 mm^3^), and transplanted into the left liver lobe of nude mice. Mice were euthanized and the liver tissue specimens were collected after 6 weeks. The incidence of liver tumors was recorded.

For lung metastasis assay, 1 × 10^6^ cells were injected into tail vein of nude mice. The mice were sacrificed at 8 weeks after injection, their lungs were removed and fixed with paraformaldehyde (4%) and embedding in paraffin. Paraffin sections were stained with H&E according to standard protocol and metastatic nodules were calculated with a microscopy to evaluate the development of lung metastasis.

### Luciferase reporter assay

The TXNRD1 promoter regions 2000bp from the transcription start site (−2000bp-+1 bp) were cloned into pGL4.17 firefly luciferase reporter vector. The promoter truncations and E-box mutant was constructed by site-directed mutagenesis. Cells were seeded at a density of 10^5^ cells per well in 24-well plates and allowed to settle for 24 h. Each well was transiently transfected TXNRD1 firefly luciferase reporter construct and the pRL-TK renilla luciferase vector for normalization of transfection efficiency together with pcDNA3.1 vector or pcDNA3.1-USF2 using Lipofectamine 2000 transfection reagent in triplicate. After 8 h, the transfection media was replaced with fresh complete DMEM.

The cocultures were lysed 48 h post-transfection and were assayed sequentially for Firefly and Renilla luciferase using the Dual-Luciferase Reporter Assay System (E1910, Promega) with a Glo/Max 20/20 Luminometer (Promega) according to the manufacturer’s instructions. Relative light units were calculated as the ratio of Firefly luciferase activity to Renilla luciferase activity as described previously [26].

### Chromatin immunoprecipitation

Both Huh7 and Bel-7402 cells were subjected to chromatin immunoprecipitation analysis using the SimpleChIP Plus Sonication Chromatin IP Kit (Cell Signaling Technology) following the manufacturer’s instructions. Briefly, 1 × 10^7^ cells were harvested and crosslinked with 1% formaldehyde for 10 min at room temperature. After glycine quenching, samples were lysed with sodium dodecyl sulfate buffer and sonicated to shear DNA. Fragmented chromatin extracts with an average of 250-1000 bp were incubated with anti-USF2 antibody and normal mouse IgG at 4 °C overnight with rotation. Immunoprecipitated products were captured by incubating with Protein G Magnetic Beads followed by washes. Elutes were subjected to reverse cross-linking and then digested with RNase A and proteinase K. Immunoprecipitated DNA and input were purified for qPCR analysis with primers specifically targeting the TXNRD1 promoter region that encompassed the USF2-binding E-box site. Primer sequences were listed in Supplementary Table S1. PCR products were separated on agarose gels and visualized by ethidium bromide staining. The value of enrichment was calculated relative to the input and ratio to IgG.

### Statistical analysis

Statistical analyses were performed using SPSS software (version 21.0, IBM Corp, Armonk, NY, USA) or the GraphPad Prism software (version 6.01, GraphPad Software Inc., San Diego, CA). Values were expressed as the mean ± SD or mean ± SEM from at least three independent experiments. Quantitative variables were compared using Student’s t-test or Mann-Whitney-U test when applicable. Also, One-way ANOVA followed by a Turkey post hoc test was performed for multigroup comparison. Categorical variables were compared with Pearson’s χ^2^ or Fisher’s exact test. Correlations were determined by Pearson’s correlation. Cumulative survival curves were calculated according to the Kaplan-Meier method and compared by the log-rank test. Statistical significance was determined as indicated in the figure legends, set to *P* < 0.05 and represented as *P < 0.05, **P < 0.01, ***P < 0.001.

Additional materials and methods can be found in Supplementary Data.

## Results

### TXNRD1 is highly expressed in HCC and associated with poor prognosis in HCC patients

**Fig. 1:**
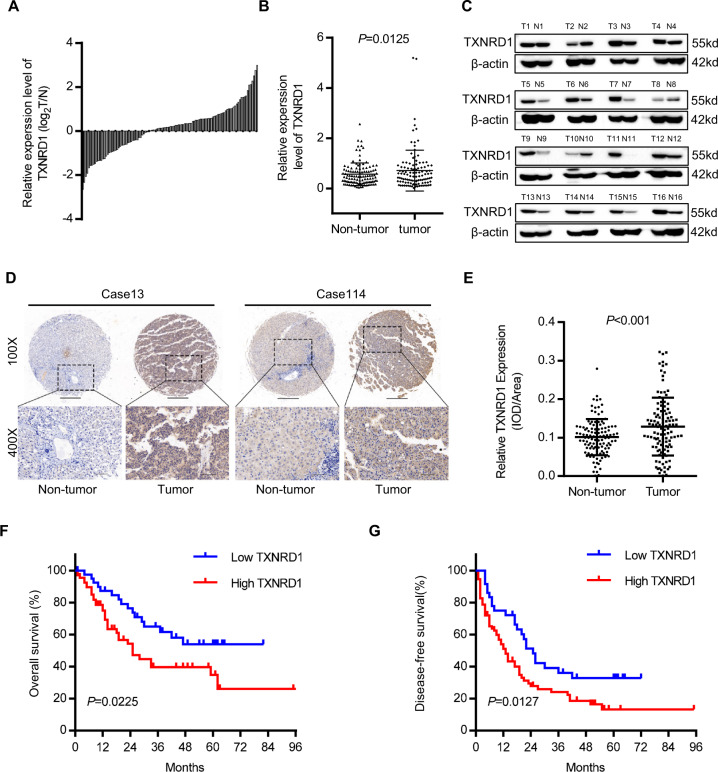
TXNRD1 is enriched in HCC and associated with poor prognosis of HCC patients. **A** Protein levels of TXNRD1 were evaluated by western blotting in 112 paired HCC tumor and adjacent non-tumor tissues. TXNRD1 level of tumor were quantified in the bar chart after normalized to their adjacent non-tumor tissues. **B** Relative grey levels of TXNRD1 in tumor and non-tumor tissues were quantified. *P* value was calculated by Paired t test. **C** Representative western blotting images of TXNRD1 expression in 16 paired tissues are shown. T tumor, N non-tumor. **D** Immunohistochemical staining (IHC) for TXNRD1 was performed in 115 paired tissues from HCC patients. Representative IHC images are shown. **E** Dot density plot shows the quantification of immunohistochemical TXNRD1 staining. *P* value was calculated by Paired t test. **F**–**G** Kaplan–Meier analysis for overall survival (**F**) and disease-free survival (**G**) was performed according to high or low expression of TXNRD1 in HCC tumor. *P* value was calculated by log-rank test.

To verify the clinical relevance of TXNRD1 in HCC, data mining from the ONCOMINE database of three HCC cohorts indicated that TXNRD1 mRNA was strongly upregulated in HCC tumor tissues compared with adjacent normal tissue samples (Fig. S1A). Then, we evaluated TXNRD1 protein level in 112 paired HCC tumor and adjacent non-tumor tissues by western blot. Overexpression of TXNRD1 was detected in 62.5% (70/112) HCC tissues as compared with the adjacent non-tumor tissues (Fig. 1A). The protein expression level of TXNRD1 was significantly higher in HCC tumor tissues compared with their adjacent normal tissues (Fig. 1B). Representative western blotting images of TXNRD1 expression in 16 paired tissues are shown in Fig. 1C. In addition, immunohistochemical staining (IHC) was carried out to examine the expression of TXNRD1 in another cohort of 115 paired HCC tissues in tissue microarray (Fig. 1D). Consistently, TXNRD1 expression was significantly higher in tumor tissues compared with their adjacent normal tissues (Fig. 1E). According to IHC analysis, 115 patients were divided into two groups: low TXNRD1 group (*n* = 37) and high TXNRD1 group (*n* = 78). The detailed clinicopathological features is summarized in Table S2. Chi-squared analysis indicated that there was no correlation between TXNRD1 expression status and clinicopathological features such as age, gender, HBsAg, ALT, AST, AFP, Child score, tumor size, tumor number, vascular invasion, tumor differentiation, TNM stage. However, high TXNRD1 expression was positively associated with liver cirrhosis (*P* = 0.028). Regarding the correlation of TXNRD1 expression with postoperative outcomes, Kaplan-Meier’s analysis revealed that patients with high TXNRD1 expression demonstrated significantly shorter overall survival (OS) and disease-free survival (DFS) than those with low expression (Fig. 1F, G). Additionally, we found that TXNRD1 expression level was elevated and highly correlated prognosis of HCC in TCGA database (Fig. S1B, C). Therefore, these clinical results suggest that TXNRD1 expression may associate with HCC development and represent an important prognostic indicator for HCC in the clinical setting.

### TXNRD1 promotes HCC proliferation and contributes to HCC metastasis in vitro

**Fig. 2:**
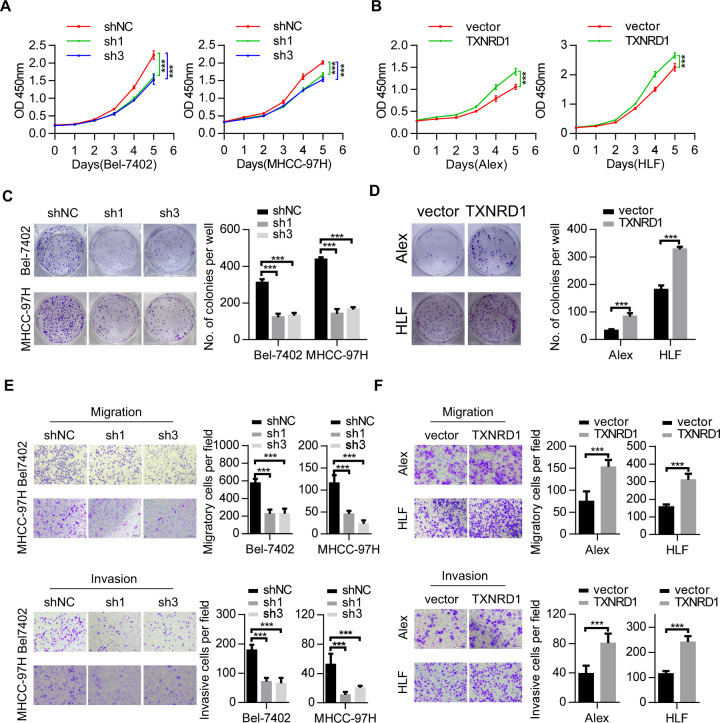
TXNRD1 promotes proliferation and metastasis of HCC cells in vitro. **A** Proliferation abilities of HCC cells with TXNRD1 knockdown compared to the control vector were investigated by cell growth curve and CCK-8 assay. **B** Proliferation abilities of HCC cells with TXNRD1 overexpression compared to the control vector were investigated by cell growth curve and CCK-8 assay. **C** The anchorage-depend colony formation assay was performed to assess the effects of TXNRD1 knockdown on clone formation ability of HCC cells. **D** The anchorage-depend colony formation assay was performed to assess the effects of TXNRD1 overexpression on clone formation ability of HCC cells. **E** Cells migration and invasion abilities in HCC cells with TXNRD1 knockdown were determined by transwell assay. Representative image (left) and summary bar chart (right) are shown. Scale bar, 200 μm. **F** Cells migration and invasion abilities in HCC cells with TXNRD1 overexpression were determined by transwell assay. Representative image (left) and summary bar chart (right) are shown. Scale bar, 200 μm. Data represent the mean ± SEM. ***P < 0.001. *P* values were calculated by One-way ANOVA (**A**, **C**, **E**) or Student’s t test (**B**, **D**, **F**).

The above clinical results suggested that TXNRD1 may play a functional role in HCC progression. Accordingly, we evaluated the functions of TXNRD1 in HCC cells. To evaluate the effects of TXNRD1 on cell proliferation, colony formation, migration and invasion, we first detected the endogenous TXNRD1 levels in different liver and HCC cell lines by western blot (Fig. S2A). Then, we chose Bel-7402, MHCC-97H cell as TXNRD1 high expression cell lines and selected Alex, HLF cells as SHC4 low expression cell lines for further experiments. TXNRD1 levels were modulated using lentivirus-mediated TXNRD1-specific short hairpin RNAs(shRNA) or lentivirus vector TXNRD1 (Fig. S2B). sh1-TXNRD1 and sh3-TXNRD1, which induced the more significant knockdown effect, was adopted for further study. The variation of enzymatic activity of TXNRD1 was also confirmed in knockdown and overexpressed cells (Fig. S2C). Cell proliferation assays demonstrated that knockdown of TXNRD1 in Bel-7402 and MHCC-97H cells significantly inhibited cell viability compared with their vector controls (Fig. 2A), while an inverse effect was observed in Alex and HLF cells with TXNRD1 overexpression (Fig. 2B). In keep with this, knockdown of TXNRD1 decreased the number and size of colonies formed in Bel-7402 and MHCC-97H cells (Fig. 2C), whereas ectopic expression of TXNRD1 enhanced the colony formation ability in Alex and HLF cells (Fig. 2D). Further, we examined the effect of TXNRD1 on cell migration and invasion by transwell assay. The results showed that TXNRD1 knockdown significantly decreased cell migrative and invasive abilities in Bel-7402 and MHCC-97H cells compared with their controls (Fig. 2E). Moreover, Alex-TXNRD1, HLF-TXNRD1 cells exhibited a significant enhance in cell migration and invasion compared with their empty vector cells (Fig. 2F). Together, these data suggest that TXNRD1 plays a critical role in promoting proliferation and metastasis of HCC cells.

### TXNRD1 promotes HCC growth and metastasis in vivo

**Fig. 3:**
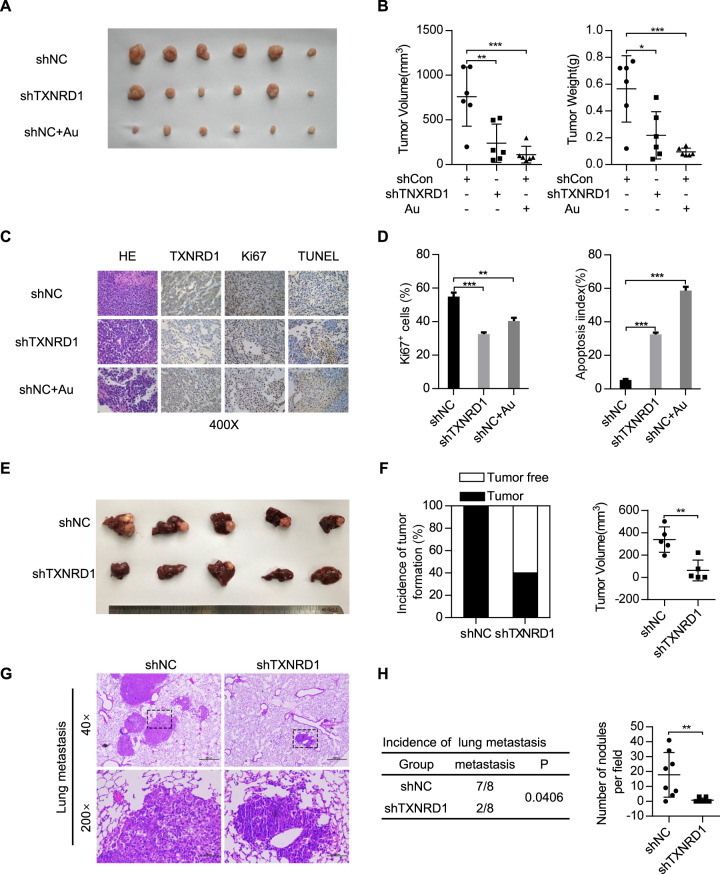
TXNRD1 knockdown inhibits tumor growth and metastasis of HCC cells in vivo. **A** Bel-7402 cells with TXNRD1 knockdown or control were injected into the flank of nude mice. After the tumors grew to 3-5 mm in diameter, Auranofin (Au) was treated with 10 mg/kg twice per week. Images of tumors excised from six nude mice at 5 weeks after inoculation were taken. **B** Dot plots show the volume and weight of indicated tumors. **C** IHC images of TXNRD1, Ki67 and TUNEL in xenograft tumors were presented. **D** Quantitative IHC analysis of Ki-67 and apoptosis in xenograft tumors from each group in (**C**). **E** Photos of liver in-situ tumor formation and mice liver were taken after 6 weeks of orthotopic transplantation. **F** Statistical analysis of the incidence of intrahepatic tumor is shown (left panel). Intrahepatic tumor volumes of different groups are summarized in dot chat (right panel). **G** Bel-7402 cells with TXNRD1 knockdown or control were injected into tail vein of nude mice. Representative HE staining images of lung metastases were presented. **H** Statistical analysis of the incidence of lung metastases is shown (left panel). The number of visible metastatic nodules of different groups are counted in dot chat (right panel). Data represent the mean ± SEM. *P < 0.05, **P < 0.01, ***P < 0.001. *P* values were calculated by Student’s t test (**B**, **D**, **F**, **H**) or Pearson’s chi-square test (**H**).

To validate these findings, we further examined the effects of TXNRD1 expression on in vivo tumor growth and metastasis of HCC cells in vivo. In subcutaneous implantation nude mice model, TXNRD1-silenced cells or TXNRD1-overexpressing cells were injected into the flanks of nude mice. At the end of the experiment, the mice were sacrificed and xenografts were excised (Fig. 3A). The nude mice injected with shTXNRD1-transfected Bel-7402 cells were found have much smaller tumor volume and tumor weight than those injected with shRNA control transfected cells (Fig. 3B). Whereas TXNRD1 overexpressing HLF cells showed a significant increase in their ability to form tumors in nude mice compare to vector-transfected cells (Fig. S3A, B). The expression levels of TXNRD1 and Ki67 were decreased in tumors formed by TXNRD1-silenced cells (Fig. 3C, D). More apoptotic cells were observed in TXNRD1 knockdown xenografts as indicated by terminal deoxynucleotidyl transferase-mediated deoxyuridine triphosphate nick-end labeling (TUNEL) assay (Fig. 3C, D). To assess the potential of TXNRD1-targeted therapy, we evaluated the antitumor activity of the TXNRD1 inhibitor Auranofin (Au) in vivo. Treatment with Auranofin resulted in significant suppression of xenograft tumor growth (Fig. 3A, B). Similar with TXNRD1 knockdown, fewer proliferating cells and more apoptotic cells were detected in xenograft tumors with Auranofin treatment as indicated by Ki-67 staining and TUNEL assays (Fig. 3C, D). Liver in-situ xenograft model were established using Bel-7402 cells with or without TXNRD1 knockdown. The results showed that knockdown of TXNRD1 in Bel-7402 cells significantly decreased the incidence of orthotopic liver tumor and the tumor volume compared to controls (Fig. 3E, F). The lung metastasis model by tail vein injection was used to investigate the function role of TXNRD1 in metastasis. Mice were sacrificed after 8 weeks and lungs were removed (Figure S3C), and consecutive sections were taken from every lung tissue block and stained with H&E (Fig. 3G). In contrast, mice injected with TXNRD1-knockdown cells experienced less of detectable metastases. Histologic results supported the observations and disclosed a higher incidence of lung metastasis and more metastatic lesions produced by control cells whereas TXNRD1-knockdown cells lacked metastatic lesions. Consistent with the function of TXNRD1 in vitro, TXNRD1 contributes to cell proliferation and metastasis in HCC.

### TXNRD1 facilitates HCC proliferation and metastasis through the Akt/mTOR signaling pathway

**Fig. 4:**
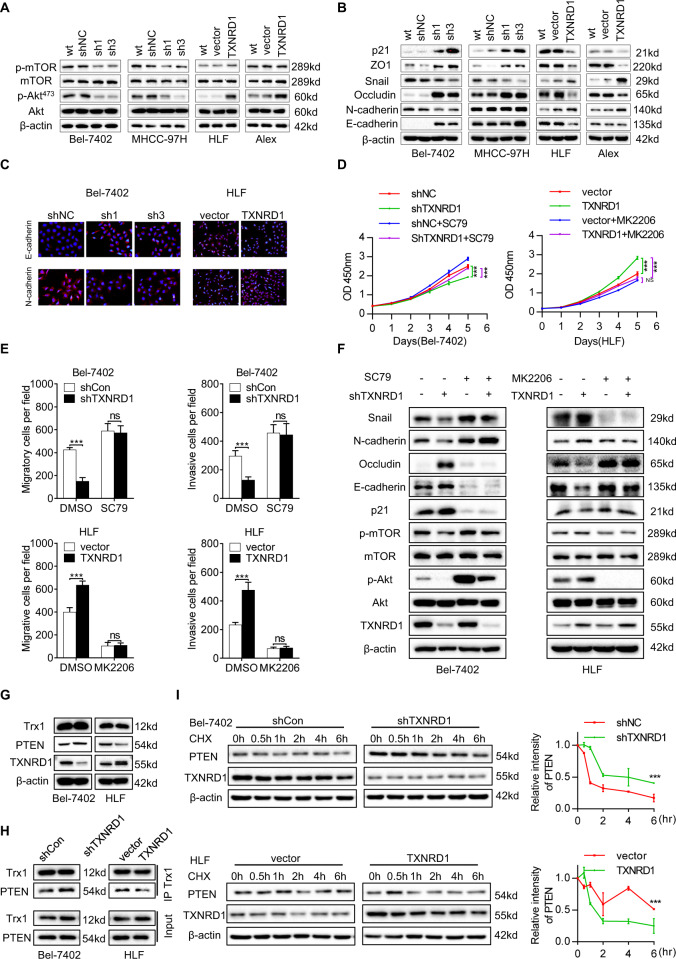
TXNRD1 activates the AKT-mTOR signaling pathway to enhance the proliferation and metastasis of HCC cells. **A** TXRND1 was knockdown by two different shRNA in Bel-7402 and MHCC-97H cells. TXNRD1 was overexpressed in HLF and Alex cells. The expression of Akt, p-Akt, mTOR, and p-mTOR was detected by western blot. **B** Western blot was performed to detected the level of E-cadherin, N-cadherin, Occludin, Snail, ZO1,p21 in the indicated cells with TXRND1 knockdown or overexpression. **C** Immunofluorescence images of EMT markers in Bel-7402 and HLF cell described in **A** and **B**. **D** Proliferation abilities of HCC cells treated with SC79 (10 μM) or MK2206 (5 μM) were investigated by cell growth curve and CCK-8 assay. **E** Cells migration and invasion abilities in indicated cells treated with SC79 (10 μM) or MK2206 (5 μM) were determined by transwell assay. **F** After treatment with SC79 (10 μM) or MK2206 (5 μM) for 24 hours, protein levels of indicated makers were analyzed by western blot analysis in the indicated cell lysates. **G** Western blot assays for TXNRD1, PTEN and Trx1 expression in the Bel-7402 and HLF cells. **H** Immunoprecipitation analysis showed that in Bel-7402 cells, silencing of TXNRD1 enhanced Trx1-PTEN interaction, whereas in HLF cells, TXNRD1 overexpression attenuated Trx1-PTEN interaction. **I** To prevent de novo PTEN biosynthesis, 5 μg/ml cycloheximide (CHX) was used for indicated time intervals. Expression of PTEN and TXNRD1 was determined by western blot in CHX experiment (left). Protein half-life of PTEN was quantitatively defined (right). Data represent the mean ± SEM. ns, not significant; **P < 0.01, ***P < 0.001. *P* values were calculated by Student’s t test.

Given that TXNRD1 in mainly responsible for redox homoeostasis [27, 28], which is unlikely to be a mechanism directly mediating HCC proliferation and metastasis, we speculated that TXNRD1 might function through downstream signaling molecules. It was reported that the synthetic lethality interaction between the TXNRD1 and Akt pathways occurred through the KEAP1/NRF2 cellular antioxidant pathway [29]. As activation of Akt is closely correlated with oncogenesis and metastasis of different cancers, TXNRD1 might promoted HCC development and metastasis through regulation the Akt signaling pathway. This hypothesis was supported by the observation that Akt and mTOR activation significantly decreased when TXNRD1 was downregulated by shTXNRD1, which could be enhanced by overexpression of TXNRD1 (Fig. 4A). In addition, we also investigated the effects of TXNRD1 on the key downstream effectors of the Akt signaling for cancer development. These results demonstrated that TXNRD1 knockdown decreased N-cadherin, Snail expression but increased p21, ZO1, Occludin, E-cadherin expression (Fig. 4B). Similarly, IHC staining showed that the increased expression level of E-cadherin and decreased expression level of N-cadherin were observed in TXNRD1 knockdown cells, whereas inverse expression trend in TXNRD1 overexpression cells (Fig. 4C). To further confirm that the oncogenic role of TXNRD1 was induced by activation of Akt/mTOR signaling, the Akt activator SC79 and inhibitor MK2206 were used to investigate the correlation between Akt and cell proliferation and metastasis induced by TXNRD1. We found that suppression in cell proliferation, migration and invasion induced by TXNRD1 knockdown could be rescued by SC79 treatment, while MK2206 treatment abolished the growth and motility advantages of TXNRD1 overexpression in HCC cells (Fig. 4D, E, Fig. S4A–D). Furthermore, the results of western blot analysis showed that SC79 effectively rescued the expression levels of phosphorylated Akt, phosphorylated mTOR, Snail, and N-cadherin suppressed by TXNRD1 knockdown, but blunted the enhancement of E-cadherin, Occludin and p21 induced by TXNRD1 knockdown (Fig. 4F). An opposite expression trend of these proteins was observed in TXNRD1 overexpression cells when treated with MK2206 (Fig. 4F and Fig. S4E), indicating that the promoting effect of TXNRD1 in HCC proliferation and metastasis was via Akt/mTOR signaling pathway.

Our current results indicated that TXNRD1 enhanced the Akt phosphorylation and activated the Akt signaling pathway. However, the mechanism by which TXNRD1 activated the Akt signaling pathway in HCC remains unclear. It has been reported that Trx1 binds in a redox-dependent manner to PTEN to inhibit its phosphatidylinositol-3-phosphatase activity which results in increased Akt activation in cells [30]. Moreover, TXNRD1 is necessary for Trx1 to be maintained in a reduced state in the cell and is a prerequisite for the interaction between Trx1 and PTEN [30]. Therefore, we speculated that TXNRD1 might regulate PTEN and consequently activate the Akt signaling. To confirm this, western blot and qPCR were performed to determine the effect TXNRD1 on PTEN expression in protein and mRNA level. As expected, PTEN protein levels were clearly strengthened by TXNRD1 silencing and reduced by TXNRD1 overexpression, whereas no noticeable change was found in PTEN mRNA expression compared with control cells (Fig. 4G and Fig. S4F). Immunoprecipitation revealed that TXNRD1 clearly attenuated the binding of PTEN to Trx1 (Fig. 4H). Cyclohexamide (CHX) chase experiments were conducted to evaluated whether TXNRD1 altered PTEN protein expression by promoting protein degradation. When cells were treated with CHX, the PTEN protein decayed more rapidly in the presence of TXNRD1 transduction (Fig. 4I). Collectively, these data indicated that redox modification of TXNRD1 disturbed the interaction between Trx1 and PTEN, resulting in augmenting the degradation of PTEN and consequently activating the Akt/mTOR signaling.

### Transcription factor USF2 negatively regulates TXNRD1 expression in HCC

**Fig. 5:**
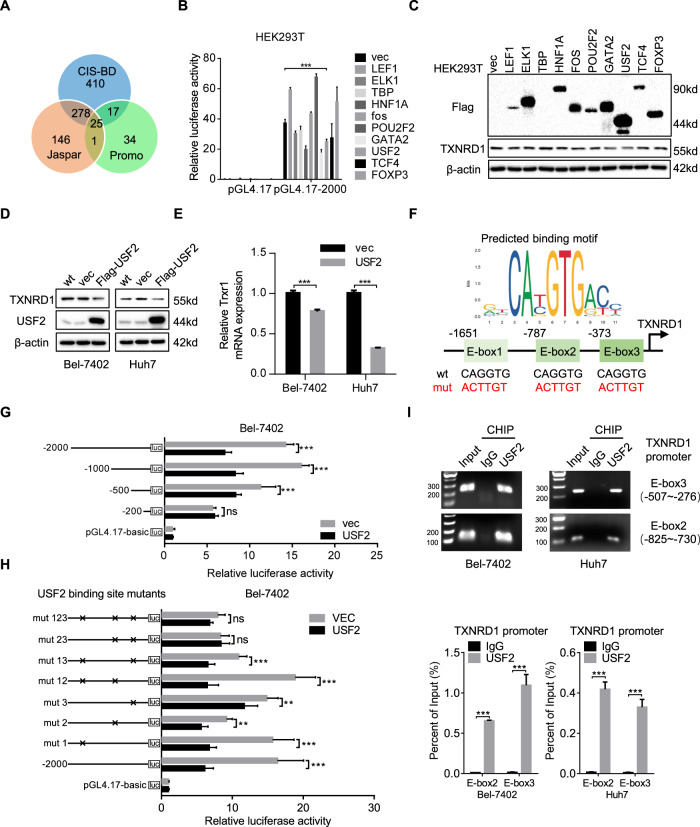
TXNRD1 is regulated by USF2 through the transcriptional suppression. **A** Venn diagrams show overlaps of putative transcription factors which might bind to the TXNRD1 promoter from three different database. **B** Relative luciferase activity was detected to analyze the regulation of ten transcription factor in the 2-kilobase human TXNRD1 promoter in HEK293T cells. **C** HEK293T cells were transiently transfected with ten transcription factors with Flag tag or empty vector for 24 h. Western blot was carried out to confirm the overexpression of indicated transcription factors and detect the protein level of TXNRD1. **D** Western blot analysis of TXNRD1 and USF2 following transient transfection of Flag-USF2 or vector control in Bel-7402 and Huh7 cells. **E** The mRNA levels of TXNRD1 were detected by qRT-PCR in Bel-7402 and Huh7 cells transiently transfected with Flag-USF2 or vector control. **F** Sequences of the canonical USF2-binding E-box motif, putative USF2-binding E-box sites in TXNRD1 promoter, and introduced point mutations used to inactivate the E-box site. **G** Fragments of the TXNRD1 promoter are shown. Relative luciferase activities of pGL4.17 containing TXNRD1 promoter truncations in Bel-7402 cells exposed to USF2 or control vector were measured by dual-luciferase assays. **H** Schematic E-box mutations of the TXNRD1 promoter are shown. Relative luciferase activities of pGL4.17 containing E-box site mutations in Bel-7402 cells exposed to USF2 or control vector were measured by dual-luciferase assays. **I** ChIP analysis of indicated cells immunoprecipitated by anti-USF2 antibody or IgG followed by qRT-PCR using specific primers targeting the USF2-bingding E-box sites in TXNRD1 promoter. Enrichment represent the percentage of input. Data represent the mean ± SEM. ns, not significant; **P < 0.01, ***P < 0.001. *P* values were calculated by Student’s t test.

To further explore the upstream mechanism of TXNRD1 overexpression in HCC, we focused our minds on transcriptional regulation of the 2 kb human TXNRD1 promoter region. We examined the 2 kb TXNRD1 promoter sequence with the CIS-BD, Jaspar and Promo search tool to screen putative transcription factor. Twenty-five transcription factors were identified by these three databases (Fig. 5A). Then, the 10 top scored transcription factors were selected as candidates. Combined with luciferase activity (Fig. 5B) and protein level (Fig. 5C) validation, this approach allowed us to identify USF2, Class B basic Helix-Loop-Helix transcription factor [31], as a negative transcription factor for TXNRD1 gene regulation. We showed that genetic overexpression of USF2 significantly reduced TXNRD1 expression at the protein and mRNA levels in Bel-7402 and Huh7 cells (Fig. 5D, E). Western blot analysis further revealed significant co-expression correlations between USF2 and TXNRD1 in the wild type cell lines, as strong evidence of USF2-mediated regulation of TXNRD1 (Figure S5A and S5B). Promoter analysis by CIS-BD, Jaspar and Promo revealed that TXNRD1 contained three conserved E-box elements within its 5’-promoter region (Fig. 5F). To map the USF2-binding E-box element in TXNRD1 promoter, we generated several luciferase reporters plasmids of various TXNRD1 promoter truncated mutants. Each was co-transfected with pcDNA3.1-USF2 or pcDNA3.1-vector into Bel-7402 cells. The luciferase activities of the 2 kb and 1 kb TXNRD1 promoter were decreased approximately 50% in the presence of USF2, while the decreased trend was weakened in 0.5 kb promoter. But no significant decrease was observed with the 0.2 kb promoter, demonstrating that E-box2 and E-box3 elements might be critical for USF2-mediated transcriptional repression of TXNRD1 (Fig. 5G). In addition, luciferase reporters of various E-box site mutants within the 2 kb promoter of TXNRD1 were constructed and co-transfected with pcDNA3.1-USF2 into Bel-7402 cells. Either individual E-box site mutants (mut 1, mut 2, mut 3) or double E-box site mutants (mut 12, mut 13) failed to block the inhibition of the TXNRD1 reporter by USF2. Double E-box site mutant (mut 23) and triple E-box site mutant (mut 123) disrupted USF2 binding to the TXNRD1 promoter as no repression was observed (Fig. 5H). Similar results were validated in Huh7 cells (Figure S5C and S5D). Chromatin immunoprecipitation (ChIP) assays were also conducted to confirm direct occupancy of USF2 with the E-box inTXNRD1 promoter. As expected, USF2 was enriched in the chromatin fragments containing the E-box2 and E-box3 DNA sequence in the promoter of TXNRD1 in Bel-7402 and Huh7 cells (Fig. 5I). These results taken together demonstrate that USF2 is a transcriptional repressor that negatively regulates the levels of endogenous TXNRD1, indicating that the upregulation of TXNRD1 in HCC could be due to the absence of USF2 repression.

### USF2 exerts an oncogenic suppression function in HCC, and the effect is reversed by TXNRD1 overexpression

**Fig. 6:**
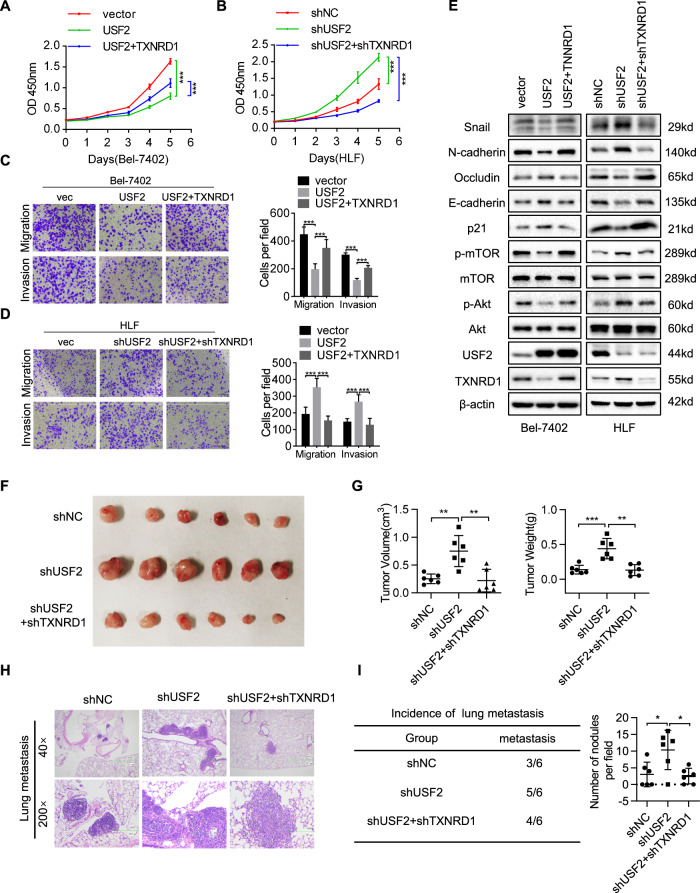
USF2 suppresses the proliferation and metastasis of HCC cells by modulating the TXNRD1. **A** Bel-7402 cells were transiently transfected with USF2 or TXNRD1, cell proliferation abilities were measured by cell growth curve and CCK-8 assay. **B** HLF cells with USF2 knockdown were stably transfected shRNA targeting TXNRD1. Proliferation abilities of indicated cells were measured by cell growth curve and CCK-8 assay. **C**–**D** Cells migration and invasion abilities of indicated cells in (**A** and **B**) were determined by transwell assay. Representative images were shown in left, and the quantitative analysis was shown in right. **E** Cell lysates were prepared and subjected to western blotting for Snail, N-cadherin, E-cadherin, Occludin, p21, p-mTOR, mTOR, p-Akt, Akt, USF2, TXNRD1. β-actin was used as a loading control. **F** Subcutaneous tumors derived from shNC, shUSF2 or shUSF2 + TXNRD1 HLF cells were photographed. **G** Average weight and volume of excised tumors were determined. **H** Lung metastasis in nude mice inoculated with shNC, shUSF2 or shUSF2 + TXNRD1 HLF cells via tail vein for 8 weeks was constructed. Representative HE stained lung tissue sections was presented. **I** Statistical analysis of the incidence of lung metastases is shown (left). The number of visible metastatic nodules in each group is counted in dot chat (right). Data represent the mean ± SD or mean ± SEM. *P < 0.05, **P < 0.01, ***P < 0.001. *P* values were calculated by One-way ANOVA.

To study the functional role of USF2 in HCC, Bel-7402 and Huh7 cells were transiently transfected USF2 or USF2 and TXNRD1. We also established stable USF2 knockdown in HFL cells (Fig. S6A). In keep with above results, genetic downregulation of USF2 significantly increased TXNRD1 expression at the protein and mRNA levels (Fig. S6B). HLF cells with USF2 knockdown were stably transfected shRNA targeting TXNRD1. Functionally, ectopic expression of USF2 significantly suppressed the proliferation, migration and invasion capabilities of these cells, which could be partially restored by TXNRD1-rescued expression (Fig. 6A, C and Fig. S6C, D). USF2 knockdown greatly enhanced the proliferation, migration and invasion capabilities of HLF cells, which could be abolished by TXNRD1 inhibition (Fig. 6B, D). Furthermore, we found that USF2 inhibited the phosphorylation of Akt and mTOR, decreased the expression of snail and N-cadherin, increased the expression of Occludin, E-cadherin and p21. Importantly, TXNRD1-rescued expression partially reversed these expression patterns induce by USF2 (Fig. 6E). An opposite expression trend of these proteins was observed in USF2 downregulated HLF cells with TXNRD1 knockdown or not (Fig. 6E). These findings in aggregate indicate that USF2 inhibits HCC cells proliferation and metastasis and the Akt/mTOR activation, at least in part, depending on TXNRD1 repression. Furthermore, the nude mice injected with shUSF2-transfected HLF cells were found have larger tumor volume and tumor weight than those injected with control cells, while this enhancement by knockdown of USF2 was blocked by knockdown of TXNRD1 (Fig. 6F, G). Lung metastasis model demonstrated that more lung metastasis nodules were observed in the lung of nude mice with HLF-shUSF2 cells injection compared to control cells. However, the lung metastatic nodules were decreased after a further knockdown of TXNRD1 (Fig. 6H, I). Together, the above data indicated that USF2 inhibited the proliferation and metastasis in HCC cells through inhibiting TXNRD1.

### USF2 is downregulated and negatively correlated with TXNRD1 expression and poor prognosis in HCC

**Fig. 7:**
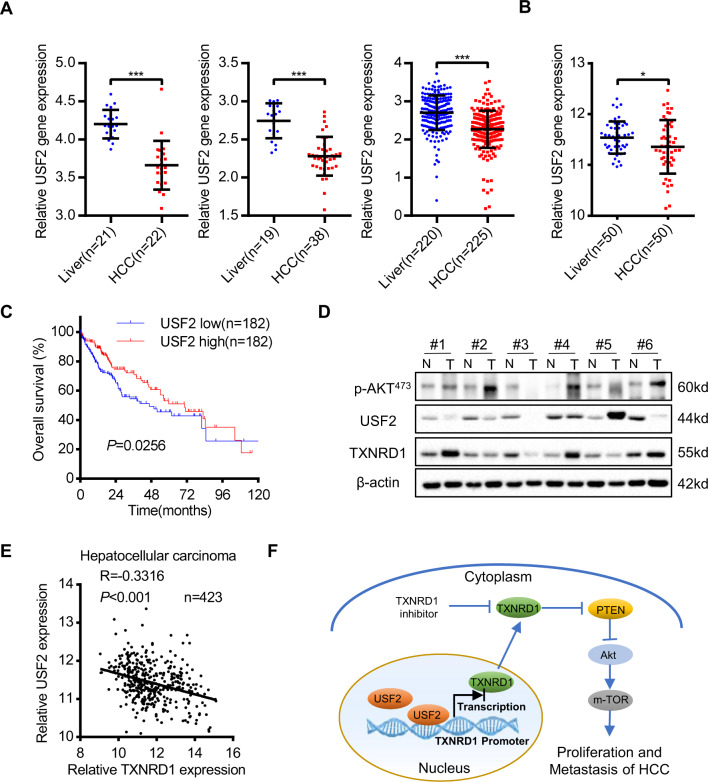
USF2 is downregulated in HCC and negatively associated with TXNRD1 expression. **A** Dot density plots show USF2 mRNA level in normal or tumorous HCC samples determined by microarray analysis. Different datasets from the Oncomine database were analyzed. **B** USF2 expression in 50 paired samples of tumorous tissues and nontumorous adjacent normal liver tissues from patients with HCC in TCGA cohort. **C** Kaplan-Meier analysis for overall survival was performed according to USF2 levels in TCGA cohort. **D** Protein levels of p-Akt, USF2 and TXNRD1 were examined by western blot analysis in six paired samples from patients with HCC. **E** Dot plot showing the correlation between the mRNA levels of TXNRD1 and USF2 in the TCGA LIHC panel. **F** Schematic depiction of mechanisms underlying USF2-mediated upregulation of TXNRD1 contributes HCC proliferation and metastasis via Akt/mTOR activation. Data represent the mean ± SD. *P < 0.05, ***P < 0.001. *P* values were calculated by Student’s t test (**A**), Paired t test (**B**), log-rank test (**D**), Pearson’s correlation coefficient(E).

Given the results from these experiments, we sought to determine the transcriptional regulation of USF2 and TXNRD1 in the clinical setting. To understand whether high TXNRD1 expression is associated with inhibition of USF2-mediated transcriptional program in clinical HCC specimens, we analyzed USF2 levels in the Oncomine and TCGA database. We found that a decreased expression of USF2 in HCC tumor tissues compared with adjacent normal liver tissues (Fig. 7A, B). Furthermore, the overall survival period was significantly longer in USF2 higher expression group than that in USF2 lower expression group (Fig. 7C), suggesting a tumor suppressive role and prognostic value of USF2 in HCC. We also determined the protein expression of TXNRD1, USF2, and phospho-Akt in paired HCC tumor tissues. The result showed that the protein levels of TXNRD1 and phospho-Akt were consistently higher in HCC tumor tissues compared with their adjacent normal tissues, while a consistently opposite expression pattern of USF2 was detected (Fig. 7D). To explore the clinical relevance, we analyzed the association between TXNRD1 expression and USF2 expression in TCGA database. TXNRD1 expression negatively correlated with USF2 expression (Fig. 7E). Intriguingly, correlation analysis revealed that the negative co-expression correlation between TXNRD1 and USF2 was presented in nine clinical cohort of other human malignancies (Fig. S7), as strong clinical evidence of USF2-mediated transcriptional suppression of TXNRD1. Altogether, these results suggest that downregulation of USF2 weakens the transcriptional repression of TXNRD1, contributes to the overexpression of TXNRD1 and poor prognosis in HCC.

## Discussion

In this study, we have identified the role of TXNRD1 in HCC and determined the possible mechanisms involved. Our results demonstrated the TXNRD1 was significantly upregulated in HCC tumor tissues, which was also verified by database analysis. TXNRD1 overexpression correlated with poor survival in HCC patients. Functional studies indicated TXNRD1 knockdown substantially suppressed HCC cell proliferation and metastasis both in vitro and in vivo, and its overexpression showed opposite effects. Mechanistically, TXNRD1 attenuated the interaction between Trx1 and PTEN which resulting in the acceleration of PTEN degradation, thereby activated Akt/mTOR signaling and its target genes which conferred to elevated HCC cell mobility and metastasis. Moreover, USF2 was identified as a transcriptional suppressor of TXNRD1, which directly interacted with two E-box sites in TXNRD1 promoter. USF2 functioned as tumor suppressor through the downstream repression of TXNRD1. Further clinical data revealed negative co-expression correlations between USF2 and TXNRD1.Our data together describe a novel USF2-TXNRD1-Akt/mTOR signaling axis that regulates HCC proliferation and metastasis and provide new insight into how TXNRD1 functions in the progression of HCC (Fig. 7F).

TXNRD1, one of the major redox regulators in mammalian cells, acts in protecting normal cells from oxidative burden, whose primary function in normal cells is to keep Trx1 in the reduced state [32]. Several lines of evidence have supported the idea that TXNRD1 has cancer-preventive effects. TXNRD1 participated in activating the p53 tumor suppressor and promoting other tumor suppressor activities [33], and its downregulation by specific carcinogenic electrophilic compounds resulted in altering some of the cell’s malignancy phenotypes [34]. TXNRD1-deficient mice exposed to a liver carcinogen showed a significantly increased incidence for chemically induced liver cancer [32]. However, high expression level of TXNRD1 have been reported in many cancer cells, including neoplastic liver cells [23, 24], suggesting that the increase in TXNRD1 is important for survival and the promotion of cancer progression. In this study, we further confirmed that high expression of TXNRD1 was observed in HCC and related to poor prognosis. Besides, our data proved that TXNRD1 enhanced the proliferation and metastasis of HCC cells in vivo and in vitro, demonstrating that TXNRD1 promoted HCC progression. It should be emphasized that the two opposing roles of TXNRD1 in cancers, prevention/promotion, is not paradoxical. TXNRD1 functions as maintaining redox homeostasis and decreasing the mutation of normal cells that lead to cancer. Malignant cells often display upregulation of cellular antioxidant systems and exhibit stronger reliance on antioxidant systems counteracting the ROS overproduction accompanying a cancer-specific deregulated metabolism and increased mitogenic signaling [35]. Thus, the dual role of TXNRD1 in cancers might depend on the stage of cancer initiation and development and on varied vulnerabilities of tissues to oxidative stress as well.

PI3K/Akt/mTOR are important kinases activated by many cellular stimuli and regulate fundamental cellular functions, including transcription, translation, proliferation, growth, cell size, metabolism, and motility [36]. Compelling evidences reveal that dysregulation of PI3K/Akt/mTOR signaling contributes to the development of multiple cancers. Thus, alteration of the PI3K/Akt/mTOR pathway is strongly implicated in cancer pathogenesis, and targeting the effectors of this pathway is a promising therapeutic approach [7]. In nonsmall cell lung carcinoma (NSCLC), inhibition of TXNRD1 with siRNAs or its inhibitor, auranofin, sensitized NSCLC cells to the AKT inhibitor MK2206 treatment in vitro and in vivo, and simultaneous inhibition of TXNRD1 and AKT pathways induced robust ROS production, which was involved in c-jun-NH2-kinase (JNK) activation and cell apoptosis [29]. Recent pioneers demonstrated that chaetocin inhibited TXNRD1 and subsequently induced excessive ROS accumulation followed by inactivation of the PI3K/AKT pathway in gastric cancer cells [37]. Similarly, an allylated MAC inhibited gastric cancer growth by increasing ROS through directly binding to and inhibiting TXNRD1, which in turn activated FoxO3a through suppressing Akt [38]. However, whether Akt/mTOR signaling is involved in the TXNRD1-induced proliferation and metastasis of HCC cells was largely unknown. Here, we reported that TXNRD1 increased the cell proliferation, migration and invasion abilities and induced activation of Akt/mTOR signaling, which could be abolished by the AKT inhibitor MK2206 treatment, implying that Akt/mTOR signaling was at least partially involved in TXNRD1 induced HCC progression. Nevertheless, the regulatory molecular mechanism of TXNRD1 on Akt/mTOR pathway in HCC has not been determined.

It is well known that PI3K/Akt pathway is negatively controlled by a group of lipid phosphatases of which PTEN is the main representative. As an antagonist of PI3K signaling, PTEN dephosphorylates PIP3, so impaired PTEN functions by genetic, epigenetic, or proteic alterations that leads to PIP3 accumulation in cells and to uncontrolled activation of its downstream Akt signals [13, 39]. There are a number of well-established and documented regulatory mechanisms acting to modulate PTEN gene expression and protein abundance, stability and activity, including epigenetic loss or mutation of PTEN; transcriptional regulations; post-transcriptional regulation by microRNA, competitive endogenous RNA, and long non-coding RNA; protein modification by phosphorylation, oxidation, acetylation, ubiquitination and SUMOylation; aberrant localization of PTEN [14, 40]. PTEN is susceptible to oxidation since it harbors a cysteine residue at the catalytic site like other protein tyrosine phosphatases [41]. For instance, PTEN oxidation by H_2_O_2_ facilitates disulphide bond formation between the catalytic Cys^124^ and Cys^71^ residues, resulting in a conformational change, which alters the PTEN substrate binding site and leads to loss of PTEN phosphatase activity [42]. Reduced Trx1 binds to PTEN through a disulfide bond between the Cys^32^ of Trx1 and Cys^212^ of the C2 domain of PTEN, which results in an inhibition of PTEN lipid phosphatase activity and an increase in levels of constitutive phosphor-Ser^473^ Akt [30]. In this study, TXNRD1 weakened the interaction between Trx1 and PTEN, suggesting that TXNRD1 regulated the PTEN-Trx1 interaction in redox-dependent manner. This is concordant with previous reports that Trx1 bounded preferentially to oxidized PTEN bait protein and contributed to the reactivation of PTEN via reduction of disulfide exchange between Cys^71^ and Cys^124^ of PTEN with a thiol-disulfide exchange mechanism [42–44]. Interestingly, it has also been shown that TXNRD1 overexpression accelerates the degradation of PTEN and consequently induces to decreased protein level of PTEN. Overall, these findings imply that TXNRD1 promotes HCC proliferation and metastasis by activating Akt/mTOR signaling via PTEN regulation, additional studies should be required to fully understand the dynamics mechanisms of TXNRD1-mediated PTEN regulation.

Since our above results of clinical data showed that TXNRD1 was significantly upregulated at both protein and mRNA levels in HCC tissues, we next explored the upstream mechanism responsible for the regulation of TXNRD1 overexpression in HCC at the transcriptional level. We identified USF2 as a potential transcription repressor of TXNRD1 using database screening (CIS-BD, Jaspar and Promo) of promoter analysis combined with luciferase reporter assays. USF2 belongs to the basic helix-loop-helix-leucine zipper transcription factor family and act as either homodimer of heterodimer by binding to E-boxes of the DNA-core sequence (5’-CANNTG-3’) in their target genes [31, 45]. Although knowns as a transcriptional activator [46–48], USF2 also functions as a transcriptional repressor. USF2 overexpression in cultured human trophoblasts markedly inhibits endogenous CYP19 expression, differentiation of cultured human trophoblast cells, and CYP19 promoter activity [49]. Gel mobility shift experiments show binding of the transcription factors USF2 to the site -114 to -119 of the MCT1 promoter, the USF2 appears to have a repressor role on the MCT1 promoter with the use of site-directed mutagenesis and promoter activity in Caco-2 cells [50]. Moreover, active USF2 inhibits cellular transformation by preventing transcriptional repression by c-Myc [51], reduces the DNA binding activity of c-Maf [52], restrains C/EBP-mediated transcriptional regulation of the RIIbeta subunit of cAMP-dependent protein kinase C/EBP [53], demonstrating that USF2 could regulate the transcriptional activity of other transcription factors. Here, our data showed that USF2 attenuated TXNRD1 promoter luciferase activity by binding to the E-box2 and E-box3 sites, since it failed to inhibit the reporter in which the E-box2 and E-box3 sites were mutant in the context of the TXNRD1 promoter-reporter construct. ChIP assay further confirmed that USF2 was co-recruited to the TXNRD1 E-box2 and E-box3 elements, indicating that USF2 is a crucial transcription factor in the repression of TXNRD1. Our study is the first to reveal the transcriptional regulation relationship between USF2 and TXNRD1.

USF2 has been reported to implicate in several cellular processes, such as embryogenesis, metabolism and cancer development. Interestingly, the data with respect to the role of USF2 in tumor development are conflicting suggesting that it has a dual role as either tumor promoter or suppressor. Earlier result revealed that lack of USF2 function caused increased cell growth in Saos-2 osteosarcoma cells [54]. Moreover, chen et al. reported that USF2 was downregulated in human prostate cancer tissues, and USF2 expression inhibited the malignant properties of prostate cancer cells [55]. Consistent with these observations, we uncovered that deficient of USF2 gained an overall advantage in proliferation, migration and invasion, AKT phosphorylation. Rescue of the USF2-deficient cells with TXNRD1 knockdown reversed these effects. Clinical data demonstrated that low expression of USF2 in HCC tissue was associated with poor prognosis. However, our present data are inconsistent with earlier findings reporting that USF2 expressions are significantly increased in HCC [56], likely due to the extremely small sample size they analyzed. Further opposition comes from other investigation showing that USF2 inhibits the transcriptional activity of Smurf1 and Smurf2 to promote breast cancer cells proliferation, migration and invasion [57]. These apparent opposing roles of USF2 in cancers is plausible that USF2 appears to play crucial roles as either transcriptional activators or transcriptional repressors of various genes, also cooperates with other factors in tissue- and/or a stimulus-specific manner to activate or inhibit the transcription of identical genes [58]. Another possible explanation is that targeted genes whose expression is regulated by USF2 may be more important in determining the tumor-suppressive or tumor-promotive functions of USF2. Accordingly, further investigations elucidating the complex regulatory mechanisms of USF2 may help us to eliminate the discrepancy.

In summary, we provide novel evidence that USF2-mediated upregulation of TXNRD1 contributes to hepatocellular carcinoma progression by activating Akt/mTOR signaling. A better understanding of the roles and mechanisms of TXNRD1 in HCC may lead to new therapeutic strategies in HCC treatment.

## Supplementary information

Supplementary Tables

Supplementary materials and methods

Supplementary Figure legends

Figure S1

Figure S2

Figure S3

Figure S4

Figure S5

Figure S6

Figure S7

Original Data File

## Data Availability

All data generated or analyzed in this study are included in this paper and can be obtained from the corresponding author according to formal requirement.
