# Supplementary material for: USF2-mediated upregulation of TXNRD1 contributes to hepatocellular carcinoma progression by activating Akt/mTOR signaling
Source: Cell Death Dis. 2022 Nov 1;13(11):917. doi: 10.1038/s41419-022-05363-x (PMC9626593; doi:10.1038/s41419-022-05363-x)
Supplement: Supplementary file 1 — Supplementary Tables [file 41419_2022_5363_MOESM1_ESM.docx]

| **Table S1. List of primers used in this study** | | |
| --- | --- | --- |
| Primer | Forward Primer (5′-3′) | Reverse Primer (5′-3′) |
| **Primers for luciferase assay** | | |
| pGL4.17-2000 | ctggcctaactggccggtaccATTATTATTAGGTATTTGGGGGACAAA | ccagatcttgatatcctcgagTTAGAATCCTGGTGCCTGGC |
| pGL4.17-1000 | ctggcctaactggccggtaccATGACCCTGGTTCATGGGTCA |  |
| pGL4.17-500 | ctggcctaactggccggtaccTCCTTCCTTCATTCAGTTATTATGTATTT |  |
| pGL4.17-200 | ctggcctaactggccggtaccTTTCAAAAGCCTAATAACTTCCTTTAGG |  |
| pGL4.17-mut1 | TAGCacttgtTGGTGGTGCATGCCTGTAGTCCC | CACCACCAacaagtGCTAATTTTTTGTATTTTTAGTAGAAATGG |
| pGL4.17-mut2 | GGACTCCAGAATacttgtAAACACTCATGTCAATAATCAAACACTC | acaagtATTCTGGAGTCCCTAGAACAGAGAATG |
| pGL4.17-mut3 | AGTAGCTGacttgtGCTGGGTGCAGCTGGAGTT | CAGCacaagtCAGCTACTAAGGTGAGCCATGCT |
| **Primers for CDS cloning** | | |
| pcDNA3.1-Flag-LEF1 | gatgacgatgacaagaagcttATGCCCCAACTCTCCGGA | tgctggatatctgcagaattcTCAGATGTAGGCAGCTGTCATTCT |
| pcDNA3.1-Flag-ELK1 | gatgacgatgacaagaagcttATGGACCCATCTGTGACGCTG | tgctggatatctgcagaattcTCATGGCTTCTGGGGCCC |
| pcDNA3.1-Flag-TBP | gatgacgatgacaagaagcttATGGATCAGAACAACAGCCTGC | tgctggatatctgcagaattcTTACGTCGTCTTCCTGAATCCC |
| pcDNA3.1-Flag-HINF1A | gatgacgatgacaagaagcttATGGTTTCTAAACTGAGCCAGCTG | tgctggatatctgcagaattcTTACTGGGAGGAAGAGGCCAT |
| pcDNA3.1-Flag-FOS | gatgacgatgacaagaagcttATGATGTTCTCGGGCTTCAACG | tgctggatatctgcagaattcTCACAGGGCCAGCAGCGT |
| pcDNA3.1-Flag-POU2F2 | gatgacgatgacaagaagcttATGGTTCACTCCAGCATGGG | tgctggatatctgcagaattcTCAGGCCTGACAAGCCGA |
| pcDNA3.1-Flag-GATA2 | gatgacgatgacaagaagcttATGGAGGTGGCGCCGGAG | tgctggatatctgcagaattcCTAGCCCATGGCGGTCACC |
| pcDNA3.1-Flag-TCF4 | gatgacgatgacaagaagcttATGCATCACCAACAGCGAATG | tgctggatatctgcagaattcTTACATCTGTCCCATGTGATTCG |
| pcDNA3.1-Flag-FOXP3 | gatgacgatgacaagaagcttATGGATGATGATATCGCCGCGC | tgctggatatctgcagaattcTCAGGGGCCAGGTGTAGGGTTGG |
| pcDNA3.1-Flag-USF2 | gatgacgatgacaagaagcttATGGACATGCTGGACCCGG | tgctggatatctgcagaattcTCACTGCCGGGTGCCCTC |
| pLenti-Flag-TXNRD1 | CATAGAAGACACCGACTCTAGAcgccaccatgaacggccctgaagatct | CTTTGTAGTCAGCCCGGGATCCacctcagcagccagcctgga |
| pLenti-Flag-USF2 | atagaagacaccgactctagagccacc ATGGACATGCTGGACCCGG | tttgtagtcagcccgggatccCTGCCGGGTGCCCTCGCC |
| **Primers for qRT-PCR** | | |
| TXNRD1 | ATGGCAAGAAGGTGATGG | GCAGTAAGGCAAGGAGAA |
| β-actin | CATGTACGTTGCTATCCAGGC | CTCCTTAATGTCACGCACGAT |
| PTEN | TTTGAAGACCATAACCCACCAC | ATTACACCAGTTCGTCCCTTTC |
| USF2 | CCCGGACACACCCTTACTCT | GCTCCACTTCGTTGTGCTG |
| **Primers for ChIP assay** | | |
| TXNRD1 promoter  USF1 binding site 1 | AGTGGCTCACACCTGTAATCC | GTCTCTTGCTCTGTTGCTCTG |
| TXNRD1 promoter  USF1 binding site 2 | CGTACTAGATCCATTCTCTGTTCT | CCTGTAAGGTGATTATTGACATGAG |
| TXNRD1 promoter  USF1 binding site 3 | CCTTCCTTCCTTCCTTCATTCAG | GAGCACCTCTTCCTTATTAGATTCT |

| **Table S2. The correlation between TXNRD1 expression and clincopathological features in HCC patients** | | | | |
| --- | --- | --- | --- | --- |
| Clinical variables | No. of patients | TXNRD1 expression level | | *P* value |
|  | n=115 | Low(n=37) | High(n=78) |  |
| Age(years) |  |  |  | 0.068 |
| <60 | 91 | 33(89.2%) | 58(74.4%) |  |
| ≥60 | 24 | 4(10.8%) | 20(25.6%) |  |
| Gender |  |  |  | 0.507 |
| Male | 97 | 30(81.1%) | 67(85.9%) |  |
| Female | 18 | 7(18.9%) | 11(14.1%) |  |
| HBsAg |  |  |  | 0.435 |
| Positive | 89 | 27(73.0%) | 62(79.5%) |  |
| Negative | 26 | 10(27.0%) | 16(20.5%) |  |
| ALT(U/L) |  |  |  | 0.416 |
| ≤40 | 78 | 27(73.0%) | 51(65.4%) |  |
| >40 | 37 | 10(27.0%) | 27(34.6%) |  |
| AST(U/L) |  |  |  | 0.371 |
| ≤40 | 78 | 23(62.2%) | 55(70.5%) |  |
| >40 | 37 | 14(37.8%) | 23(29.5%) |  |
| AFP(ng/ml) |  |  |  | 0.283 |
| <20 | 39 | 10(27.0%) | 29(37.2%) |  |
| ≥20 | 76 | 27(73.0%) | 49(62.8%) |  |
| Child-Pugh Class |  |  |  | 0.908 |
| A | 102 | 33(89.2%) | 69(88.5%) |  |
| B | 13 | 4(10.8%) | 9(11.5%) |  |
| Liver cirrhosis |  |  |  | 0.028 |
| No | 21 | 11(29.7%) | 10(12.8%) |  |
| Yes | 94 | 26(70.3%) | 68(87.2%) |  |
| Tumor size(cm) |  |  |  | 0.802 |
| ≤5 | 36 | 11(29.7%) | 25(32.1%) |  |
| >5 | 79 | 26(70.3%) | 53(67.9%) |  |
| Tumor number |  |  |  | 0.883 |
| Single | 88 | 28(75.7%) | 60(76.9%) |  |
| Multiple | 27 | 9(24.3%) | 18(23.1%) |  |
| Vascular invasion |  |  |  | 0.614 |
| Yes | 25 | 7(18.9%) | 18(23.1%) |  |
| No | 90 | 30(81.1%) | 60(76.9%) |  |
| Tumor differentiation | |  |  | 0.243 |
| Well/Moderate | 68 | 19(51.4%) | 49(62.8%) |  |
| Poor | 47 | 18(48.6%) | 29(37.2%) |  |
| TNM stage |  |  |  | 0.452 |
| I | 77 | 23(62.2%) | 54(69.2%) |  |
| II-III | 38 | 14(37.8%) | 24(30.8%) |  |

| **Table S3. List of antibodies used in this study** | | | | | | | |
| --- | --- | --- | --- | --- | --- | --- | --- |
| Antibody | WB | IHC | IF | IP | Specificity | Company | Catalog Number |
| TXNRD1 | 1:1000 | 1:100 | / | / | Mouse monoclonal | Santa Cruz Biotechnology | sc-28321 |
| β-actin | 1:1000 | / | / | / | Rabbit polyclonal | Cell Signaling Technology | #4967 |
| Ki-67 | / | 1:200 | / | / | Rabbit monoclonal | Abcam | ab16667 |
| p-mTOR | 1:1000 | / | / | / | Rabbit monoclonal | Cell Signaling Technology | #5536 |
| mTOR | 1:1000 | / | / | / | Rabbit monoclonal | Cell Signaling Technology | #2983 |
| p-Akt(473) | 1:1000 | / | / | / | Rabbit monoclonal | Cell Signaling Technology | #4060 |
| Akt | 1:1000 | / | / | / | Rabbit monoclonal | Cell Signaling Technology | #4685 |
| p21 | 1:1000 | / | / | / | Rabbit monoclonal | Cell Signaling Technology | #2947 |
| ZO1 | 1:1000 | / | / | / | Rabbit polyclonal | Proteintech | 21773-1-AP |
| Snail | 1:1000 | / | / | / | Rabbit polyclonal | Proteintech | 13099-1-AP |
| Occludin | 1:1000 | / | / | / | Rabbit monoclonal | Cell Signaling Technology | #91131 |
| N-cadherin | 1:1000 | / | 1:100 | / | Mouse monoclonal | BD Biosciences | 610921 |
| E-cadherin | 1:1000 | / | 1:100 | / | Mouse monoclonal | BD Biosciences | 610182 |
| cyclinD1 | 1:1000 | / | / | / | Rabbit monoclonal | Cell Signaling Technology | #55506 |
| Trx1 | 1:1000 | / | / | 1:50 | Mouse monoclonal | Santa Cruz Biotechnology | sc-166393 |
| PTEN | 1:1000 | / | / | / | Rabbit monoclonal | Cell Signaling Technology | #9188 |
| Flag | 1:1000 | / | / | / | Mouse monoclonal | Sigma-Aldrich | F1804 |
| USF2 | 1:1000 | / | / | 1:100 | Mouse monoclonal | Santa Cruz Biotechnology | sc-293443 |
