# Supplementary material for: USF2-mediated upregulation of TXNRD1 contributes to hepatocellular carcinoma progression by activating Akt/mTOR signaling
Source: Cell Death Dis. 2022 Nov 1;13(11):917. doi: 10.1038/s41419-022-05363-x (PMC9626593; doi:10.1038/s41419-022-05363-x)
Supplement: Supplementary file 2 — Supplementary materials and methods [file 41419_2022_5363_MOESM2_ESM.docx]

**Supplementary materials and methods**

**Cell lines and culture**

HL-7702, HepG2, Huh7, Sk-Hep1, Bel-7402, HLE, HLF, Hep3B and Alex cell lines were purchased from China Center for Type Culture Collection (CCTCC, Wuhan, China). MHCC-97H and HCC-LM3 cell lines were obtained from Liver Cancer Institute, Zhongshan Hospital, Fudan University, Shanghai, China. 293T cells were purchased from the American Type Culture Collection. Cells were cultured with complete DMEM containing 10% FBS and antibiotics (100 μg/ml streptomycin and 100 units/ml penicillin). Cells were grown at 37˚C in a humidified atmosphere of 5% CO2. All cell lines were tested negative for mycoplasma contamination. None of the cell lines have been authenticated.

**Plasmids and constructs**

All plasmids for constructs and plasmids for lentivirus package listed in the paper were purchased from Addgene. Human TXNRD1 and transcription factor genes were amplified from the Han’s Lab cDNA library and subsequently cloned into pLenti-CMV-GFP or pcDNA3.1 vector. Human TXNRD1 promoter region was amplified from genome DNA of Huh7 cells and subsequently cloned into pGL4.17 vector. The shRNA sequences of TXNRD1 and USF2 were as follows: human TXNRD1, 5′-GCTGGATTTCTTGCTGGTATT-3′, 5′-CGTCAAGAGATAACAACAAAT-3′, 5′-CCTGCAAGACTCTCGAAATTA-3′; human USF2, 5′-TCCTCCACTTGGAAACGG

TAT-3′, 5′-GACACACCCTTACTCTCCAAA-3′, 5′-TCCAGACTGTAACGCAGACA

A-3′. TXNRD1 promoter constructions with fragment and E-box mutations were made by performing truncation and site mutagenesis according to the manufacture’s manual. (Vazyme, Mut Express MultiS Fast Mutagenesis Kit). All constructs were confirmed by sequencing.

**Transfection and lentivirus production**

Transfections were performed using Lipofectamine 2000 according to the manufacture’s instruction. For lentivirus production, pLKO.1 vector and pLKO.1-sh or pLenti-CMV-GFP vector and pLenti-CMV-GFP-targeted-gene (6 μg), psPAX2 (4.5μg), pMD2.G (1.5 μg) were co-transfected into 293T cells. The virus-containing supernatants were collected and filtered 48 h after transfection. Freshly made virus supernatants supplemented with 10 μg/ml polybrene were added to exponentially growing HCC cells. Stable overexpression or knockdown cells were achieved by 1-week puromycin (5 μg/ml) selection. The infection efficiency of lentivirus on cells was determined by western blotting analysis.

**Western blotting**

Cells or tissues were prepared using 1x RIPA buffer supplemented with 1% EDTA-free protease inhibitor cocktail (Roche) and 1% phosphatase inhibitor cocktail (Sigma). After centrifugation, protein contents were determined using the BCA method (Sigma) for same concentration and denatured in 4×NuPAGE LDS buffer. For Western blots, equal amounts of protein were separated by 8-12% sodium dodecyl sulfate-polyacrylamide gel electrophoresis (SDS-PAGE) and transferred to polyvinylidene fluoride (PVDF) membranes (Millipore). Transferred blots were blocked in 5% non-fat milk for 2 h, incubated with primary antibody at 4℃ overnight, and followed by horseradish peroxidase (HRP)-linked secondary antibody incubation for 2 h. The enhanced ECL was used to detect the HRP signal and blot images were visualized by Bio-Rad ChemiDoc system. The information for specific antibodies is listed in Supplementary Table S3.

**IHC staining**

Tissue sections were deparaffinized in xylene, rehydrated with ethanol and subjected to antigen retrieval in boiling citrate buffer for 15 min. After peroxide block, the section was incubated with primary antibody at 4℃ overnight. Primary antibodies were tittered against normal tissues to determine the optimal dilution before using the tissue microarray. The section was then treated with secondary antibody (Dako, Denmark) for 1 hour at room temperature. The peroxidase reaction was developed with diaminobenzidine (DAB, Dako, Denmark). Staining images were visualized using 3DHIESTECH scan system and software. Cell-based average integrated option density (IOD) for protein expression was analyzed by Image-Pro Plus 6.0 software (Media Cybernetics Inc, Bethesda, USA). The data was assessed separately by two pathologists and discrepancies were resolved by consensus.

**Cell proliferation assay**

Cells were seeded in 96-well microplate at the density of 1000 cells per well. After cultured for 24, 48, 72, 96 and 120 h, 100μl of 10% Cell Counting Kit-8(CCK-8, Dojindo Laboratories) solution was added for 2 h at 37℃. The optical density (OD) value was measured at 450 nm using an enzyme-linked immunosorbent assay plate reader (Bio-Tek Elx 800, USA).

**Colony formation assay**

HCC cells were seeded in 6-well plate at the density of 800 cells per wells. After 14 days, the colonies were fixed in 4% paraformaldehyde and stained with 1% crystal violet for 15 minutes. The plates were taken picture and the number of colonies larger than 100 μm in diameter were counted.

**Transwell assay**

Cell migration and invasion abilities were determined by Transwell assay. In brief, cells in 200 μl of DMEM were placed in to the upper chamber of transwell inserts (8 μm, Corning, USA) for migration assay or Matrigel-coated transwell inserts for invasion assay, and 650 μl of 10% FBS-containing DMEM in the absence or presence of Stattic was added to the lower chamber. After incubation for 24 or 48 h, cells on the upper surface of the inserts were removed with a cotton swab. The migrative or invasive cells were fixed and stained. Photographs of six random fields across three replicate wells were captured for quantification analysis.

**Thioredoxin Reductase activity detection**

Thioredoxin Reductase activity was measured using the Thioredoxin Reductase Colorimetric Assay Kit (Cayman CHEMICAL,USA) according to the manufacture’s instructions. In brief, 1×10^8^ cells were homogenized in 5ml cold PBS buffer, centrifuged for 15 minutes at 4℃, and the supernatant was removed for assay. Then, the reactions were initiated by adding 20 μl NADPH and 20 μl DTNB and followed by shaking the microtiter plated for 10 seconds to mix. The colorimetric absorbance was read once every minute at 410nm using a plate reader to obtain at least five time points. The slop rate of the linear portion of the curve was regarded as the Thioredoxin Reductase activity.

**RNA isolataion and quantitative real-time PCR**

Total RNA was extracted from cells using Trizol regent (Invitrogen), and the concentration of isolated RNA and the ratio of absorbance at 260nm to 280nm were measured with NanoDrop One/Oneᶜ spectrophotometer (Thermo). cDNA was generated from 2 μg total RNAs using a FastQuant cDNA Synthesis Kit or (TIANGEN,Beijing, China). Quantitative real-time PCR was carried out on a CFX Connect™ Real-Time PCR Detection Systemb (Bio-Rad, Hercules, CA, USA) with SuperReal PreMix Plus (SYBR Green, TIANGEN, Beijing, China) according to the manufacture’s instruction. The quantity of mRNA was calculated using ΔΔCt method and GAPDH were used as a reference. All reactions were performed as triplicates. The primer sequences are listed in Supplementary Table S1.

**Immunofluorescence staining**

Cells were grown on coverslips in a 24-well culture plate, fixed in 4% paraformaldehyde for 15 min, permeabilized with 0.1% Triton X-100 for 15 min and blocked with 5% bovine serum albumin for 2 h. Cells were then incubated with primary antibodies at 4˚C overnight, followed by incubation with Alexa Fluor-conjugated secondary antibodies (Invitrogen, Carlsbad, CA) for 1 h. Finally, nuclei were counterstained with DAPI (Sigma) for 5 min in dark. Images were visualized by an inverted fluorescence microscope on a Nikon Digital ECLIPSE C1 system (Nikon Corporation).
