# Supplementary material for: USF2-mediated upregulation of TXNRD1 contributes to hepatocellular carcinoma progression by activating Akt/mTOR signaling
Source: Cell Death Dis. 2022 Nov 1;13(11):917. doi: 10.1038/s41419-022-05363-x (PMC9626593; doi:10.1038/s41419-022-05363-x)
Supplement: Supplementary file 3 — Supplementary Figure legends [file 41419_2022_5363_MOESM3_ESM.docx]

**USF2-mediated upregulation of TXNRD1 contributes to hepatocellular carcinoma progression by activating AKT/mTOR signaling**

**Figure S1. TXNRD1 is enriched in HCC and associated with poor prognosis of HCC patients in the database.**

(A) TXNRD1 mRNA levels were compared between HCC tumor and non-tumor tissues in the three cohorts from the Oncomine database. *P* value was calculated by t test. (B) The expression of TXRND1 was significantly higher in 50 HCC tumor tissues than those in 50 adjacent non-tumor tissues in the TCGA database. *P* value was calculated by Paired t test. (C) Kaplan –Meier analysis was used to compared the overall survival of the 360 patients stratified by high or low TXNRD1 expression in the TCGA database. *P* value was calculated by log-rank test.

**Figure S2. Construction of cell lines with TXNRD1 knockdown and overexpression.**

(A) Endogenous TXNRD1 protein expression in several HCC cell line and the normal L02 cell line was detected by western blotting. β-actin was used as the control. (B) Western blot analysis confirmed the knockdown efficiency of TXNRD1 by shTXNRD1 in Bel-7402 and MHCC-97H cell lines and ectopic expression of TXNRD1 in Alex and HLF cell lines. (C) The enzyme activity of TXNRD1 was examined in the indicated cell line with TXNRD1 knockdown or overexpression. Data represent the mean±SEM. ***, *P*<0001, One-way ANOVA or Student’s t test.

**Figure S3. TXNRD1 promote tumor growth and metastasis of HCC cells *in vivo.***

(A) HLF cells with TXNRD1 overexpression or control were injected into the flank of nude mice. Images of tumors excised from six nude mice at 5 weeks after inoculation were taken. (B) Dot plots show the volume and weight of indicated tumors.(C) Photos of lung metastases and mice lung were taken after 8 weeks of tail vein injection. Data represent the mean±SEM. * *P*<0.05, Student’s t test.

**Figure S4. TXNRD1 enhances the proliferation and metastasis of HCC through the AKT-mTOR signaling pathway.**

1. Cells migration and invasion abilities in Bel-7402-shCon and Bel-7402-shTXNRD1 cells treated with SC79 or DMSO were determined by transwell assay. Representative images were shown. (B) Cells migration and invasion abilities in HLF-vector and HLF-TXNRD1 cells with MK2206 or DMSO treatment by transwell assay. Representative images were shown. (C) Proliferation abilities of Alex-vector and Alex-TXNRD1 cells treated with MK2206 or DMSO were assessed by cell growth curve and CCK-8 assay. (D) Transwell assay was used to compare cells migration and invasion abilities in Alex-vector and Alex-TXNRD1 cells with MK2206 or DMSO treatment. Representative image (upper) and summary bar chart (lower) are shown. (E) After treatment with MK2206 for 24 hours in Alex-vector and Alex-TXNRD1 cells, protein levels of indicated makers were analyzed by western blotting. (F) Fold-changes in mRNA levels of TXNRD1 and PTEN in the Bel-7402 and HLF cells. Data are shown as mean ± SEM. ns, not significant; ****P* < 0.001. *P* values were calculated by Student’s t test.

**Figure S5. USF2 acts as a transcription factor to inhibit TXNRD1 expression.**

(A) TXNRD1 and USF2 protein expression in several HCC cell line and the normal L02 cell line was detected by western blotting. β-actin was used as the control. (B) Co-expression correlation analysis of TXNRD1 and USF2 protein in cell lines of A. (C) Fragments of the TXNRD1 promoter are shown. Relative luciferase activities of pGL4.17 containing TXNRD1 promoter truncations in Huh7 cells exposed to USF2 or control vector were measured by dual-luciferase assays. (D) Schematic E-box mutations of the TXNRD1 promoter are shown. Relative luciferase activities of pGL4.17 containing E-box site mutations in Huh7 cells exposed to USF2 or control vector were measured by dual-luciferase assays. Data are shown as mean ± SEM. ns, not significant; ****P* < 0.001. *P* values were calculated by Pearson’s correlation coefficient (B) or Student’s t test (C and D).

**Figure S6. USF2 suppresses the proliferation and metastasis of HCC cells by modulating the TXNRD1.**

1. Western blot and qRT-PCR were conducted to confirm the knockdown efficiency of USF2 by shUSF2 in HLF cells. (B) Western blot and qRT-PCR analysis of TXNRD1 and USF2 following stably USF2 knockdown in HLF cells. (C) Huh7 cells expressing USF2 were transiently expressed TXNRD1 or control vector, cell proliferation abilities were measured by cell growth curve and CCK-8 assay. (D) Cells migration and invasion abilities of indicated cells were determined by transwell assay. Representative images were shown in left, and the quantitative analysis was shown in right. (E) Photos of lung metastases and mice lung were taken after 8 weeks of tail vein injection. Data represent the mean±SEM. ***P* < 0.01,****P* < 0.001. *P* values were calculated by Student’s t test (A and B) or One-way ANOVA (D).

**Figure S7. USF2 negatively correlates with TXNRD1 in other malignancies.** Dot plots show the negative correlation between USF2 and TXNRD1 in the nine human malignancies panels from TCGA database. *P* values were calculated by Pearson’s correlation coefficient.
