# Supplementary figures and images for: USF2-mediated upregulation of TXNRD1 contributes to hepatocellular carcinoma progression by activating Akt/mTOR signaling
Source: Cell Death Dis. 2022 Nov 1;13(11):917. doi: 10.1038/s41419-022-05363-x (PMC9626593; doi:10.1038/s41419-022-05363-x)

**Figure S1**

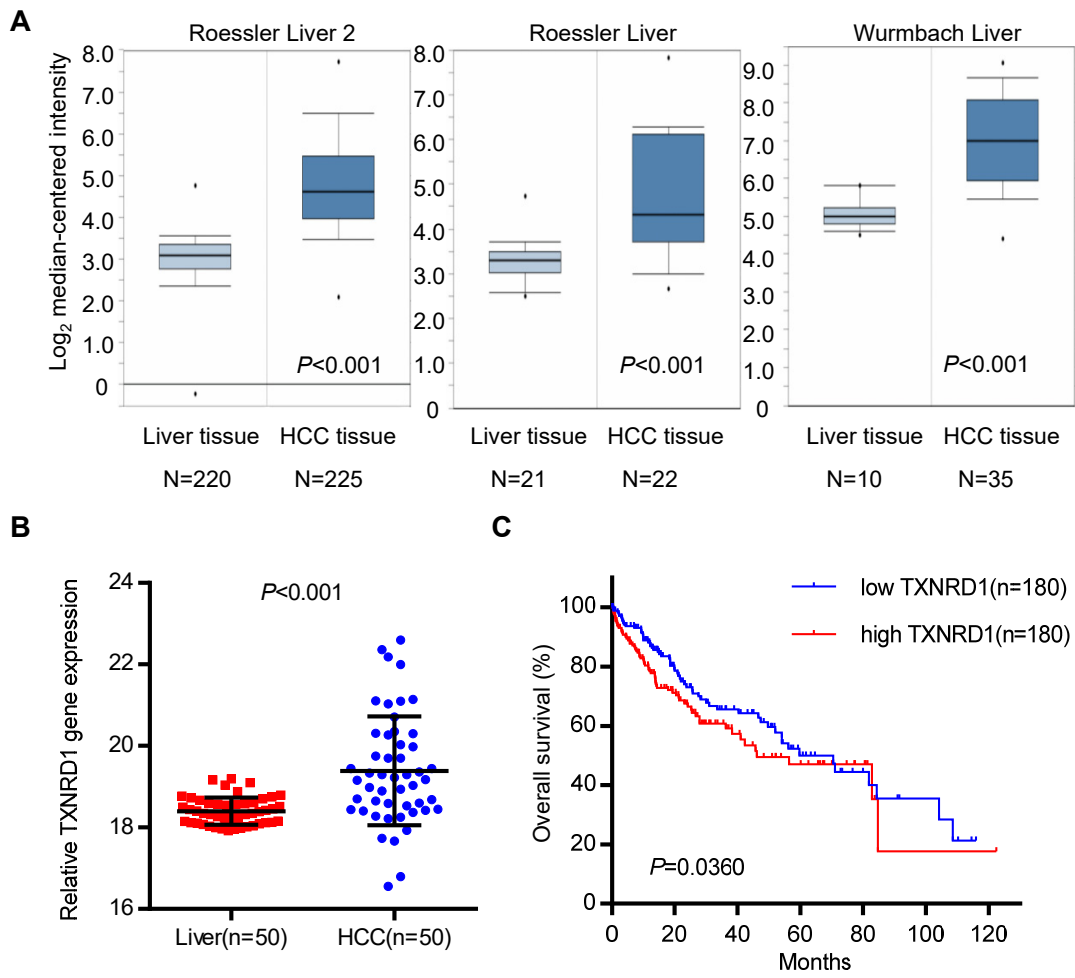

Supplement: Supplementary file 4 — Figure S1 [file 41419_2022_5363_MOESM4_ESM.pdf]

Figure S2

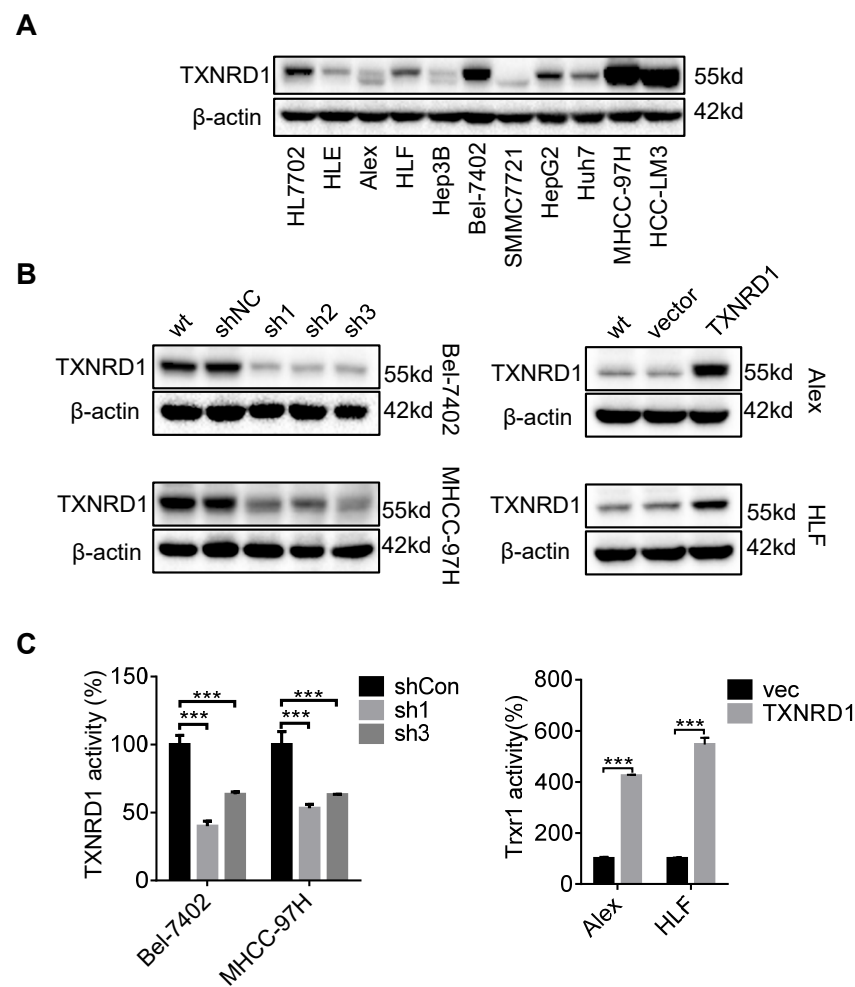

Supplement: Supplementary file 5 — Figure S2 [file 41419_2022_5363_MOESM5_ESM.pdf]

Figure S3

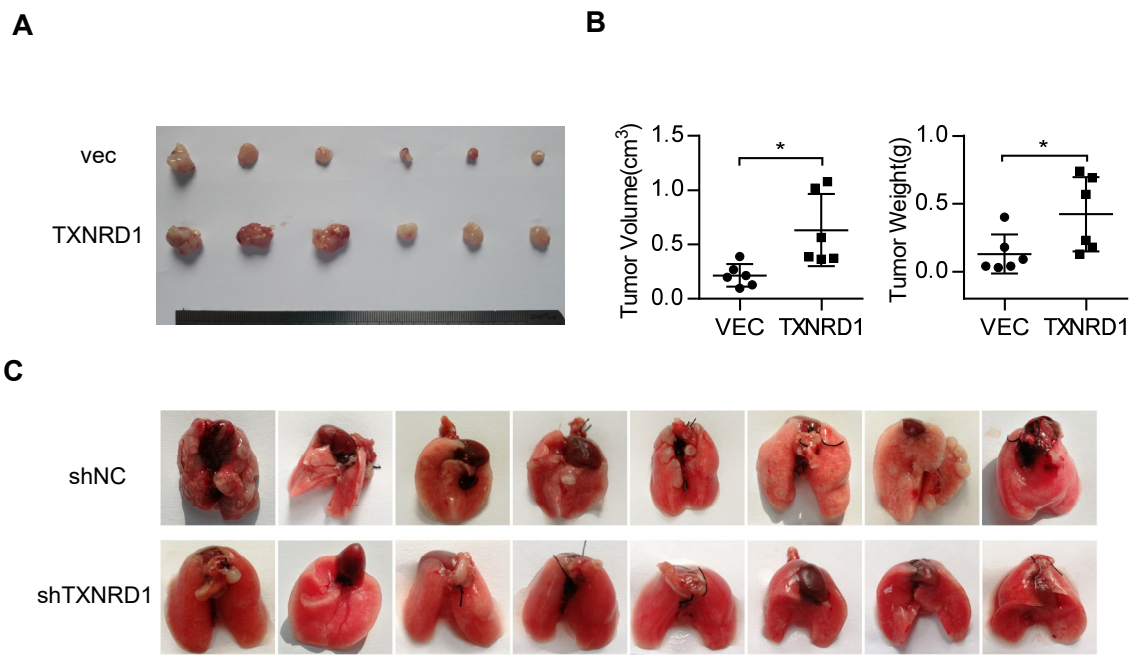

Supplement: Supplementary file 6 — Figure S3 [file 41419_2022_5363_MOESM6_ESM.pdf]

**Figure S4**

**A**

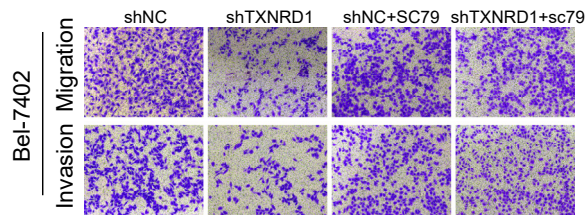

**B**

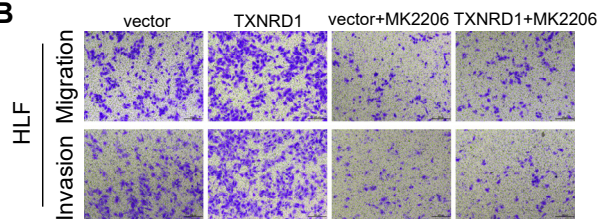

**D**

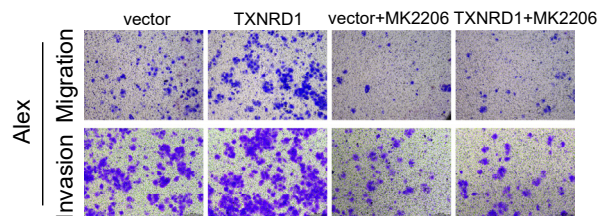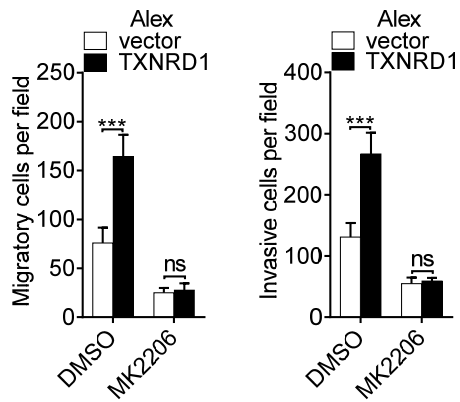

**F**

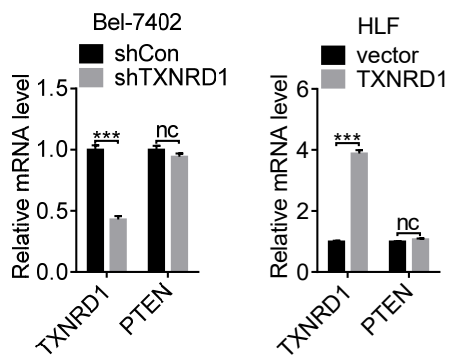

**C**

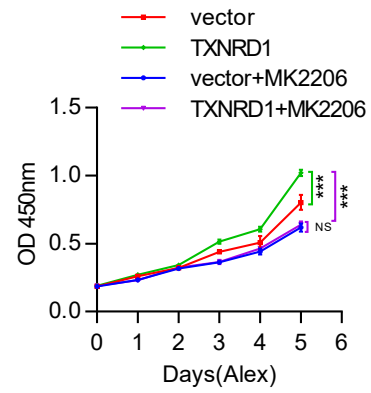

**E**

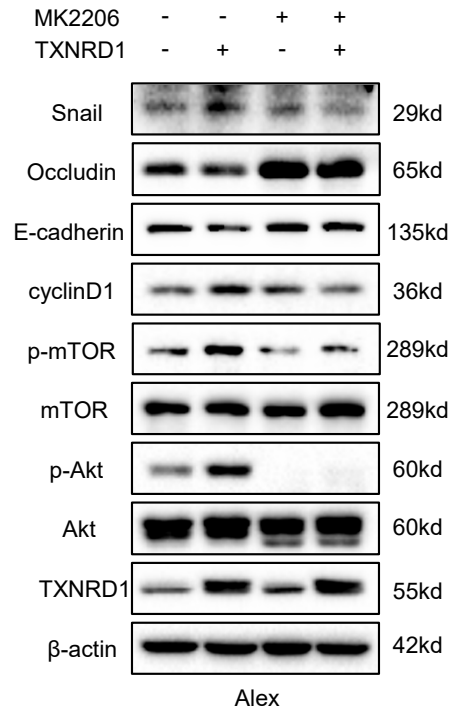

Supplement: Supplementary file 7 — Figure S4 [file 41419_2022_5363_MOESM7_ESM.pdf]

Figure S5

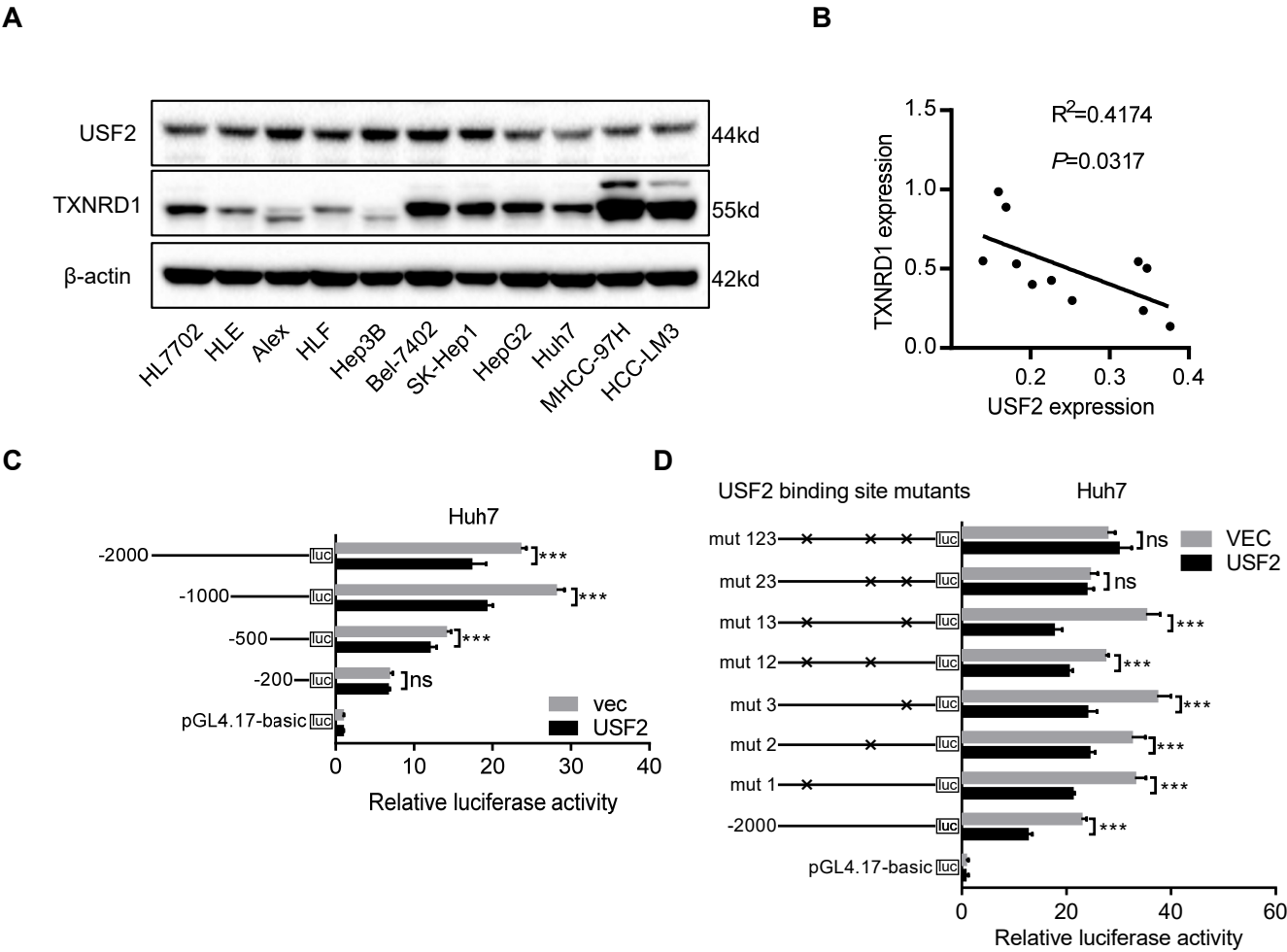

Supplement: Supplementary file 8 — Figure S5 [file 41419_2022_5363_MOESM8_ESM.pdf]

Figure S6

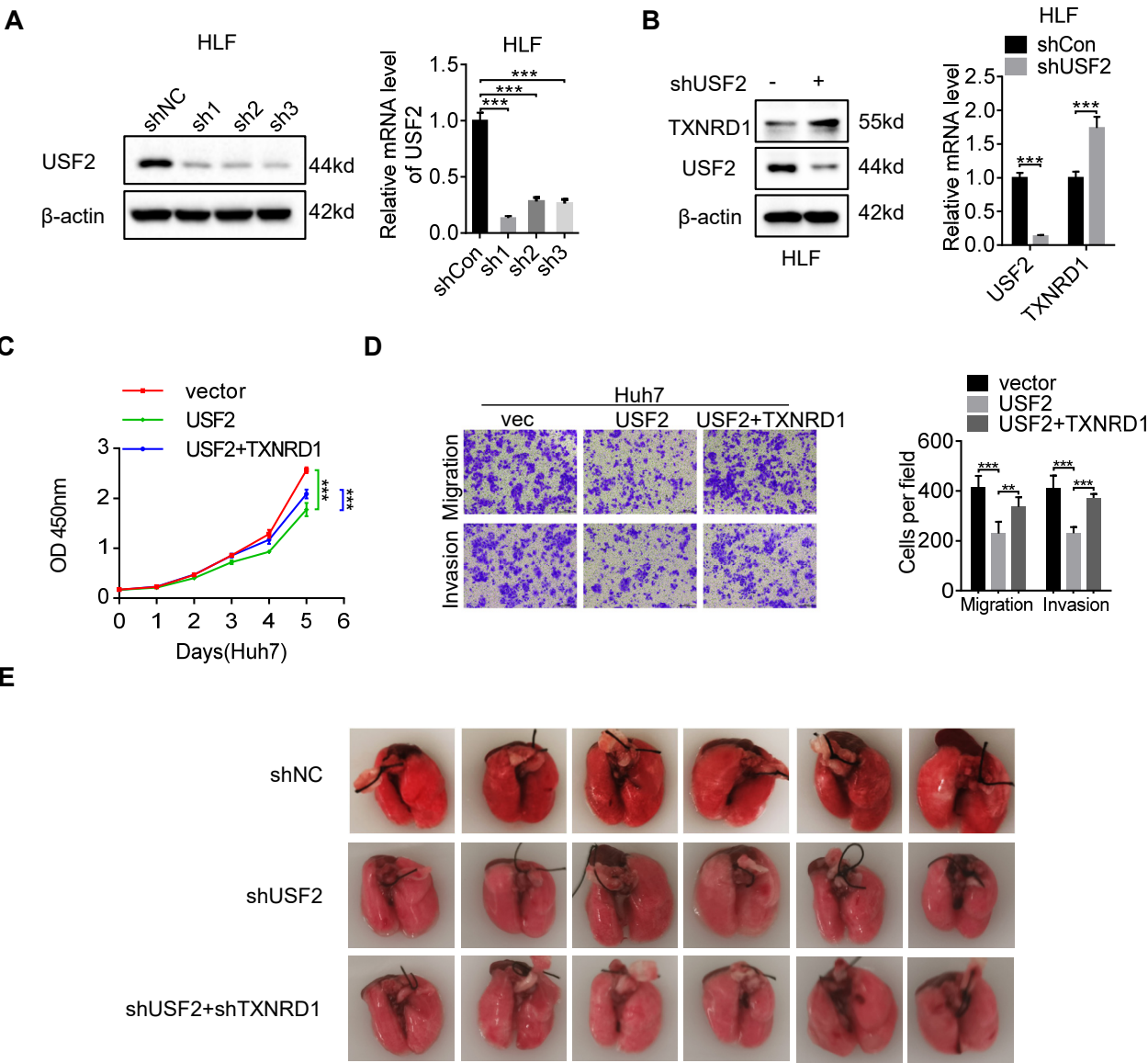

Supplement: Supplementary file 9 — Figure S6 [file 41419_2022_5363_MOESM9_ESM.pdf]

**Figure S7**

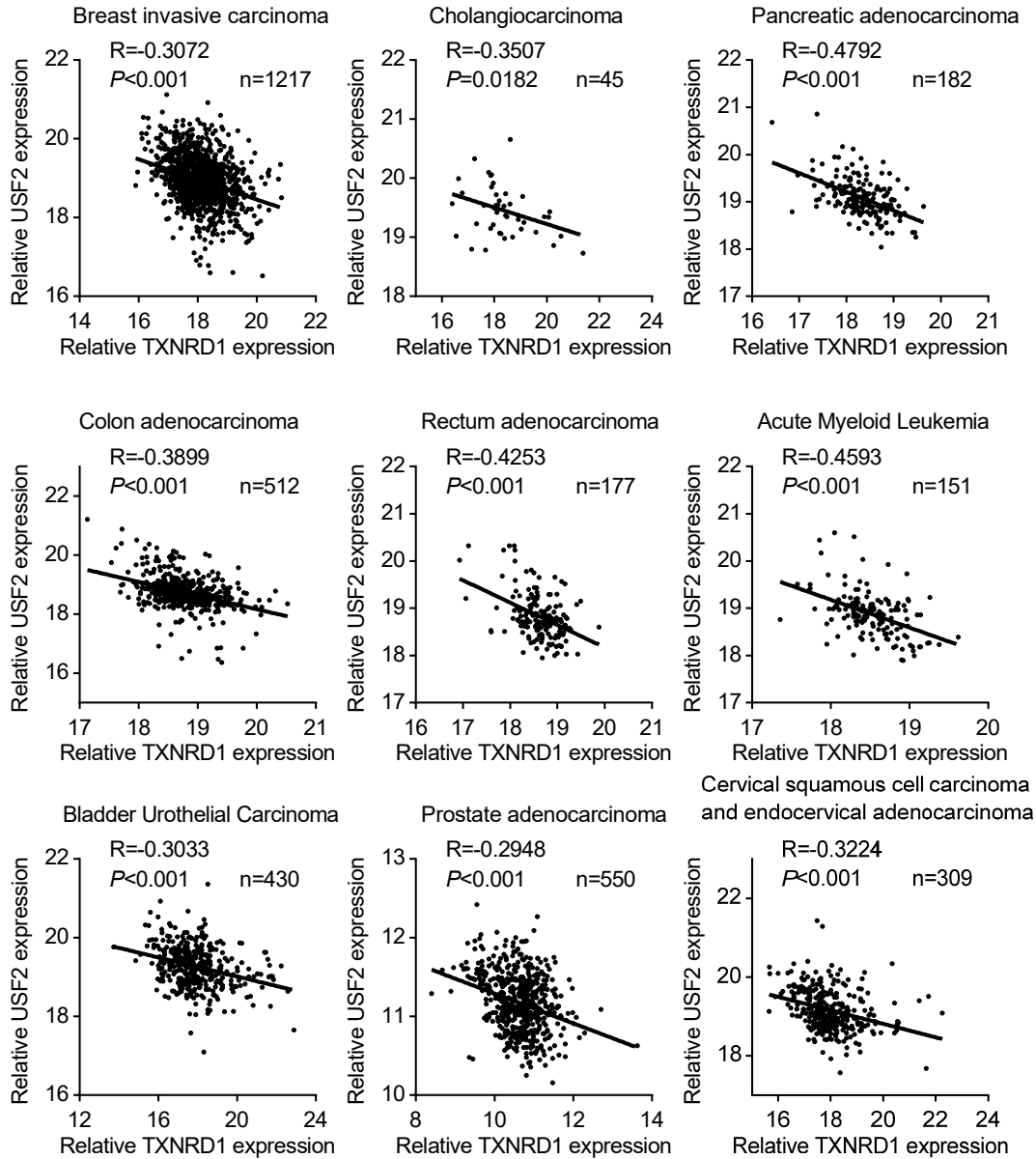

Supplement: Supplementary file 10 — Figure S7 [file 41419_2022_5363_MOESM10_ESM.pdf]
