# Supplementary material for: USF2-mediated upregulation of TXNRD1 contributes to hepatocellular carcinoma progression by activating Akt/mTOR signaling
Source: Cell Death Dis. 2022 Nov 1;13(11):917. doi: 10.1038/s41419-022-05363-x (PMC9626593; doi:10.1038/s41419-022-05363-x)
Supplement: Supplementary file 11 — Original Data File [file 41419_2022_5363_MOESM11_ESM.pdf]

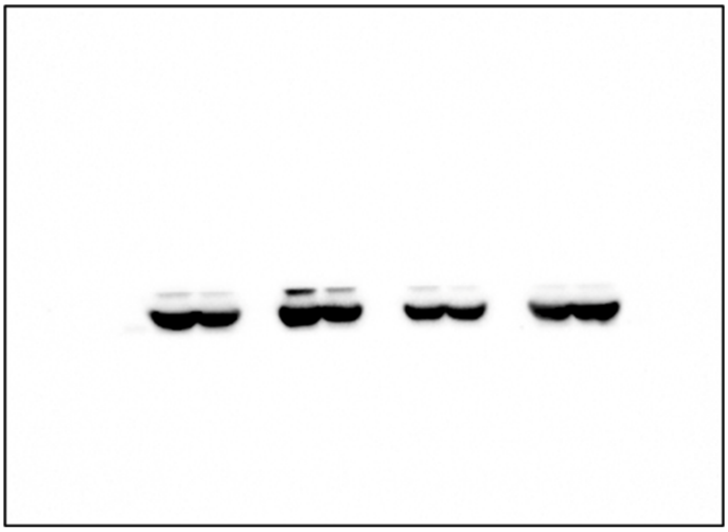

Figure 1C actin T1-N4

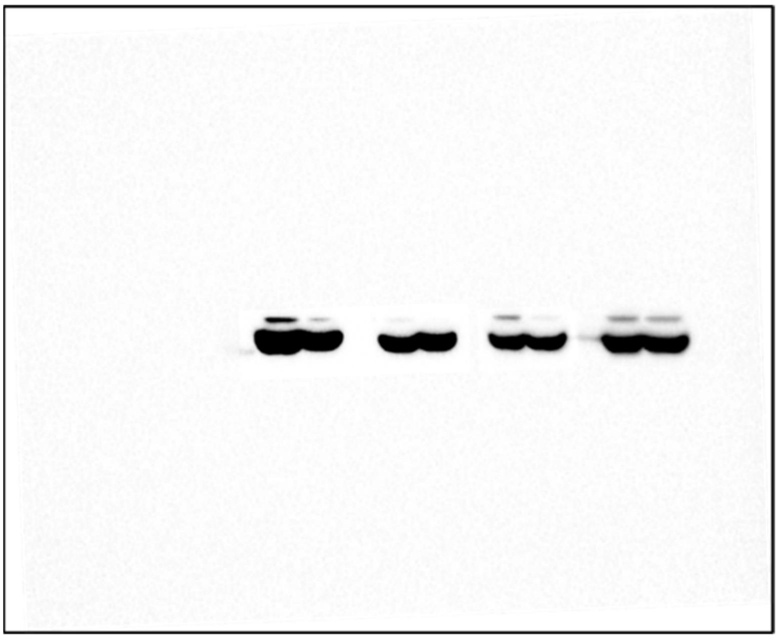

Figure 1C actin T5-N8

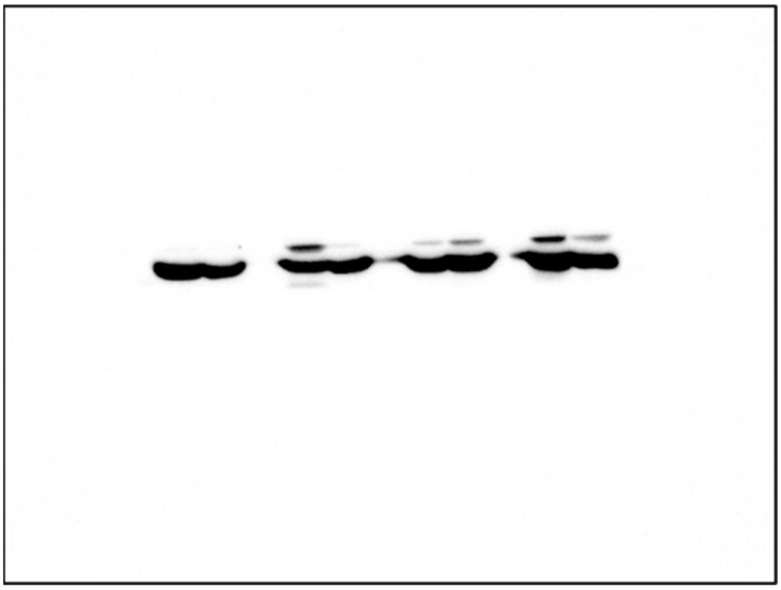

Figure 1C actin T9-N12

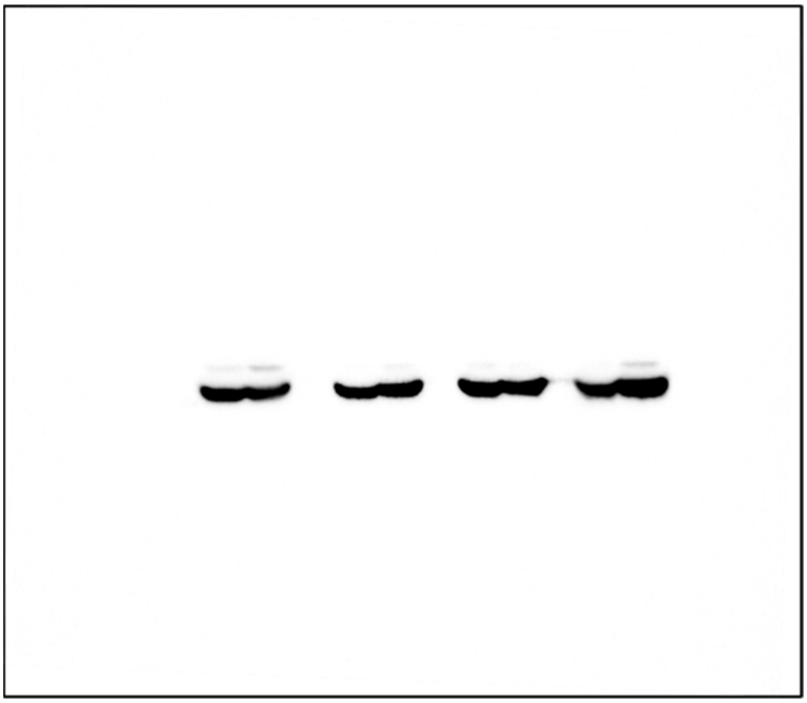

Figure 1C actin T13-N16

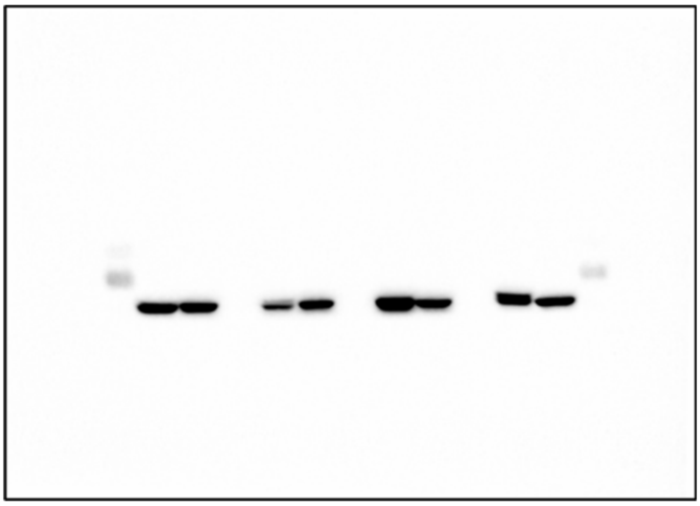

Figure 1C TXNRD1 T1-N4

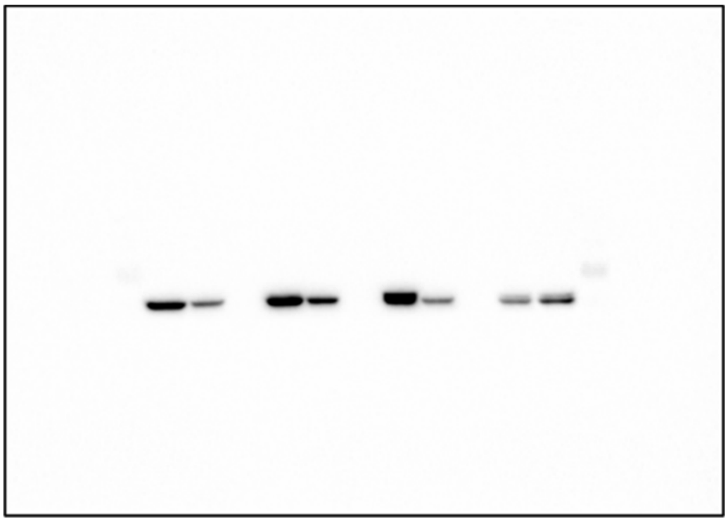

Figure 1C TXNRD1 T5-N8

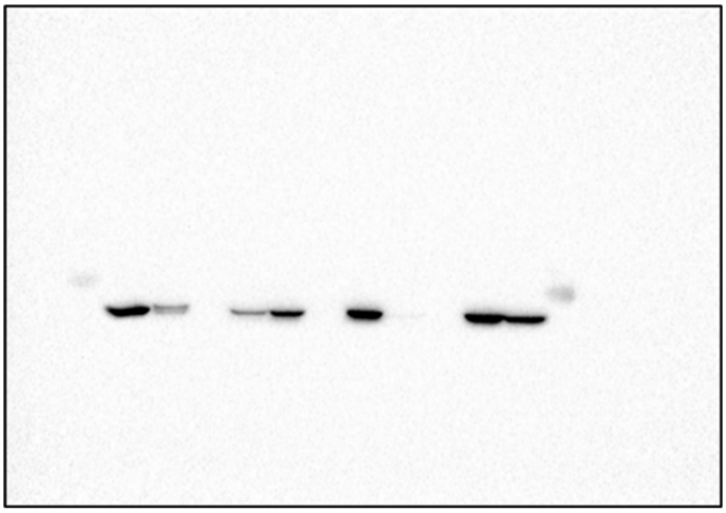

Figure 1C TXNRD1 T9-N12

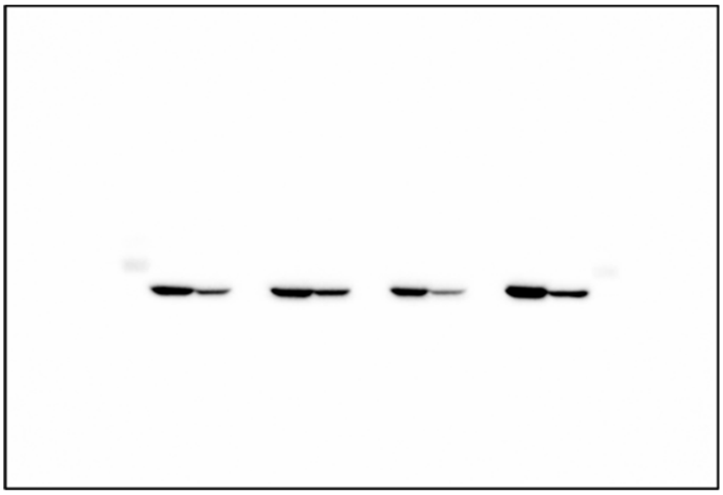

Figure 1C TXNRD1 T13-N16

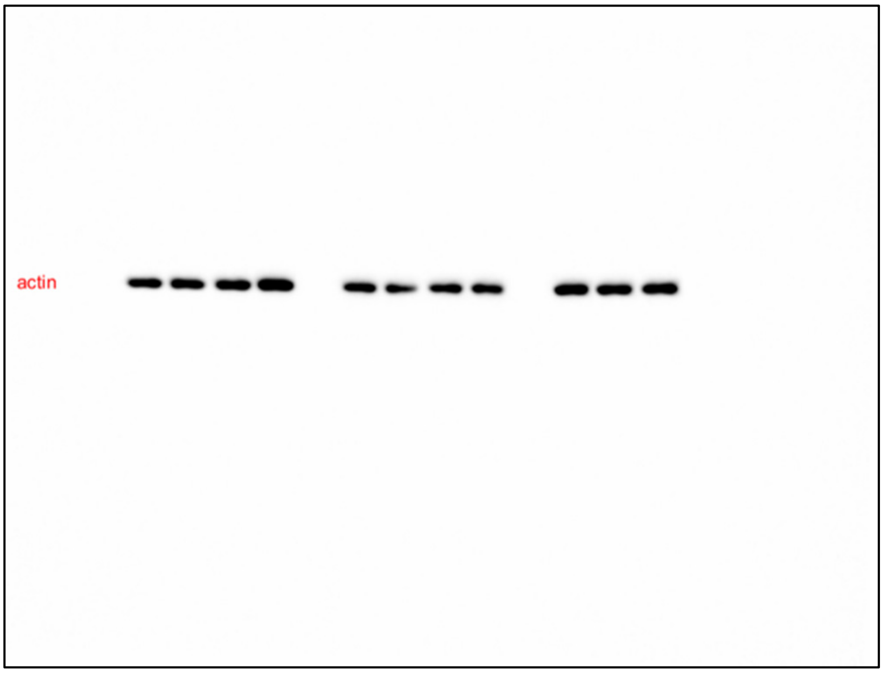

Figure 4A 97H actin

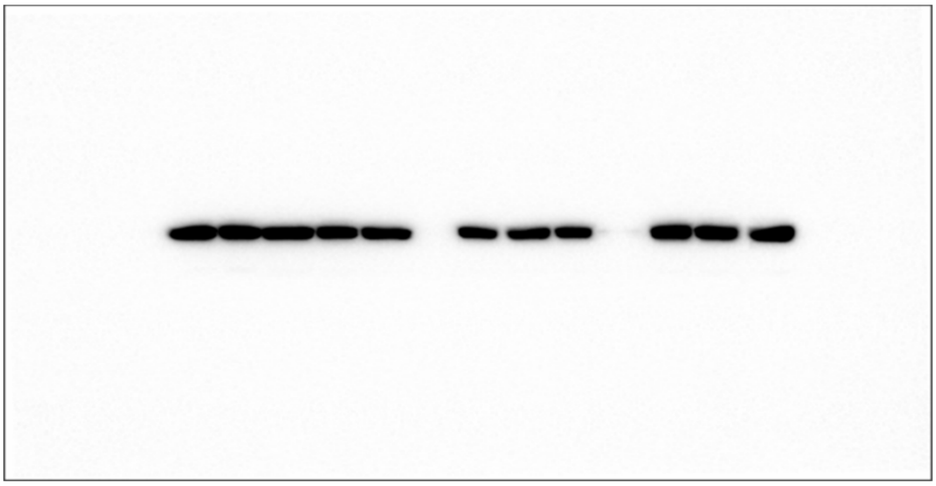

Figure 4A 97H Akt

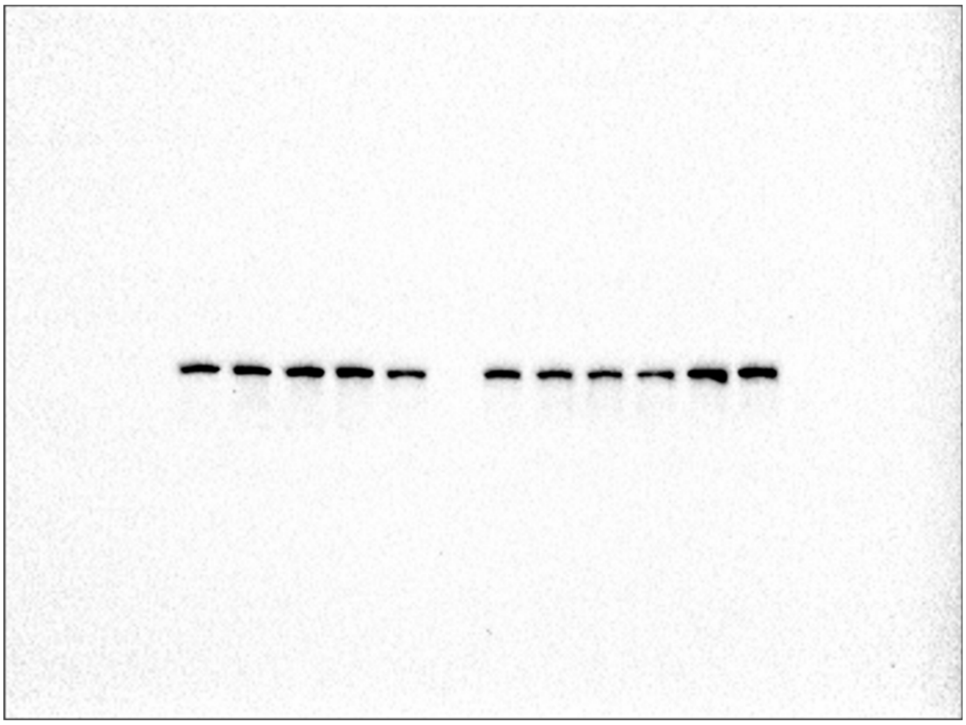

Figure 4A 97H MTOR

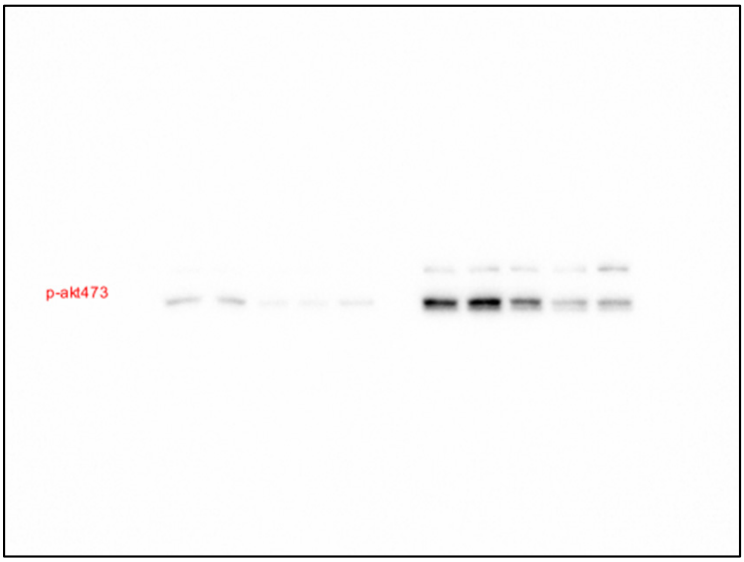

Figure 4A 97H p-Akt

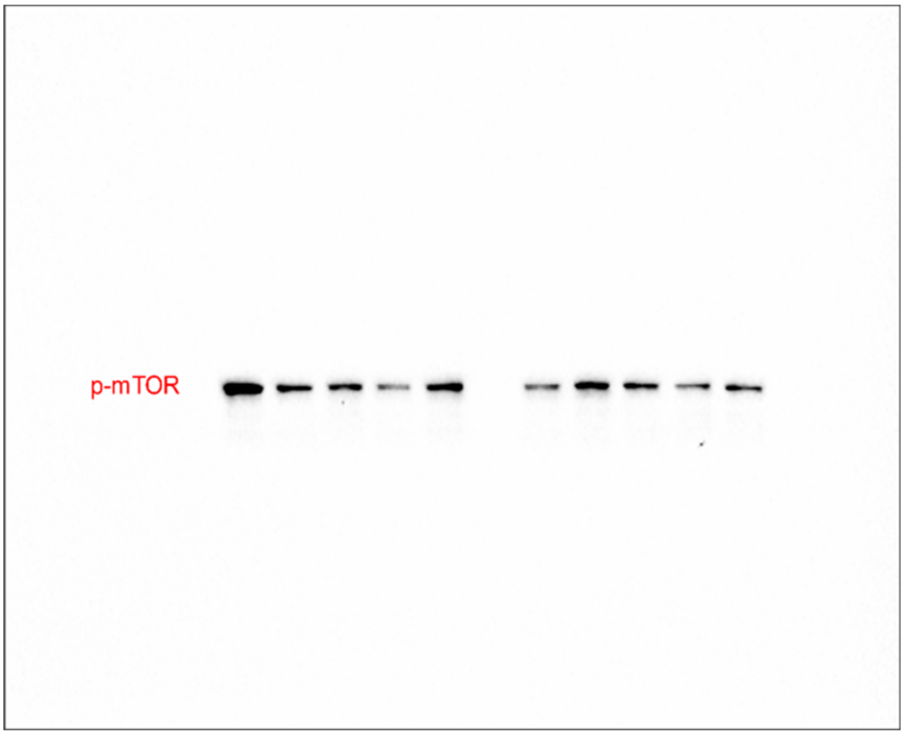

Figure 4A 97H p-MTOR

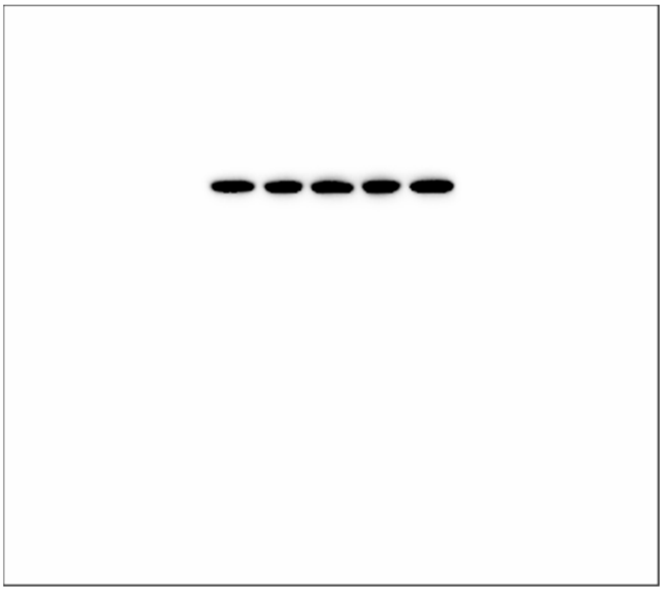

Figure 4A 7402 actin

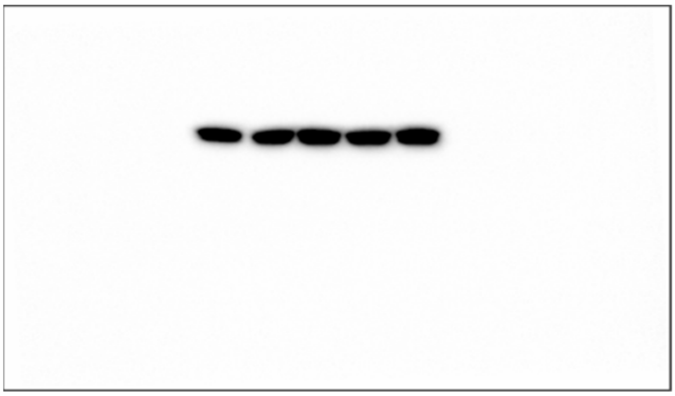

Figure 4A 7402 Akt

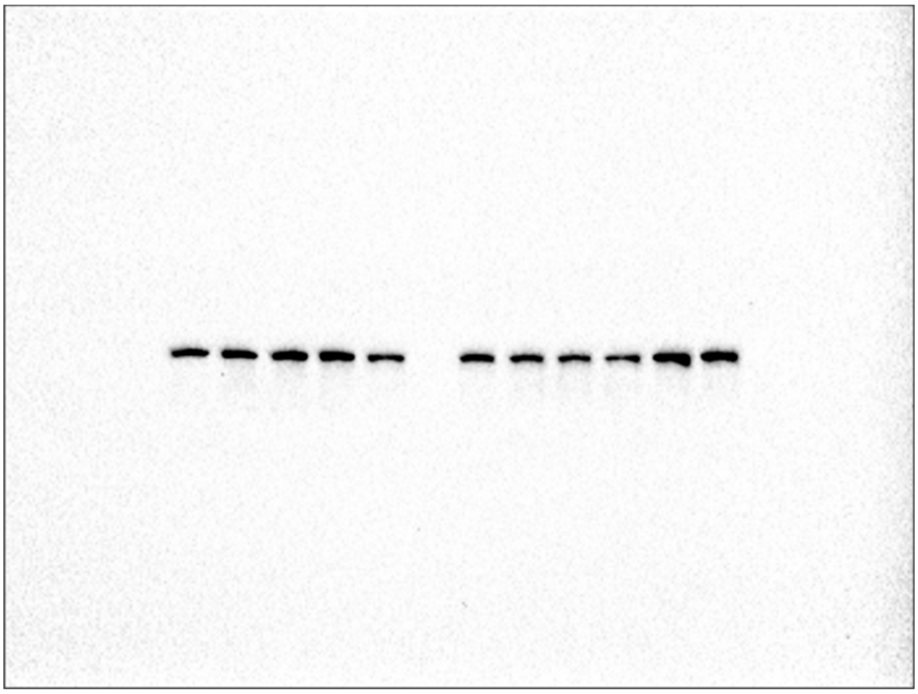

Figure 4A 7402 mTOR

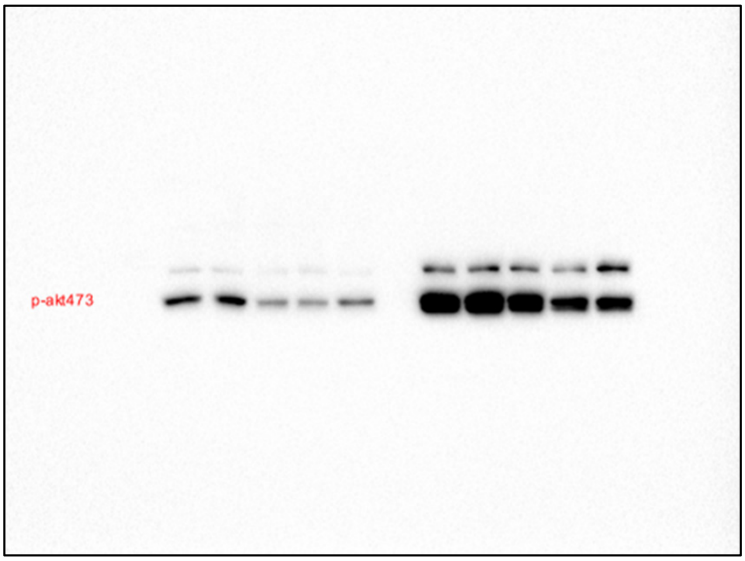

Figure 4A 7402 p-Akt

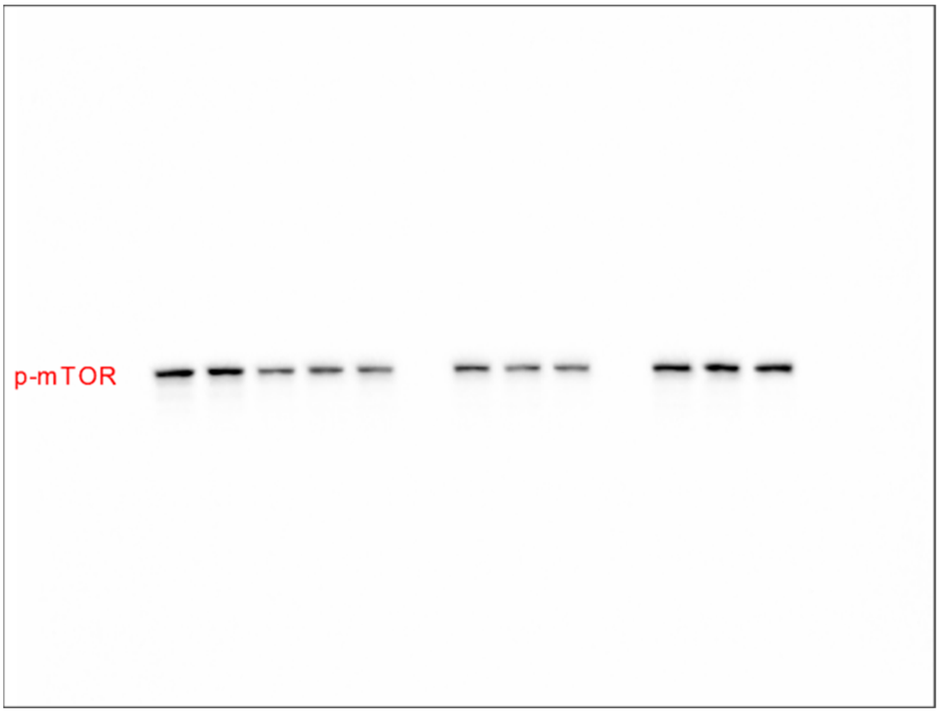

Figure 4A 7402 p-MTOR

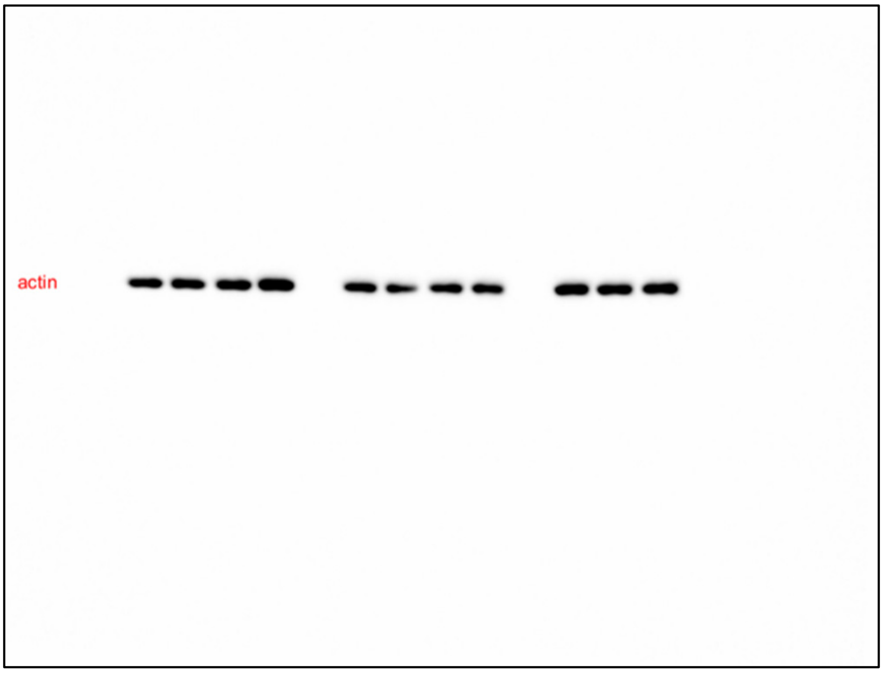

Figure 4A Alex actin

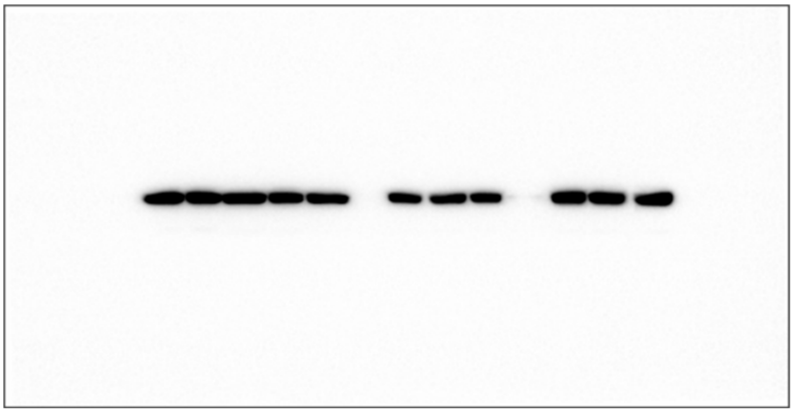

Figure 4A Alex Akt

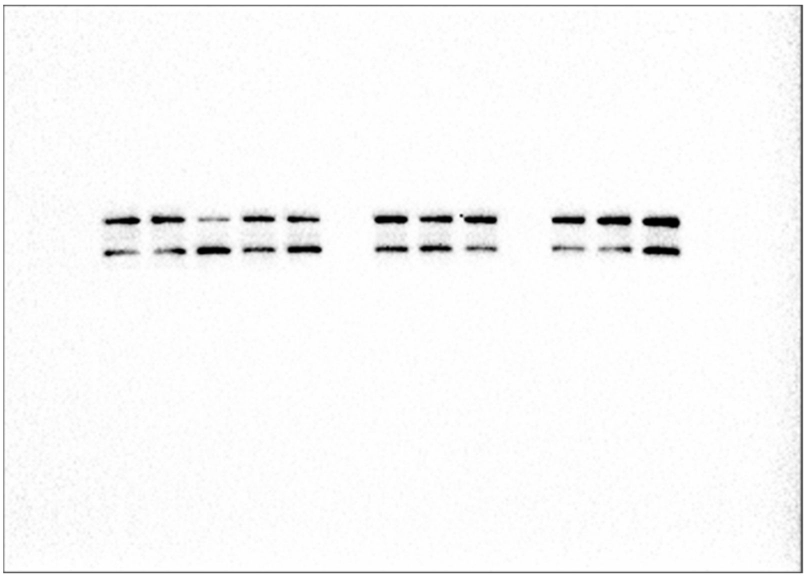

Figure 4A Alex MTOR

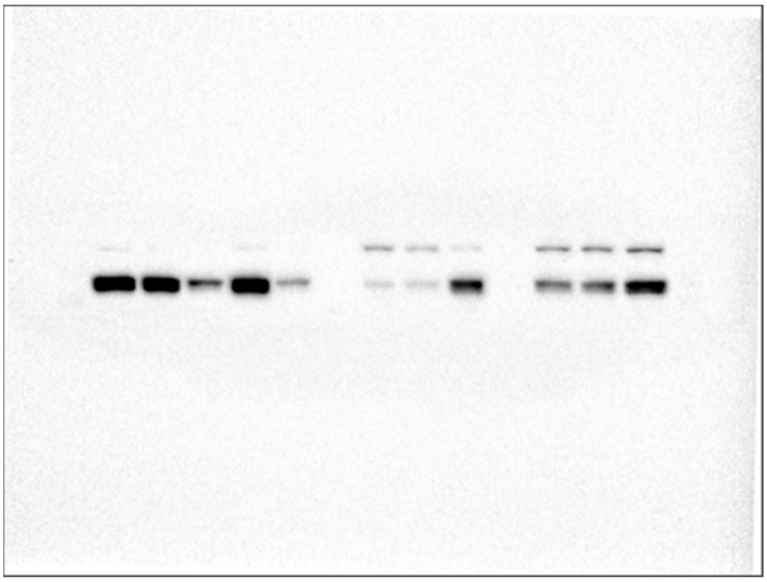

Figure 4A Alex p-Akt

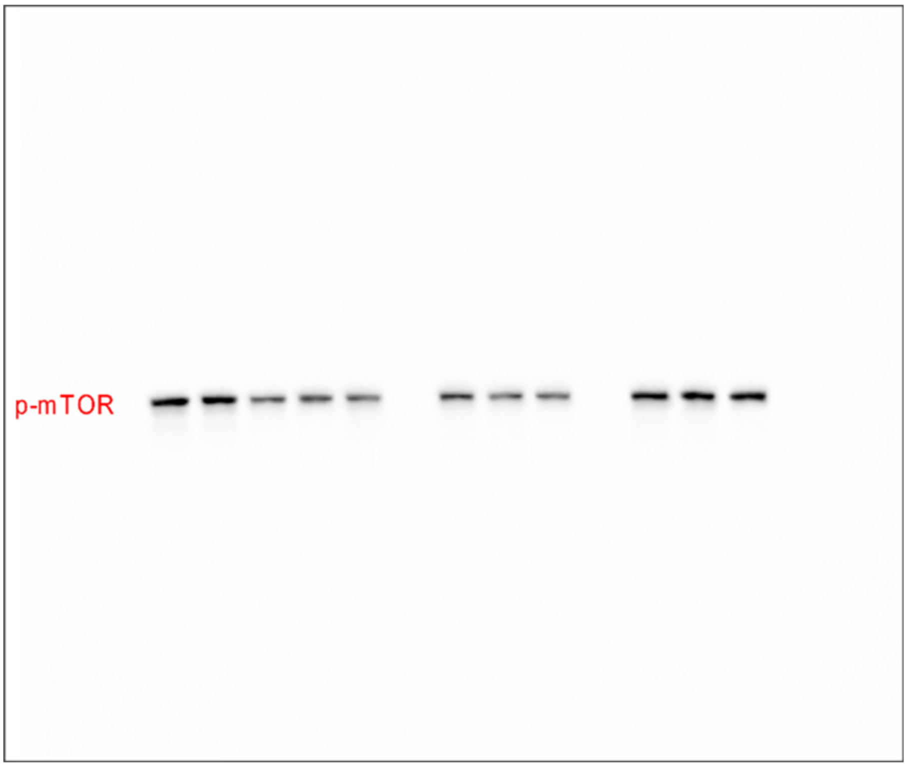

Figure 4A Alex p-MTOR

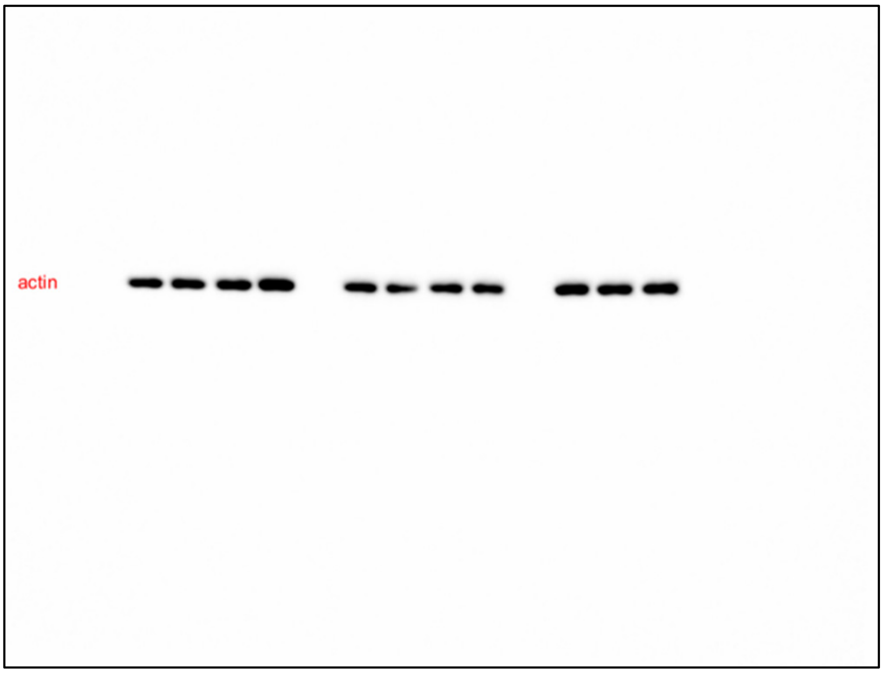

Figure 4A HLF actin

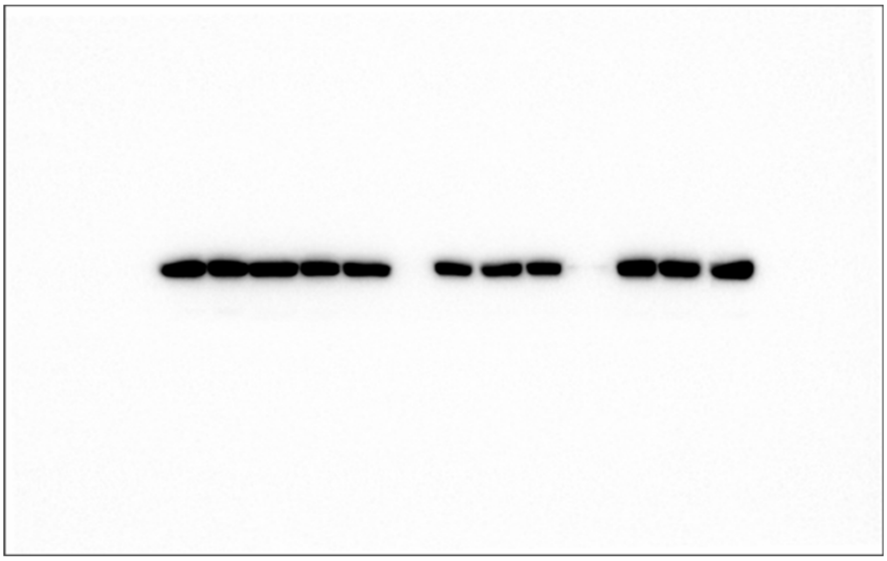

Figure 4A HLF Akt

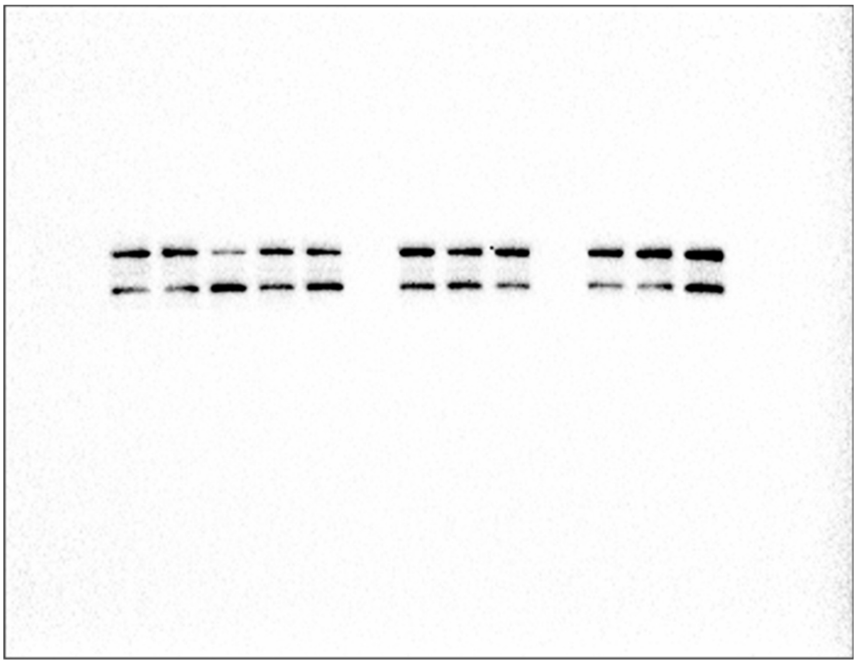

Figure 4A HLF Akt

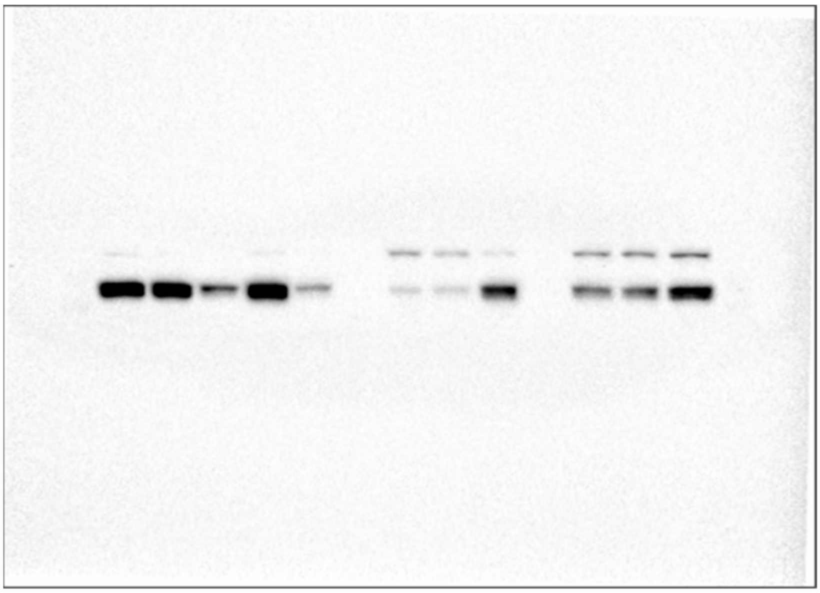

Figure 4A HLF p-Akt

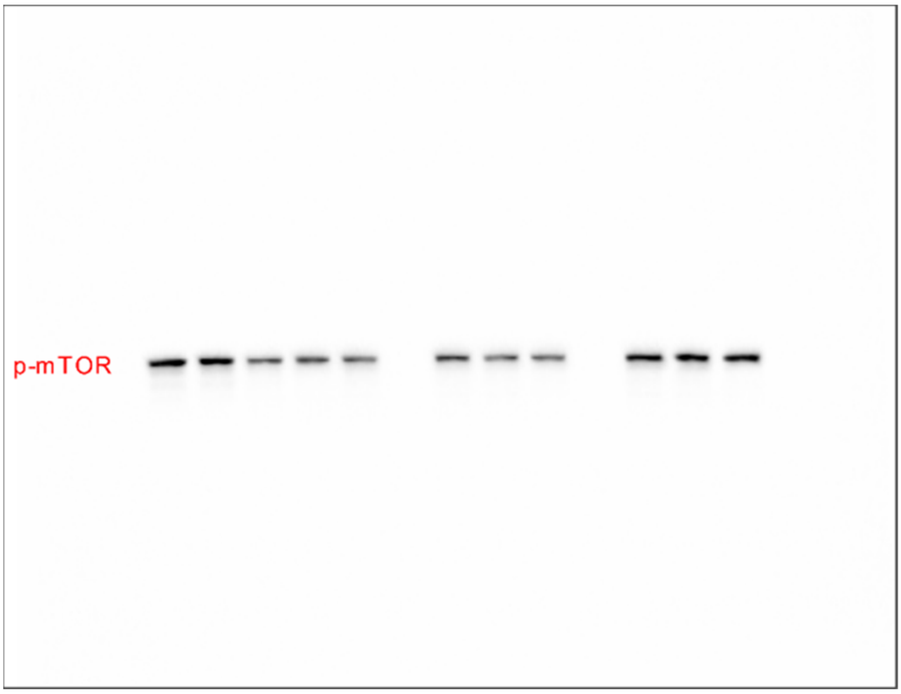

Figure 4A HLF p-MTOR

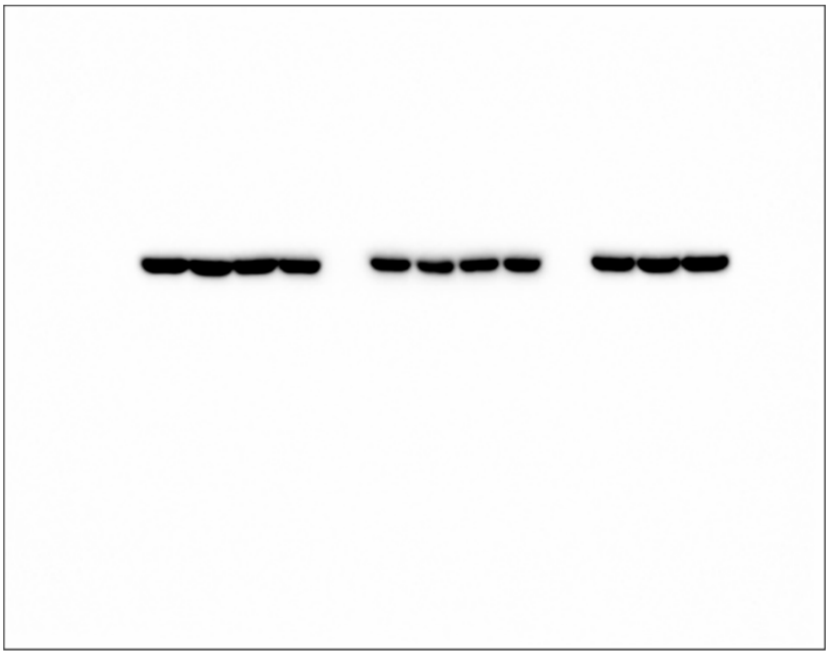

Figure 4B 97H actin

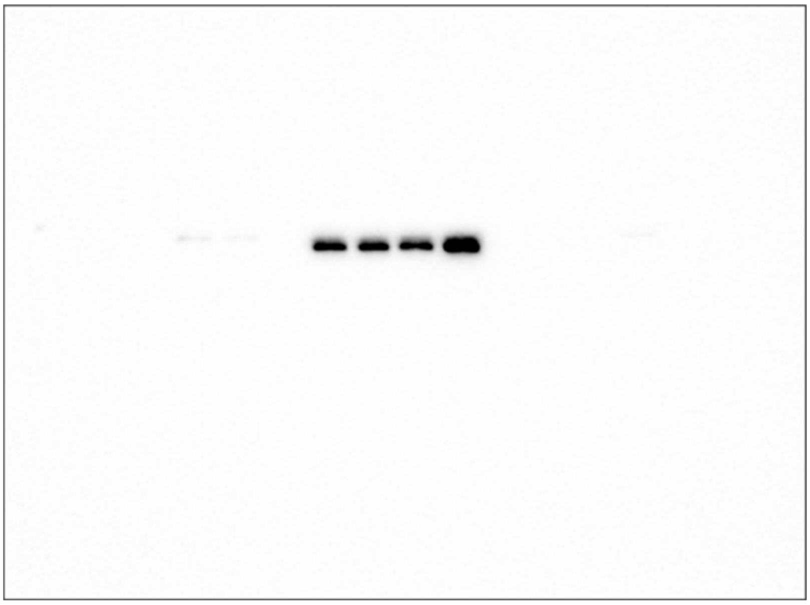

Figure 4B 97H E-cad

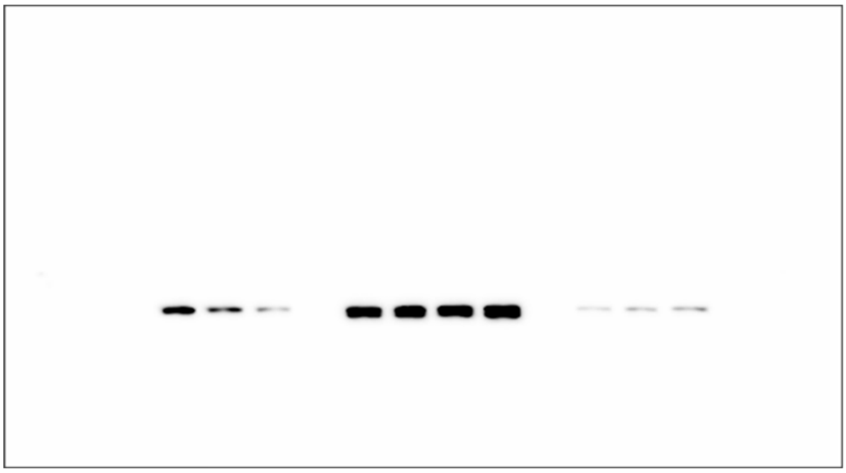

Figure 4B 97H N-cad

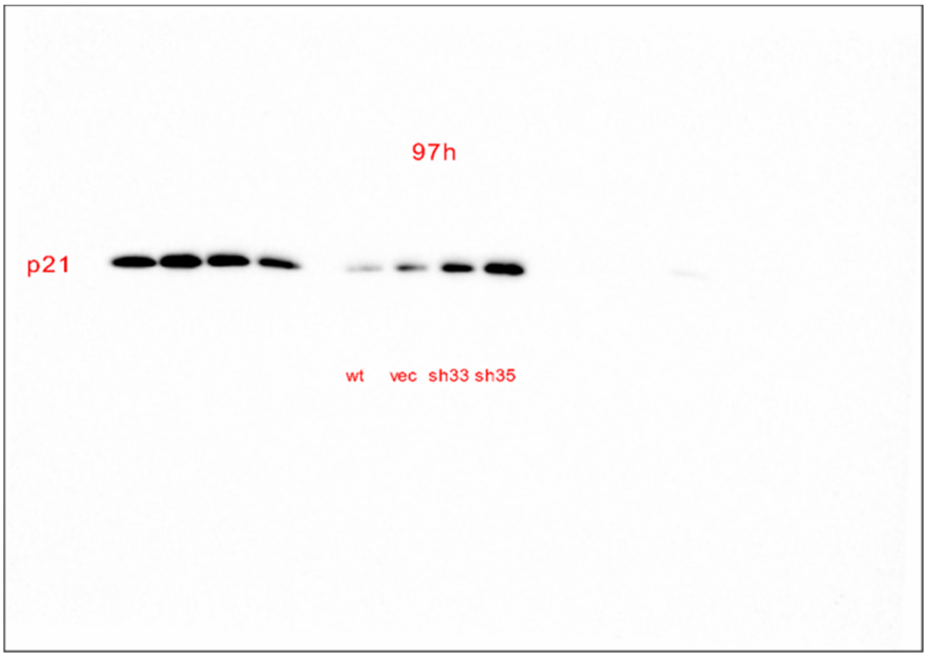

Figure 4B 97H p21

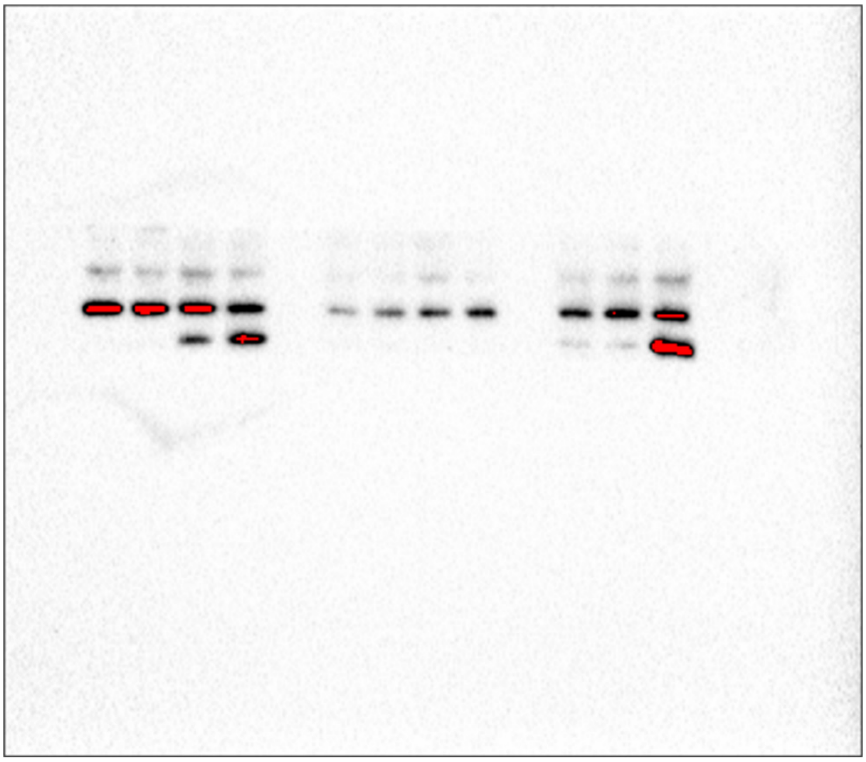

Figure 4B 97H Snail

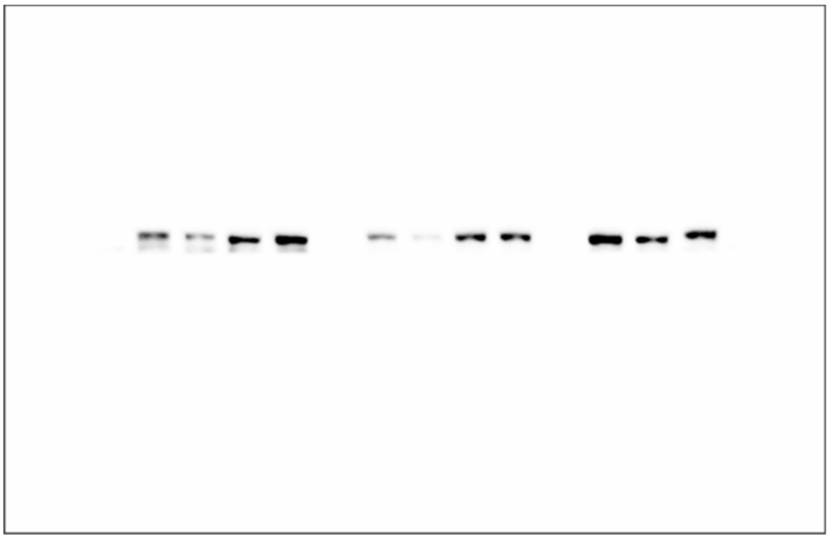

Figure 4B 97H ZO1

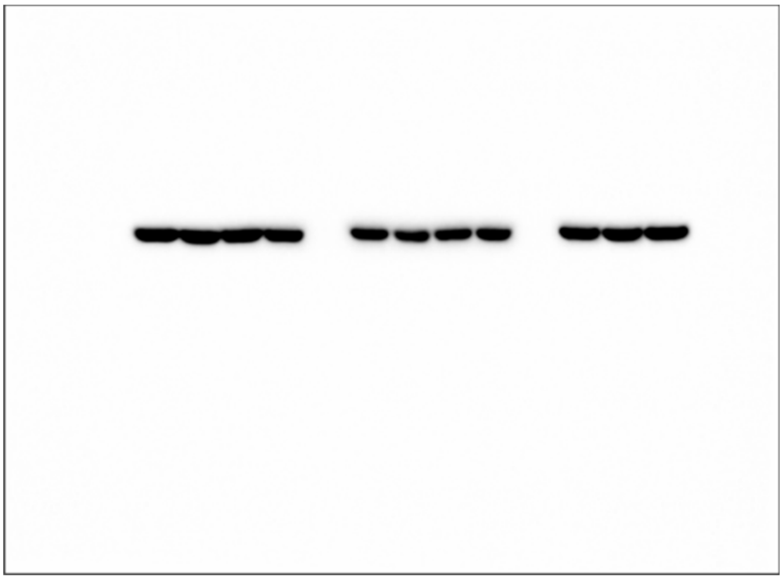

Figure 4B 7402 actin

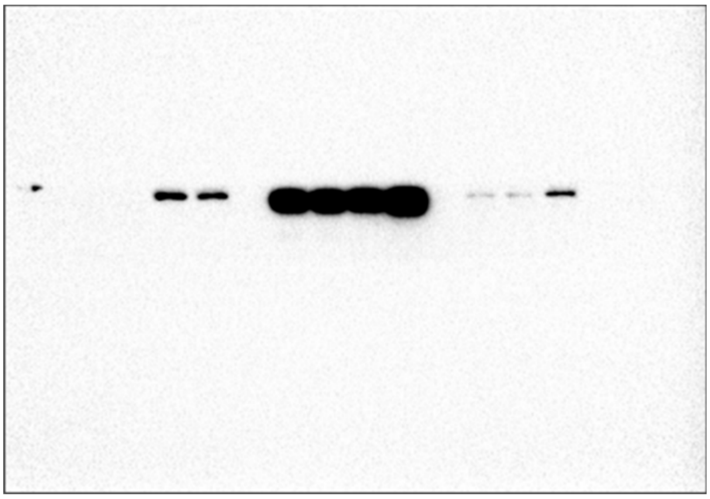

Figure 4B 7402 E-cad

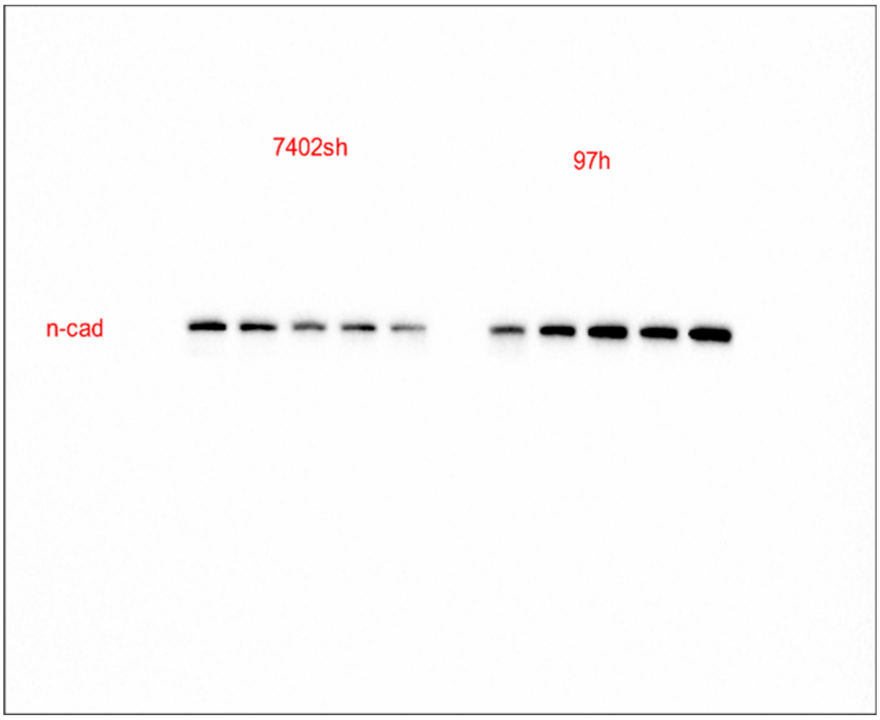

Figure 4B 7402 N-cad

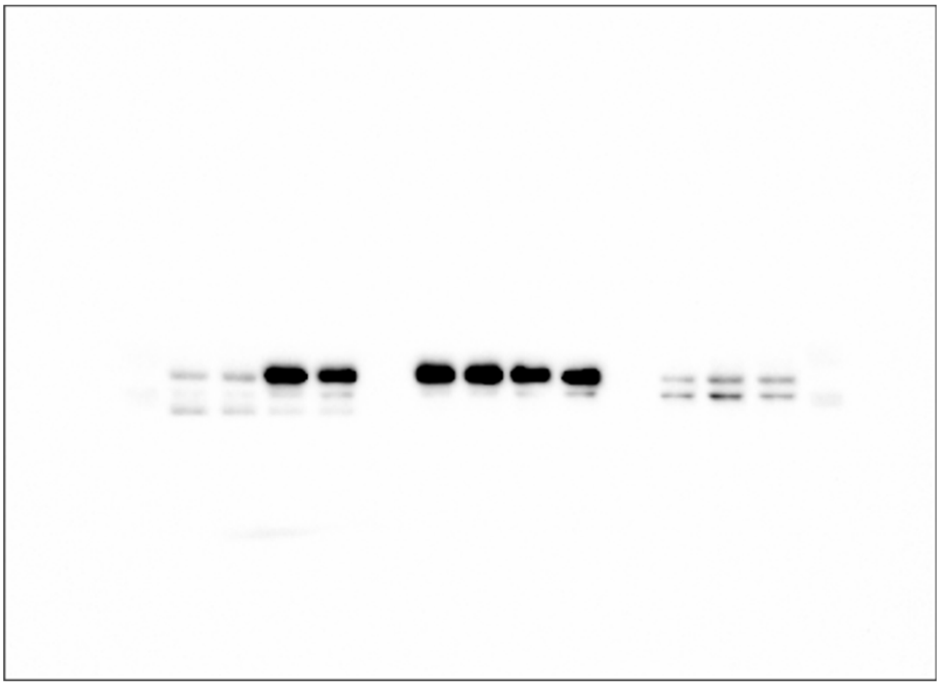

Figure 4B 7402 Occludin

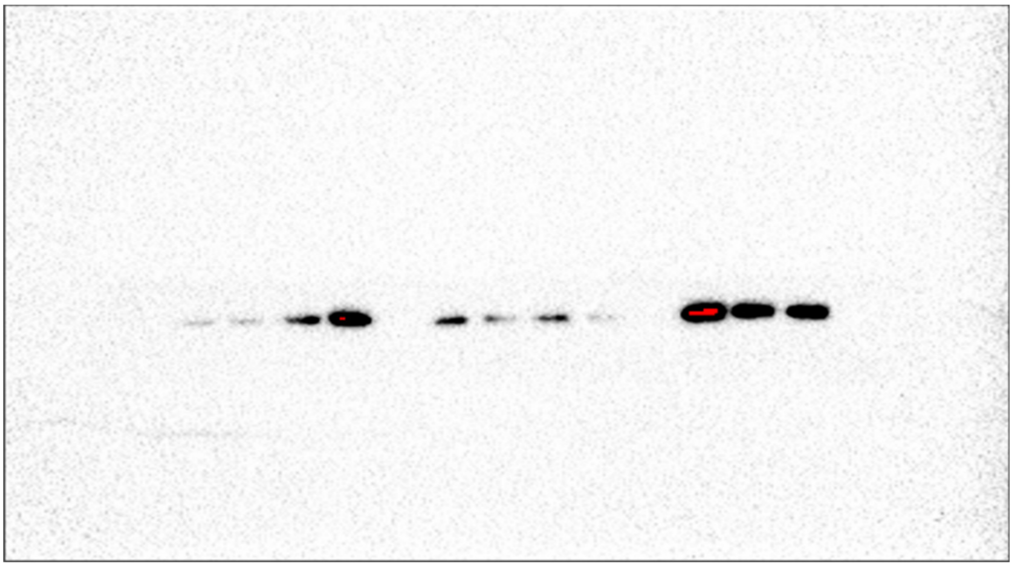

Figure 4B 7402 p21

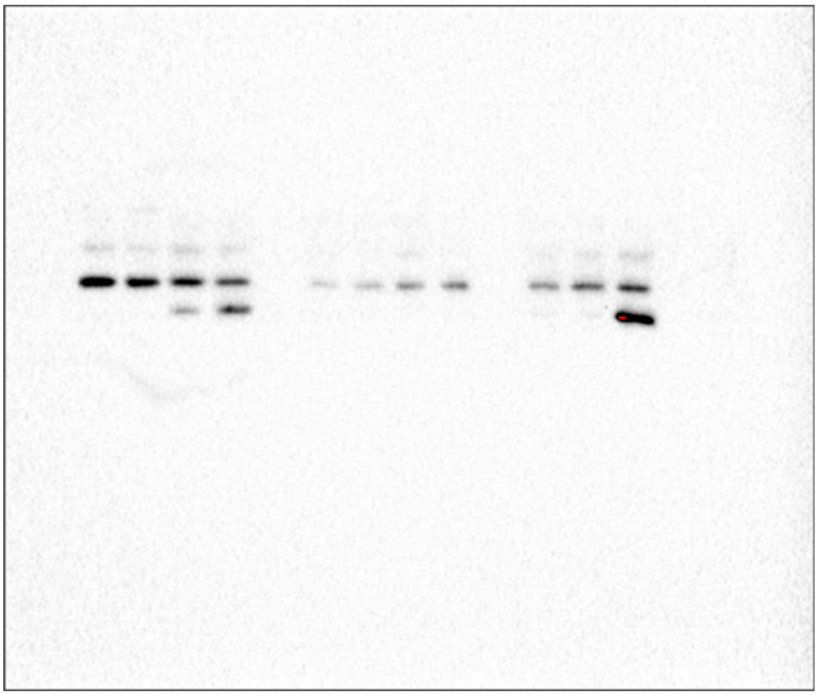

Figure 4B 7402 Snail

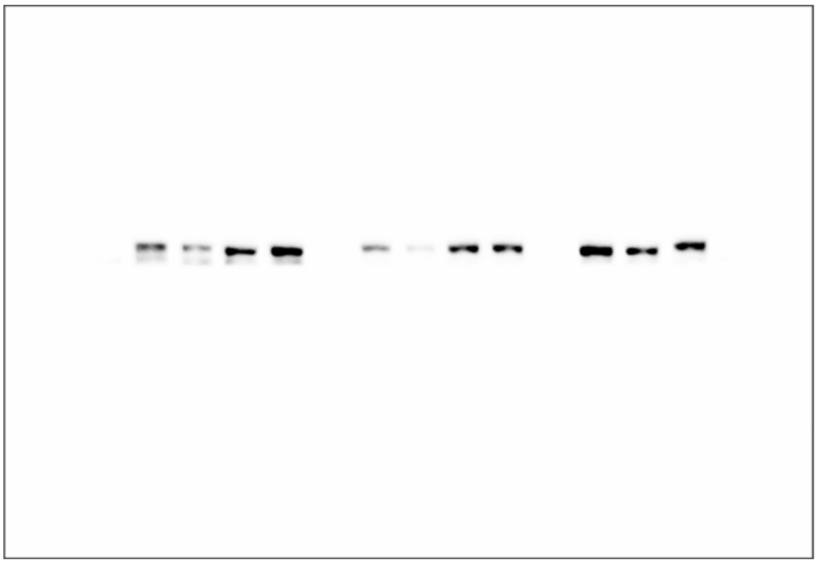

Figure 4B 7402 ZO1

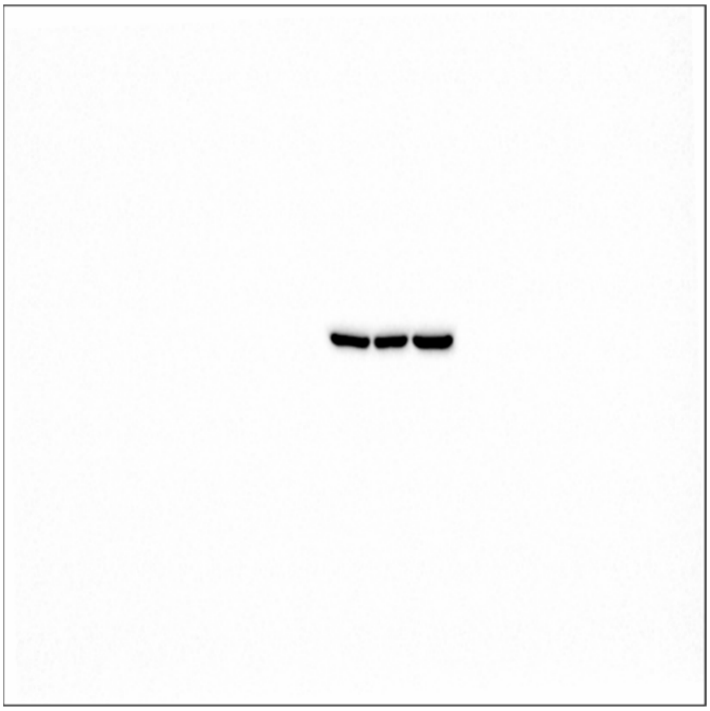

Figure 4B Alex actin

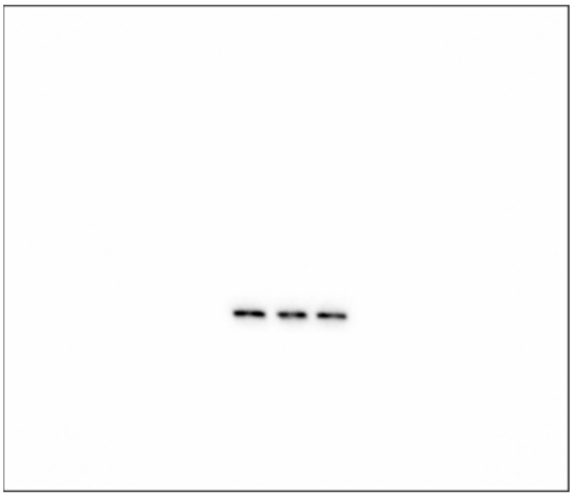

Figure 4B Alex E-cad

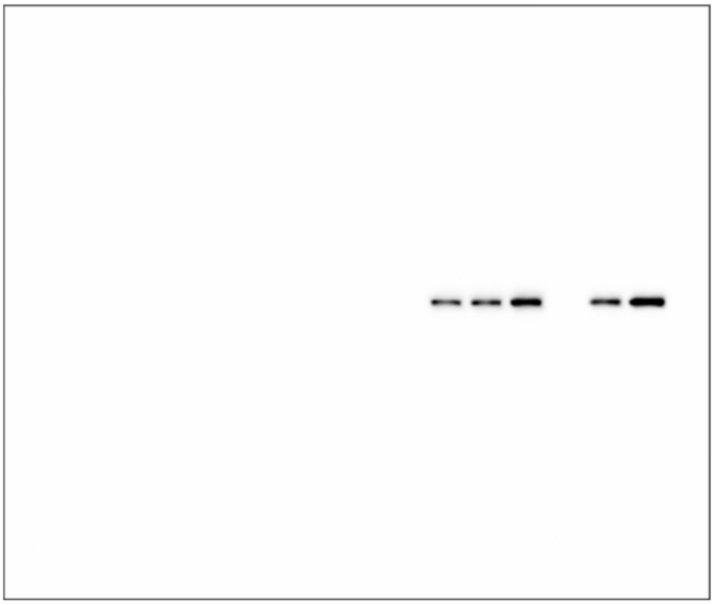

Figure 4B Alex N-cad

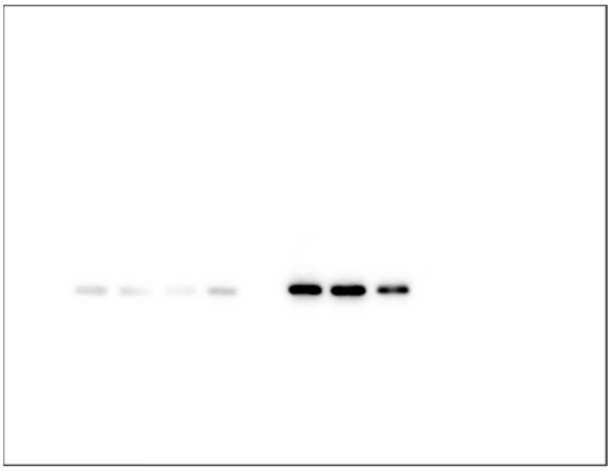

Figure 4B Alex Occludin

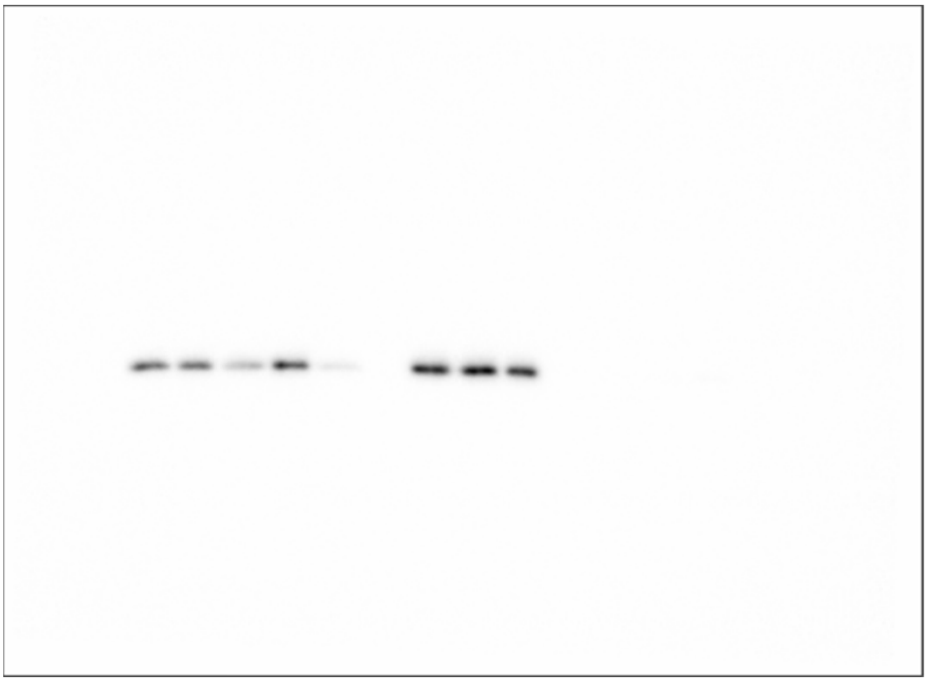

Figure 4B Alex p21

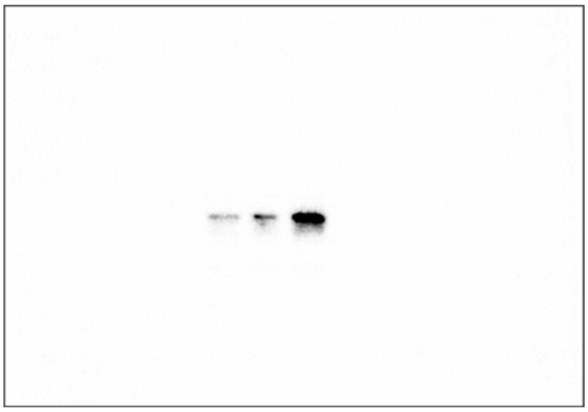

Figure 4B Alex Snail

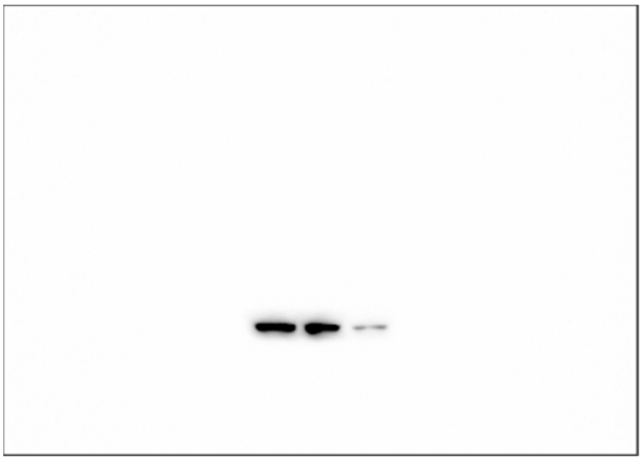

Figure 4B Alex ZO1

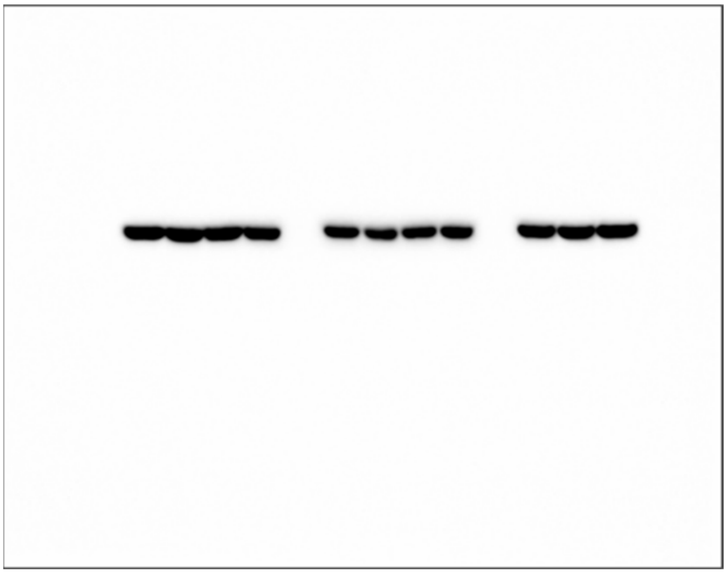

Figure 4B HLF actin

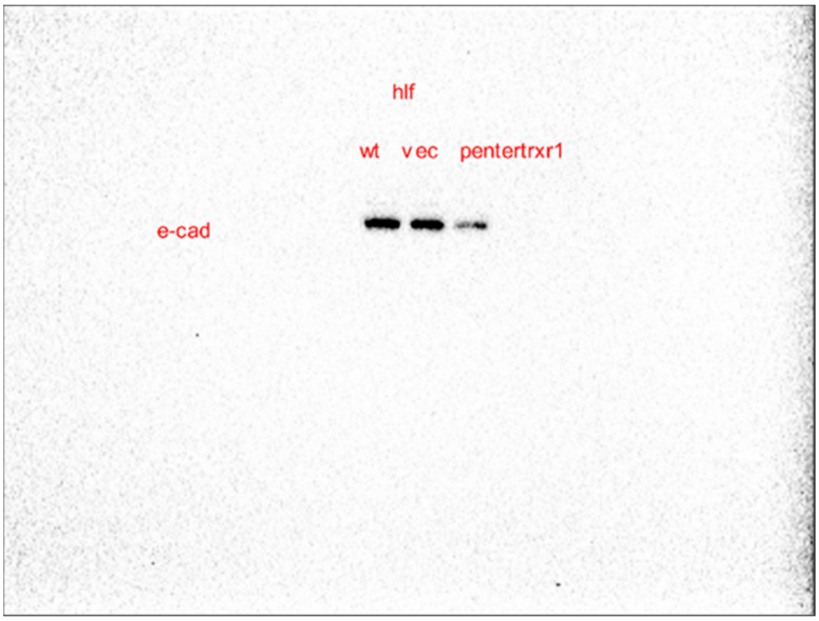

Figure 4B HLF E-cad

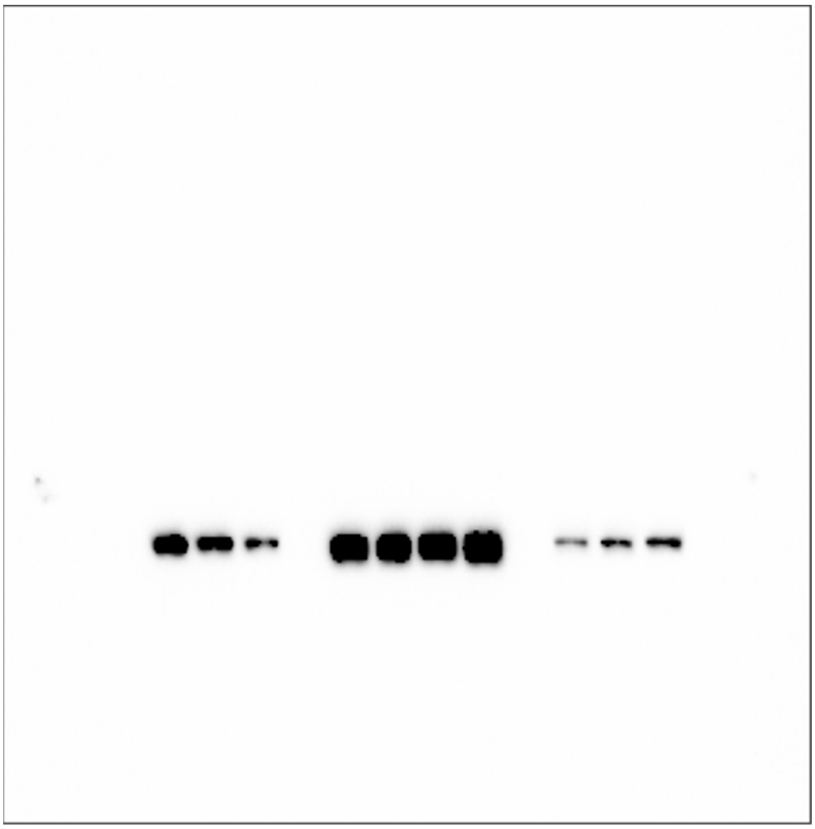

Figure 4B HLF N-cad

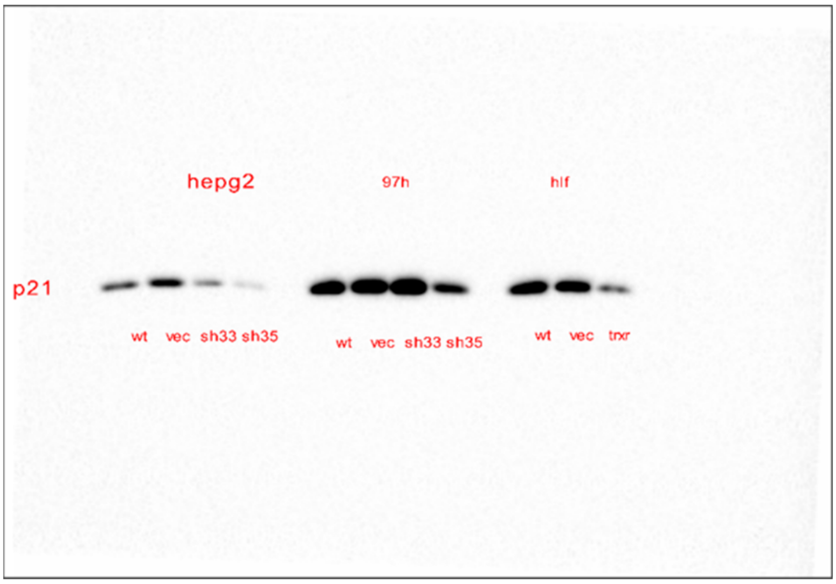

Figure 4B HLF p21

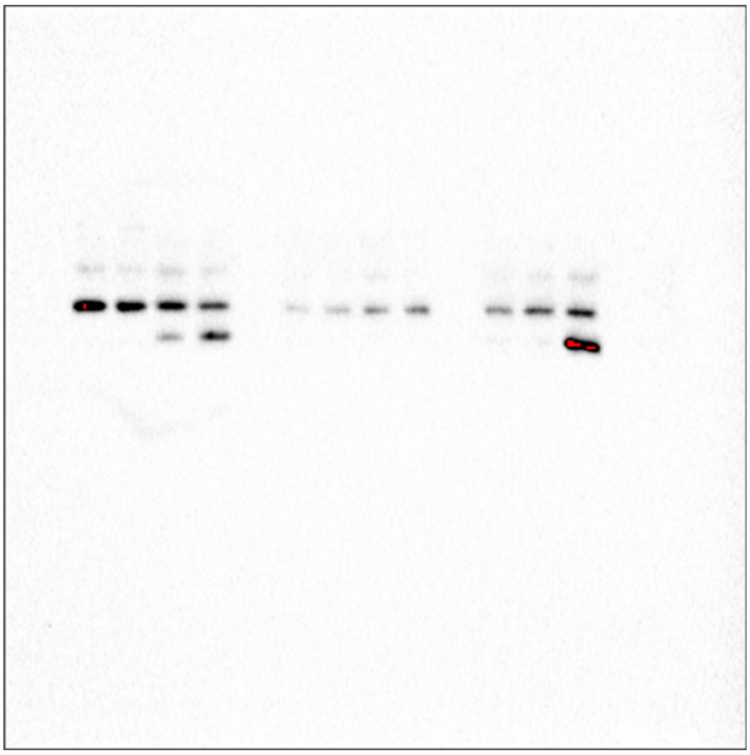

Figure 4B HLF Snail

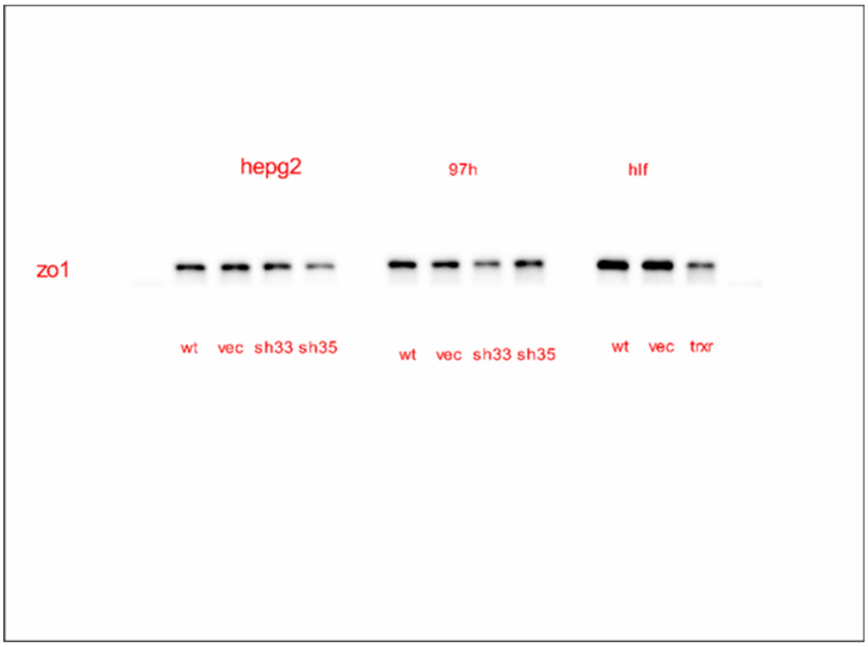

Figure 4B HLF ZO1

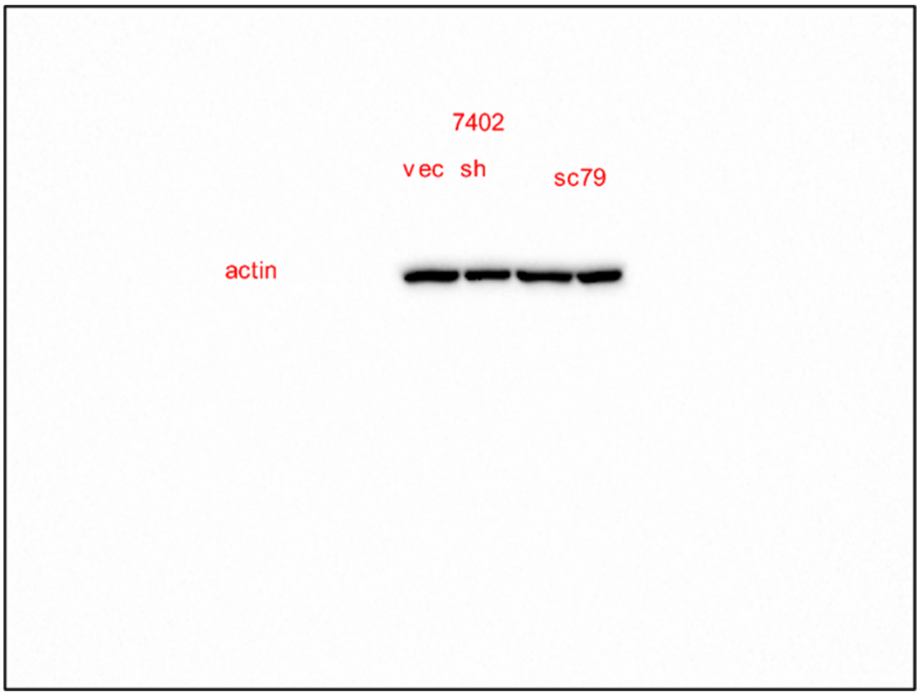

Figure 4F 7402 actin

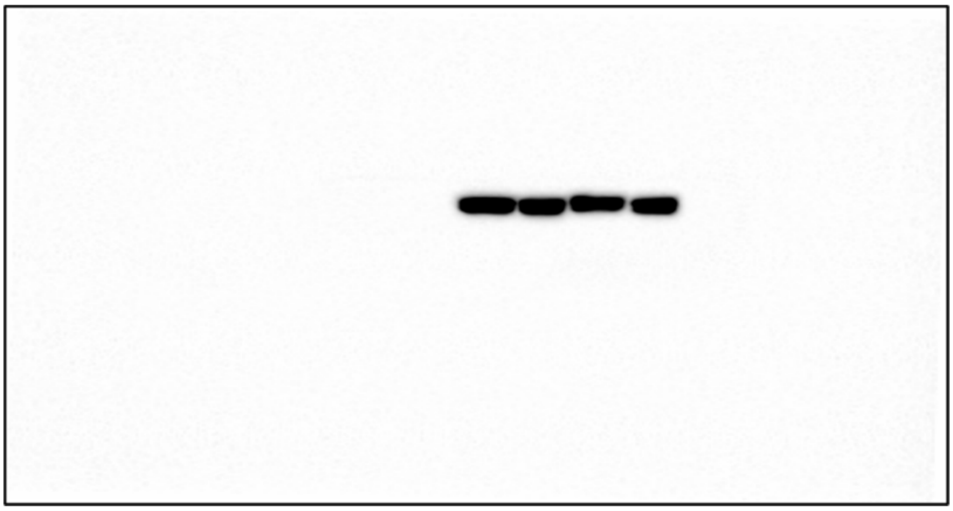

Figure 4F 7402 Akt

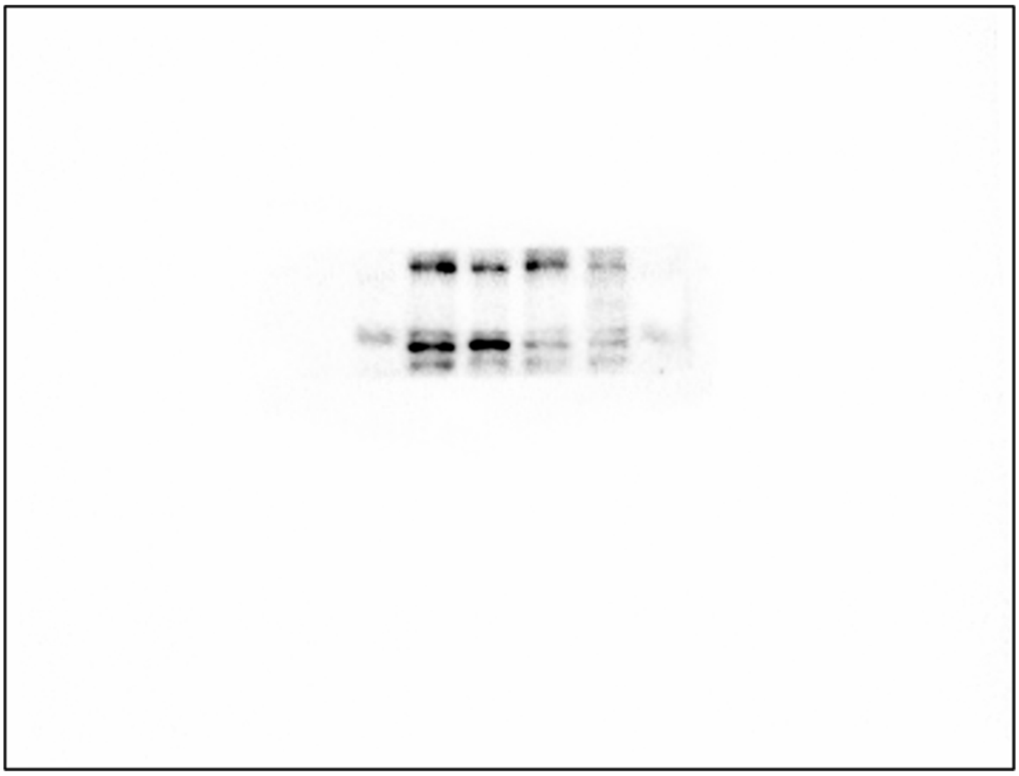

Figure 4F 7402 E-cad

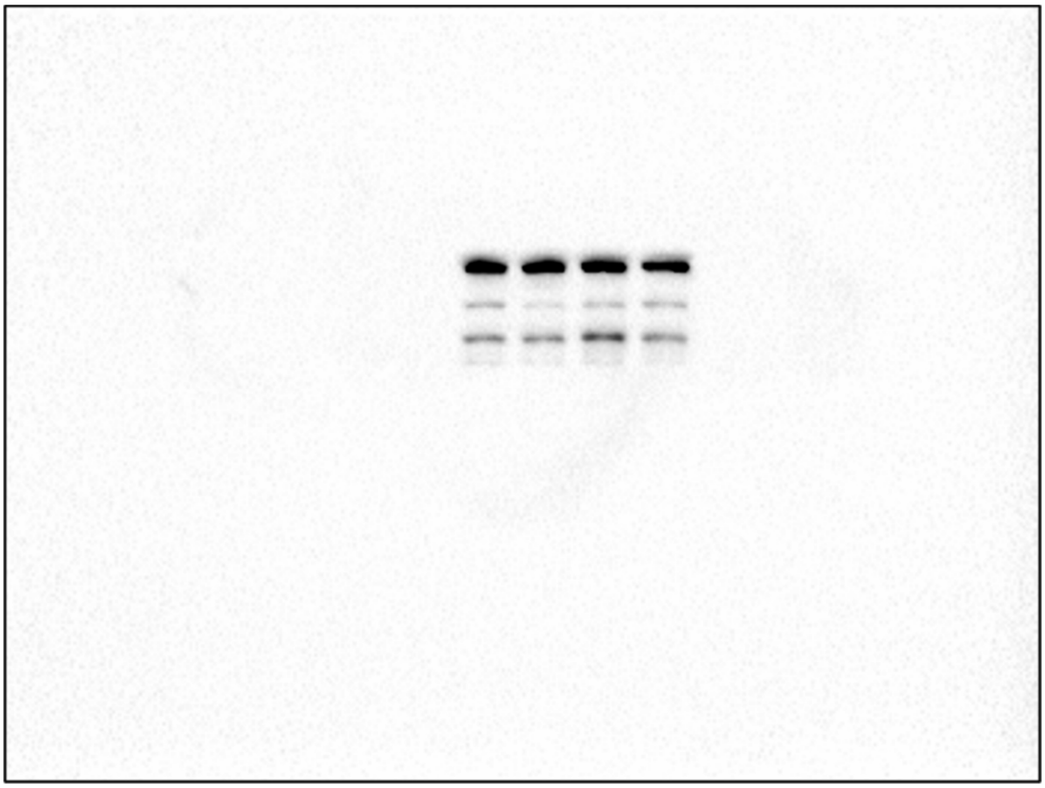

Figure 4F 7402 MTOR

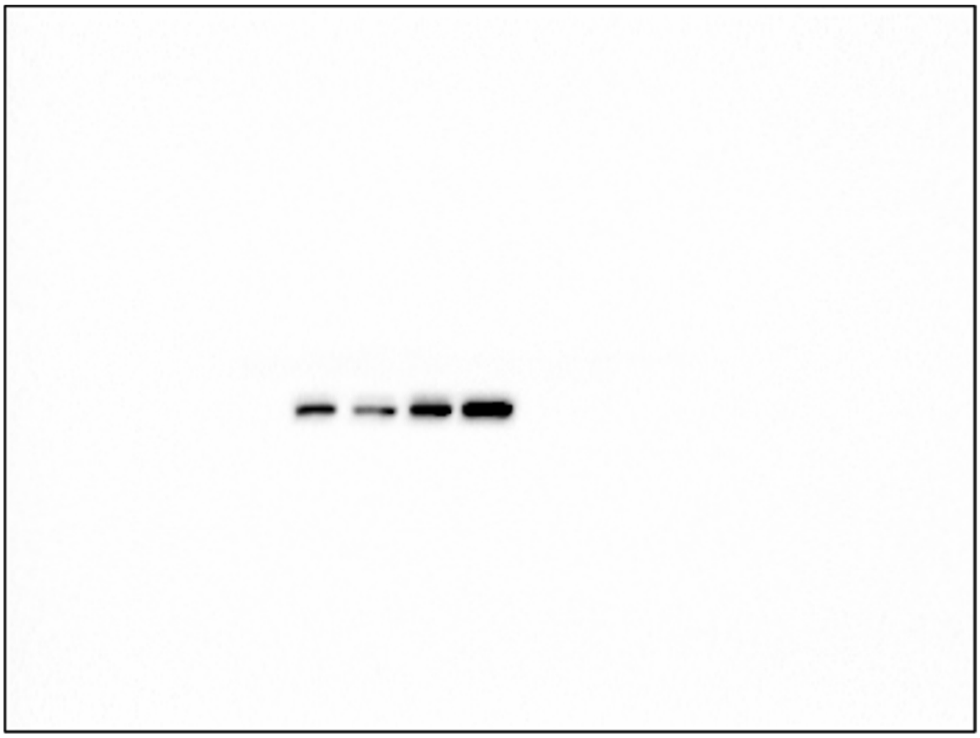

Figure 4F 7402 N-cad

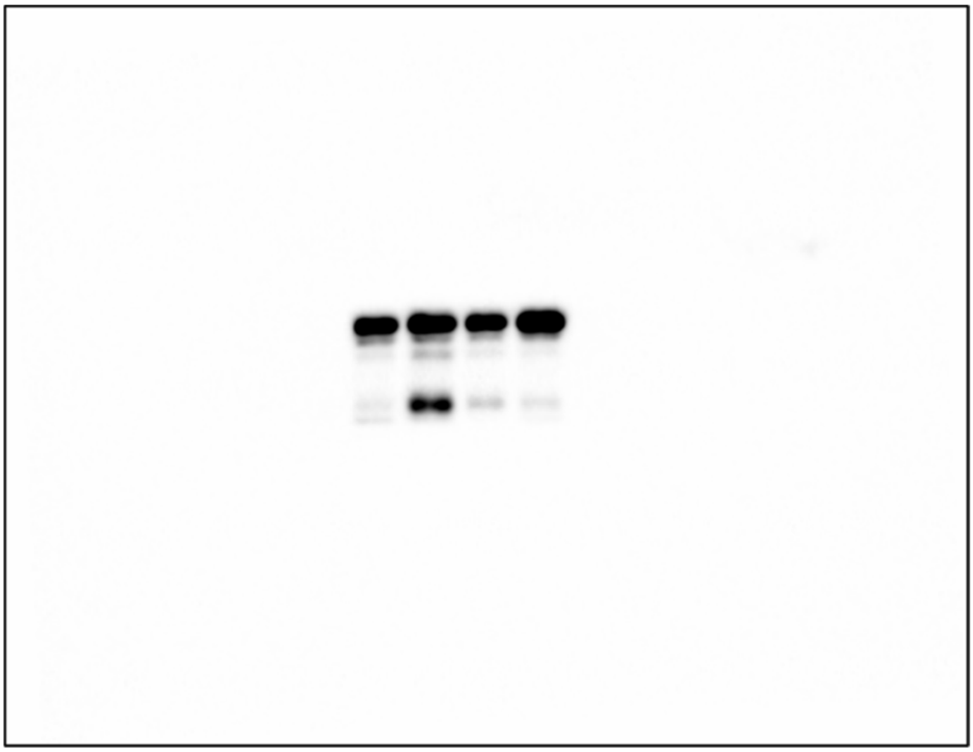

Figure 4F 7402 Occludin

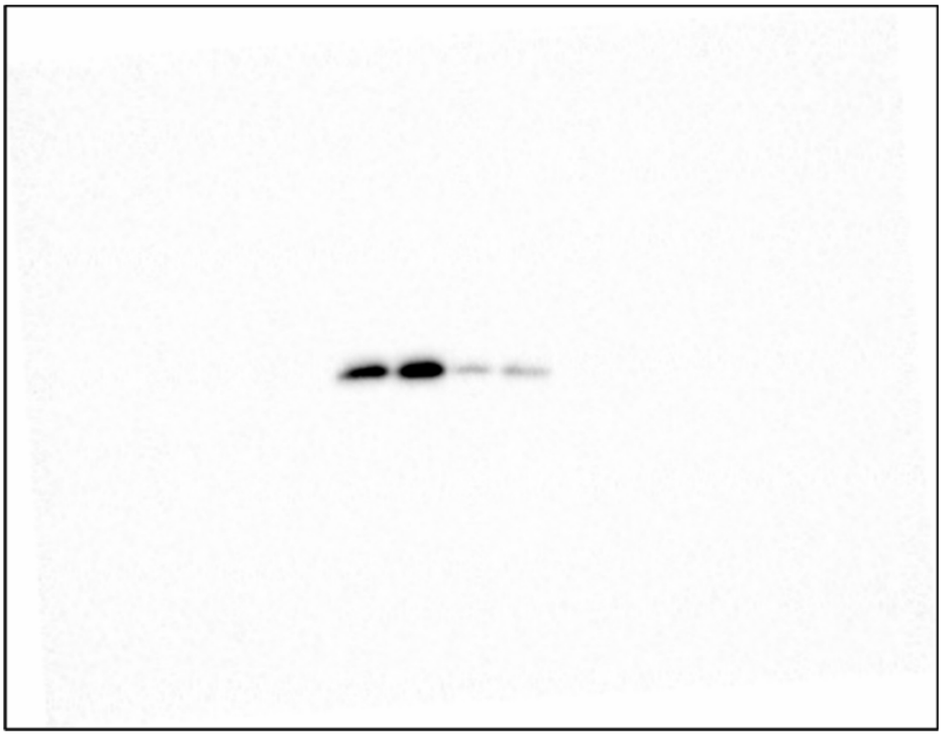

Figure 4F 7402 p21

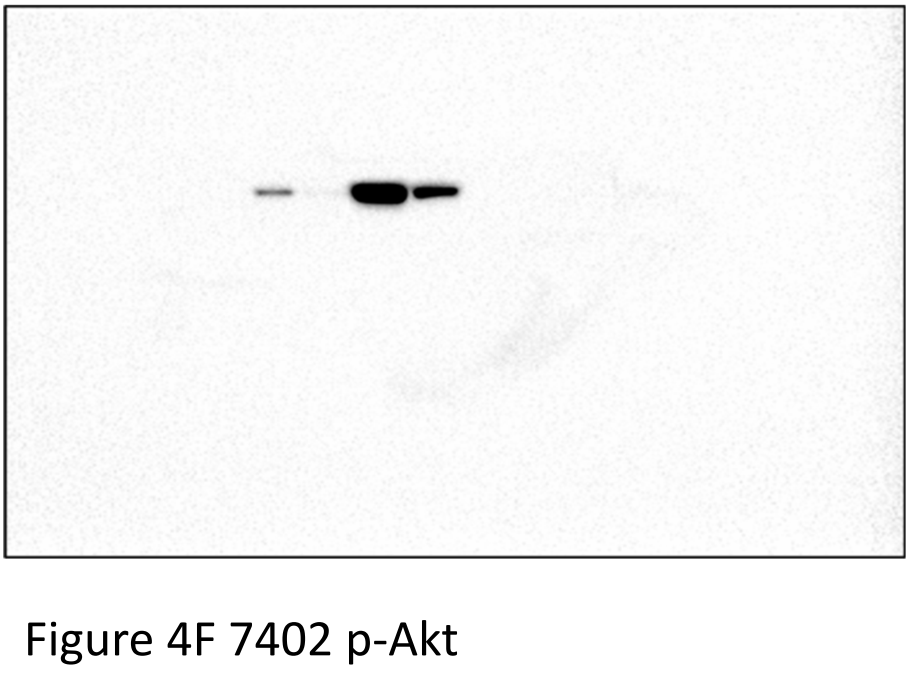

Figure 4F 7402 p-Akt

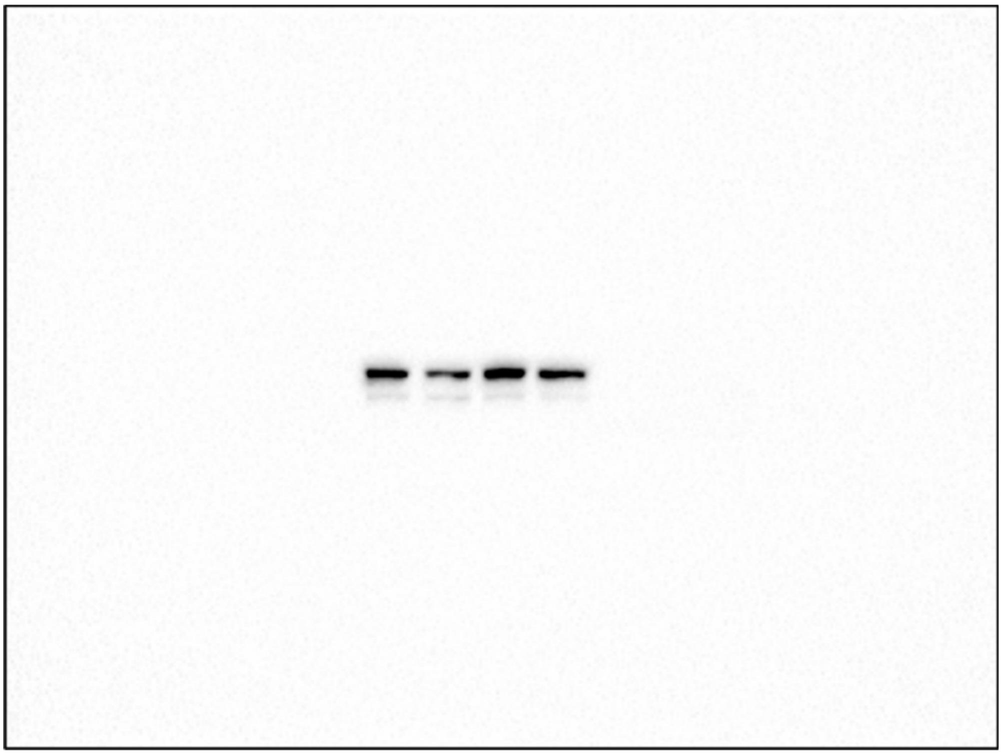

Figure 4F 7402 p-MTOR

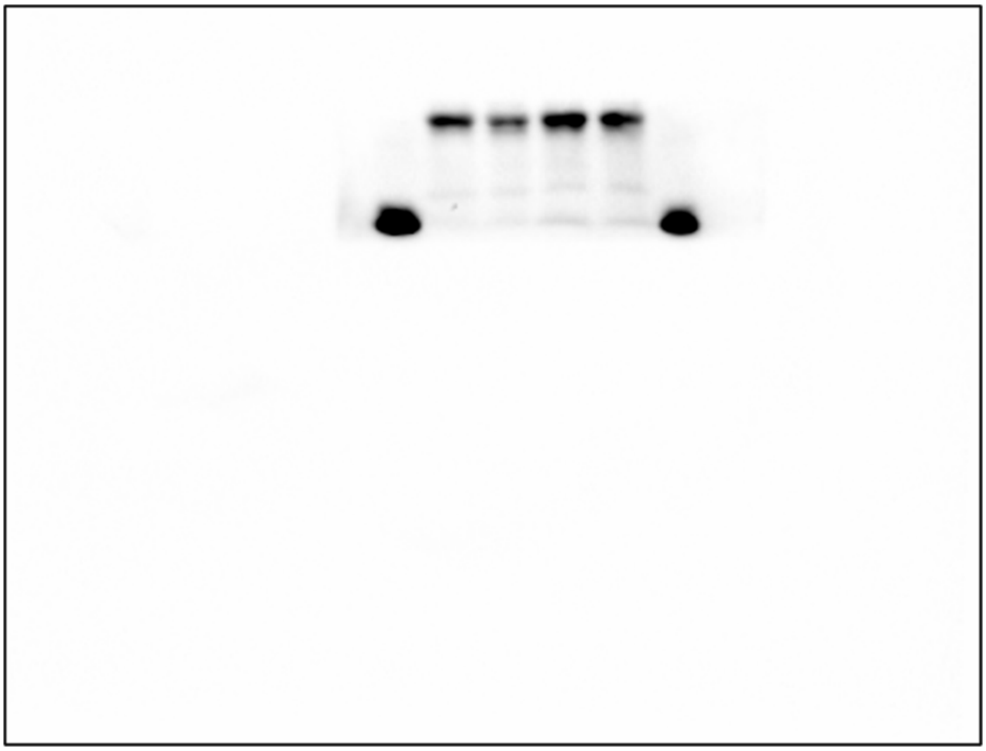

Figure 4F 7402 Snail

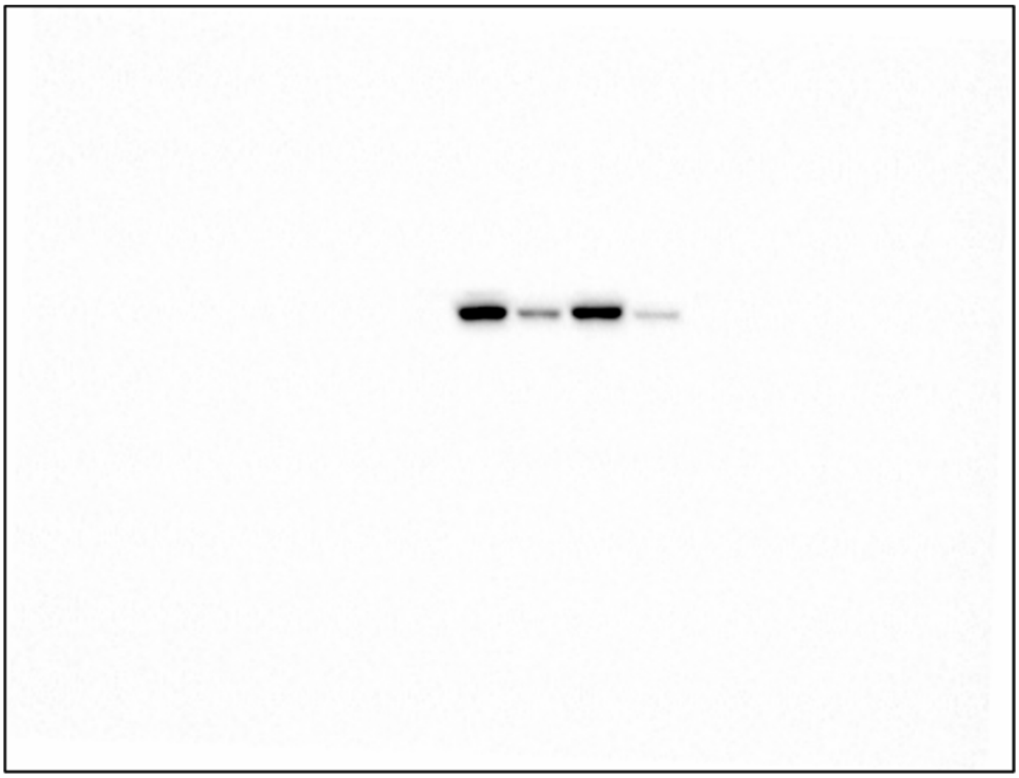

Figure 4F 7402 TXNRD1

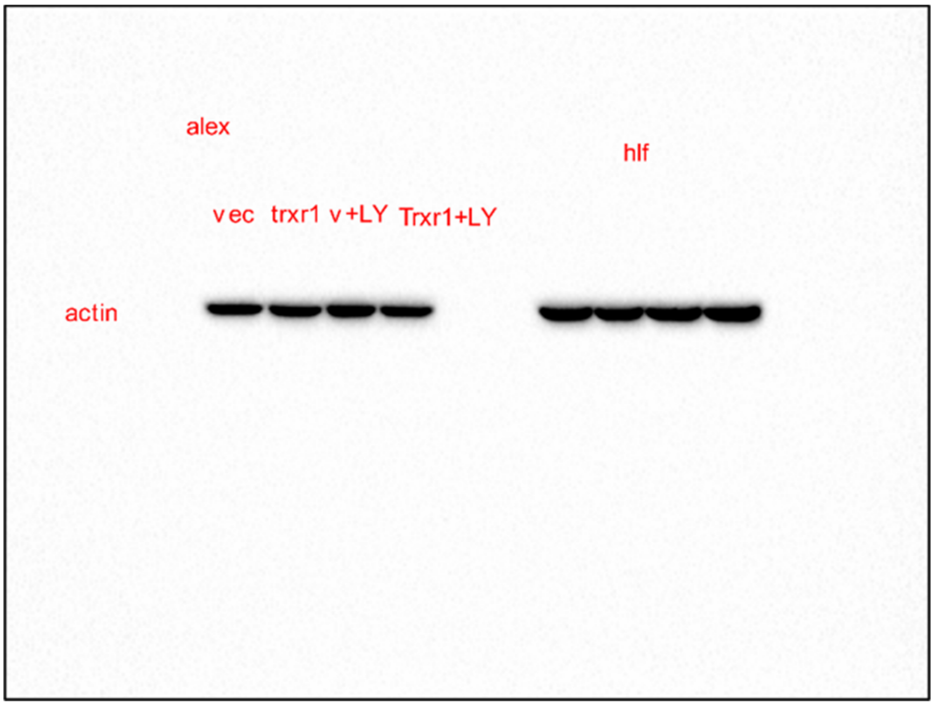

Figure 4F HLF actin

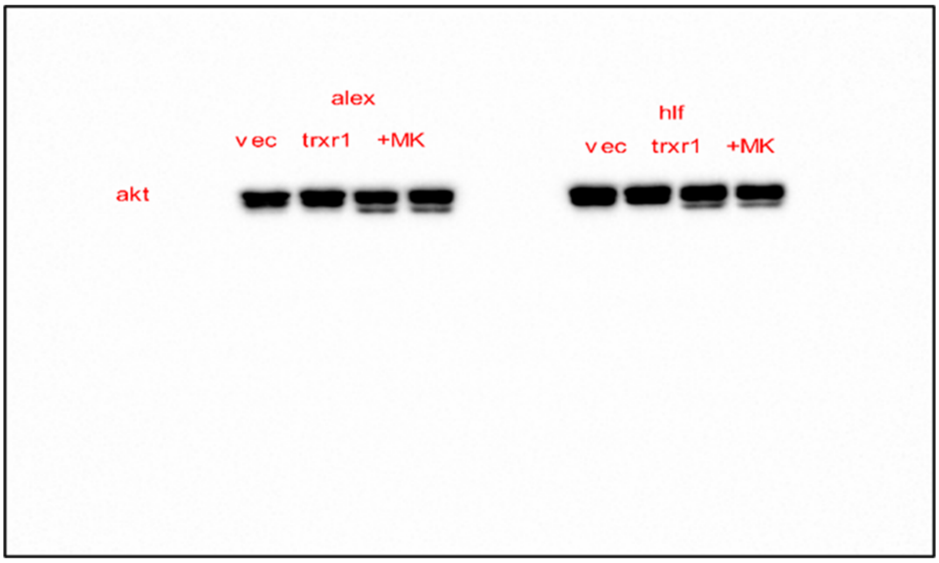

Figure 4F HLF Akt

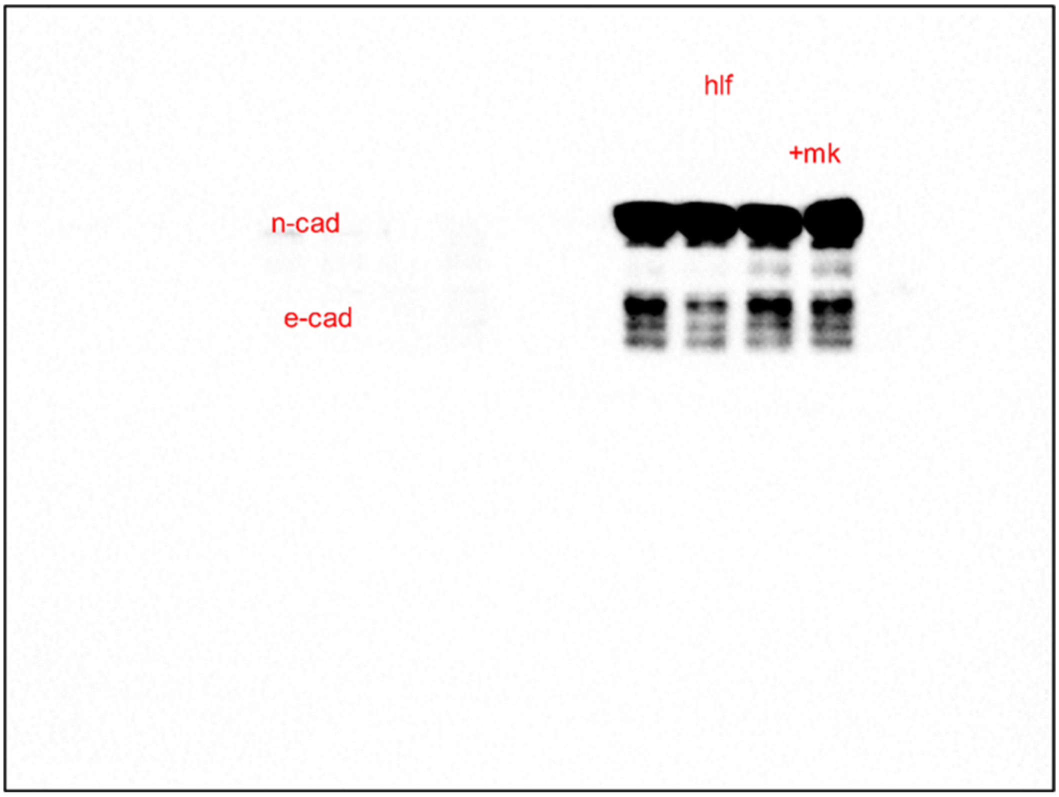

Figure 4F HLF E-cad

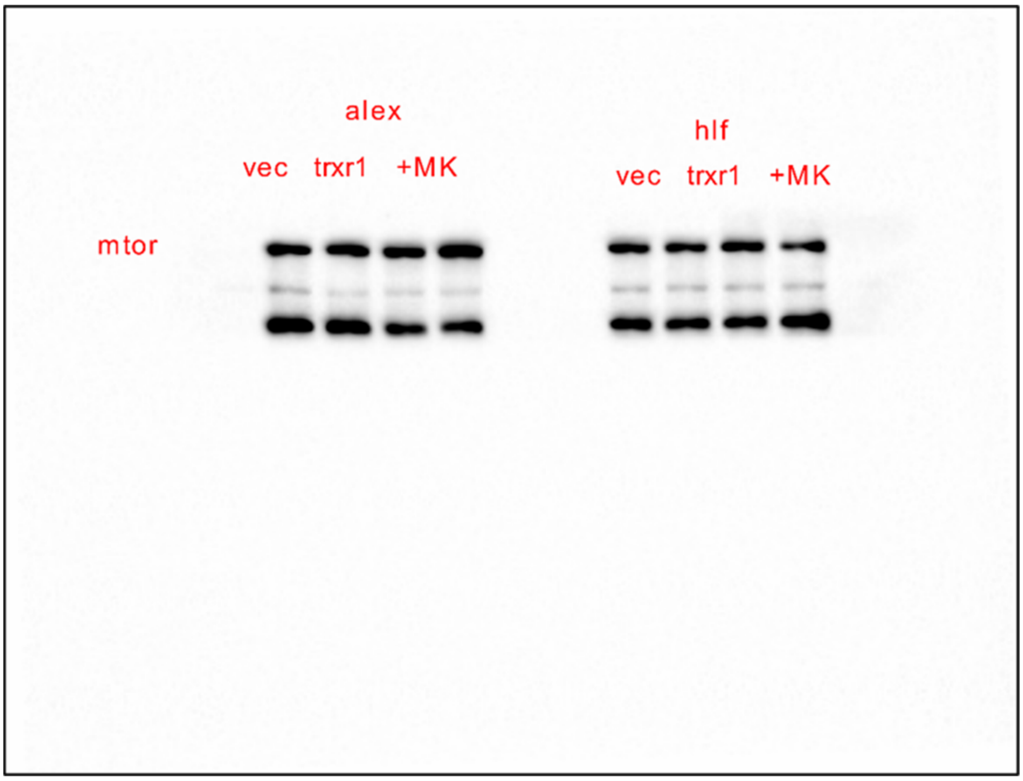

Figure 4F HLF MTOR

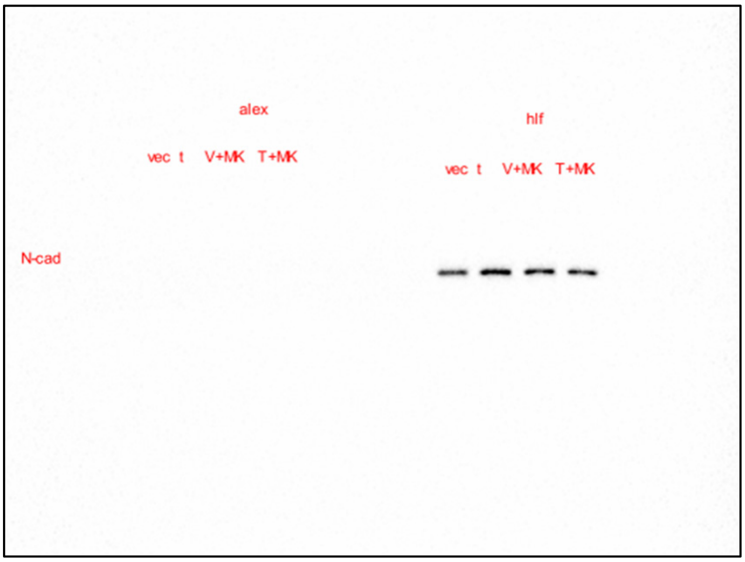

Figure 4F HLF N-cad

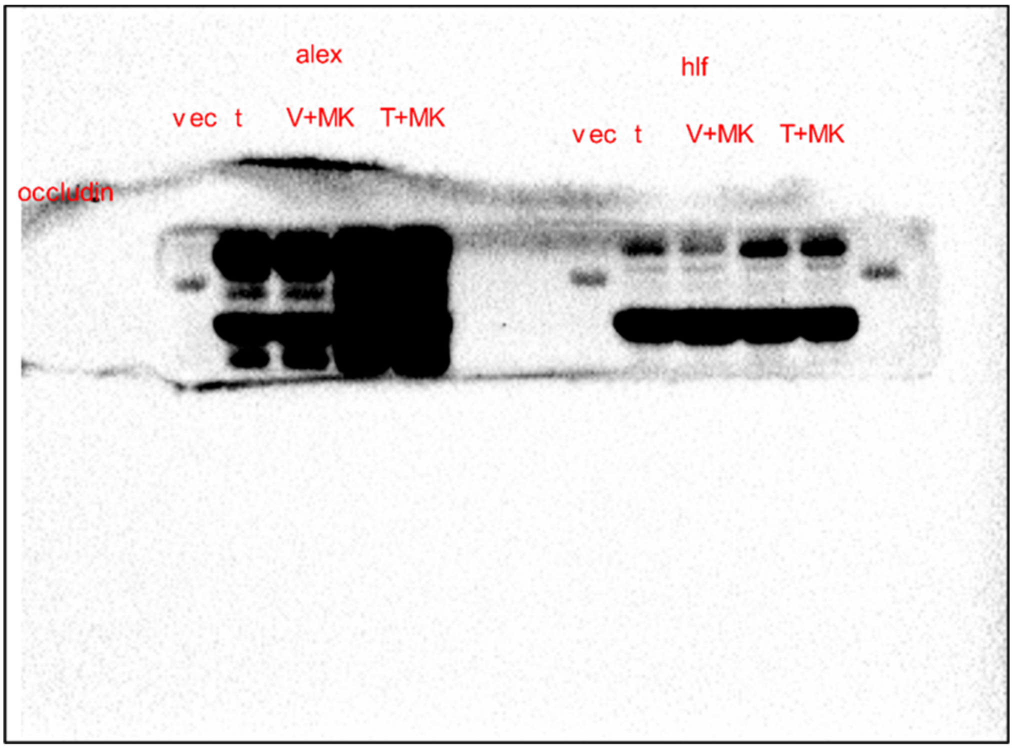

Figure 4F HLF Occludin

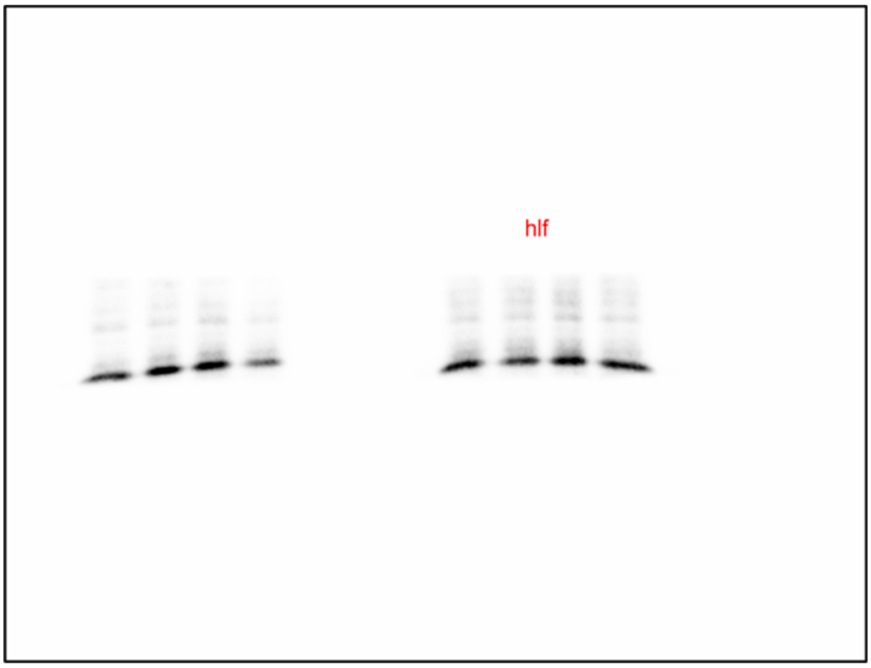

Figure 4F HLF p21

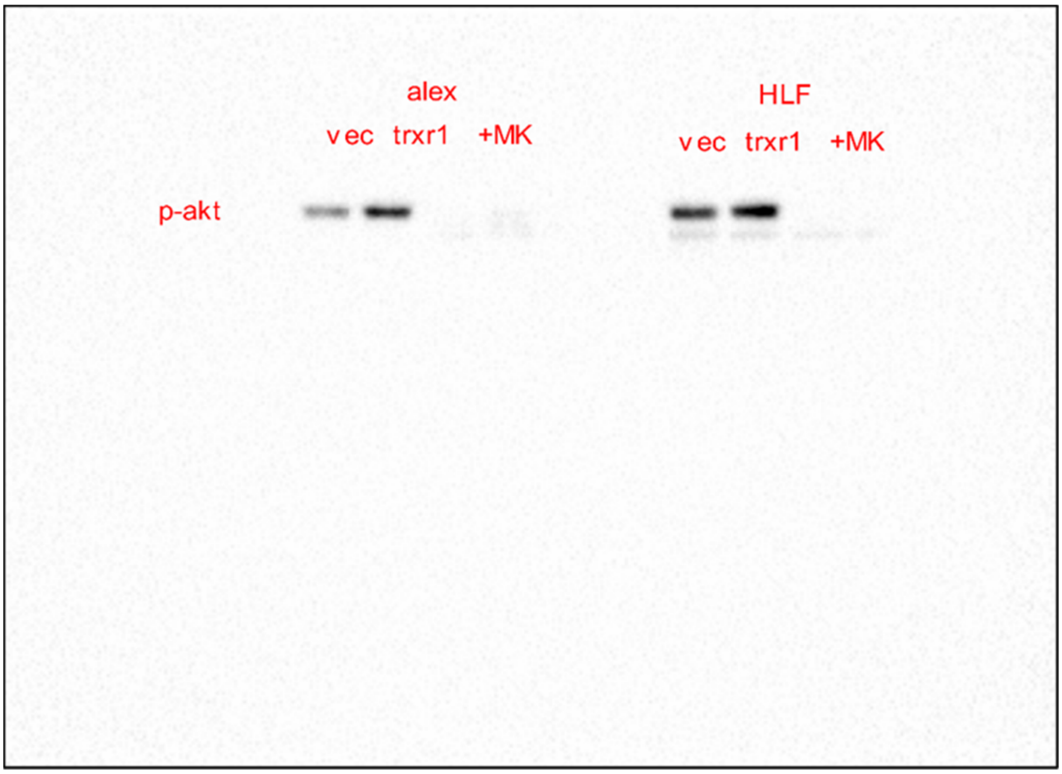

Figure 4F HLF p-Akt

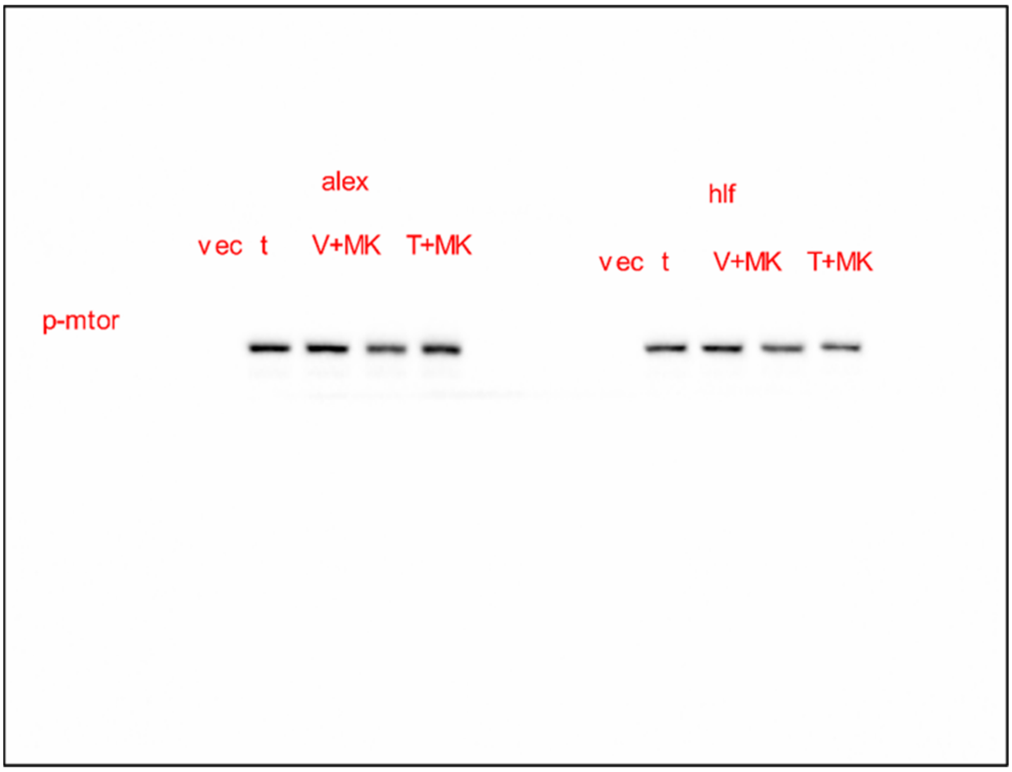

Figure 4F HLF p-MTOR

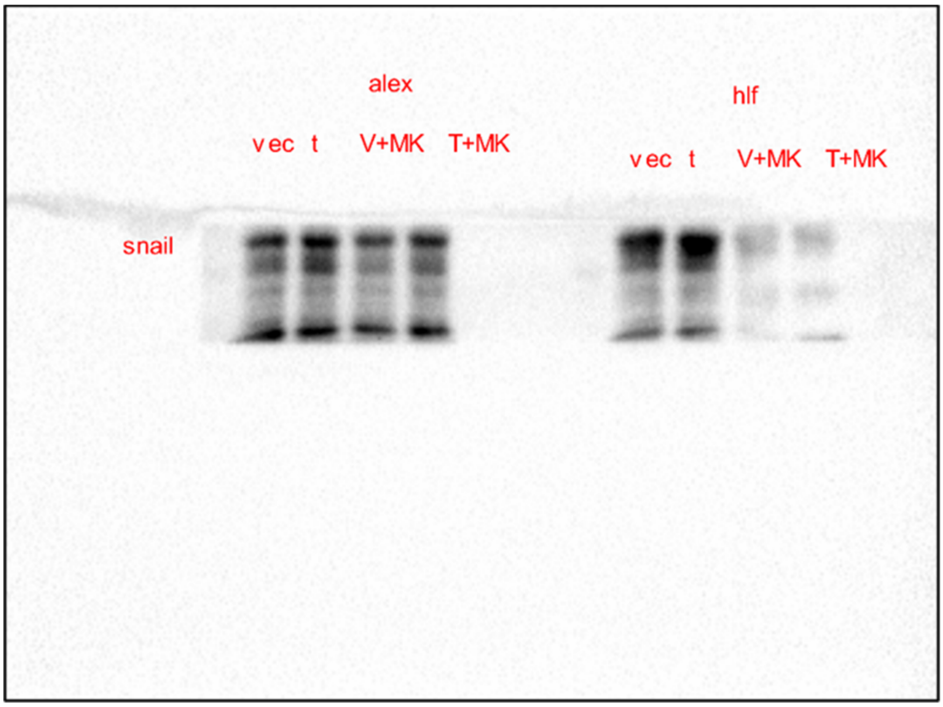

Figure 4F HLF Snail

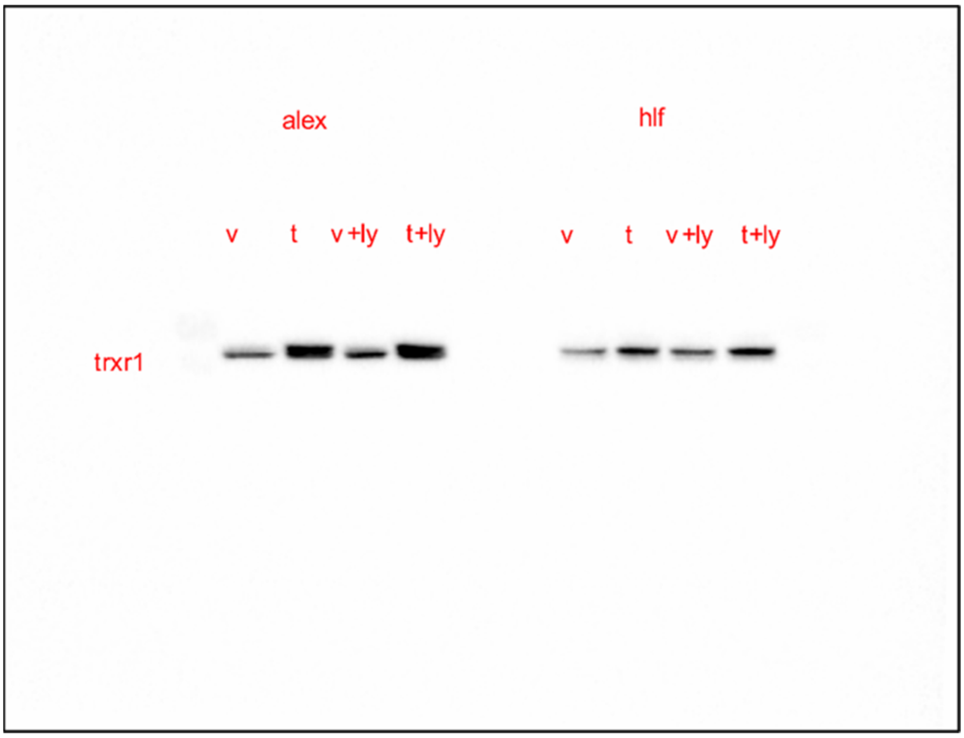

Figure 4F HLF TXNRD1

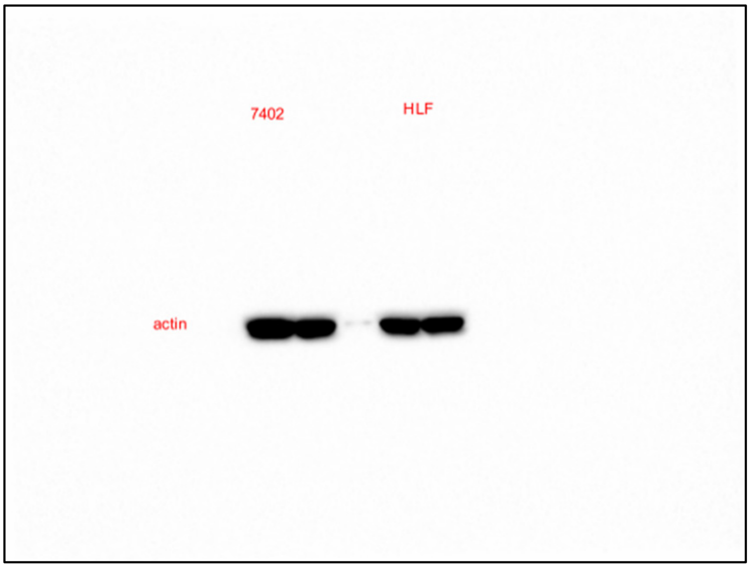

Figure 4G 7402 actin

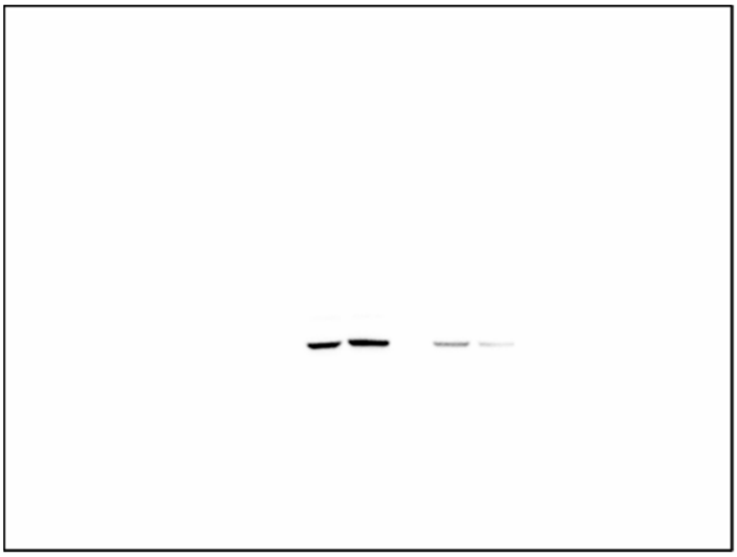

Figure 4G 7402 PTEN

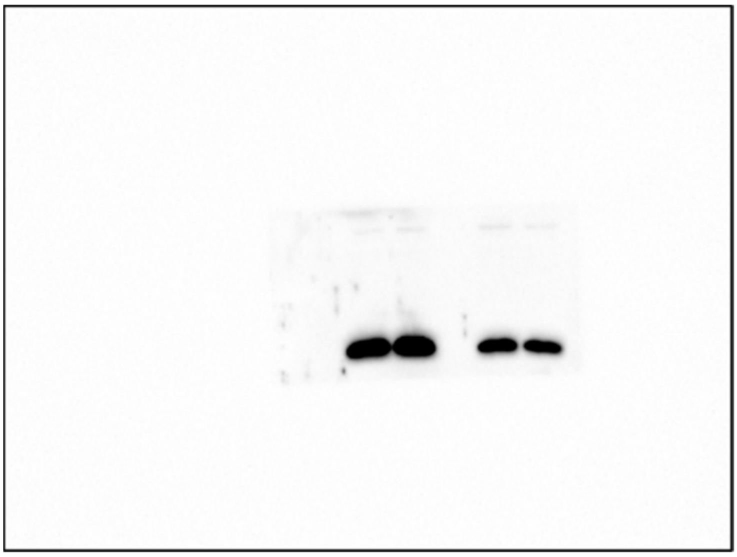

Figure 4G 7402 Trx

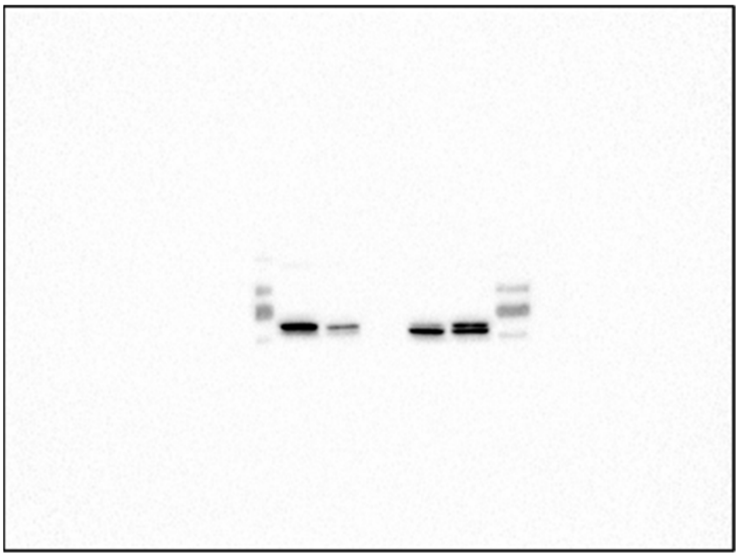

Figure 4G 7402 TXNRD1

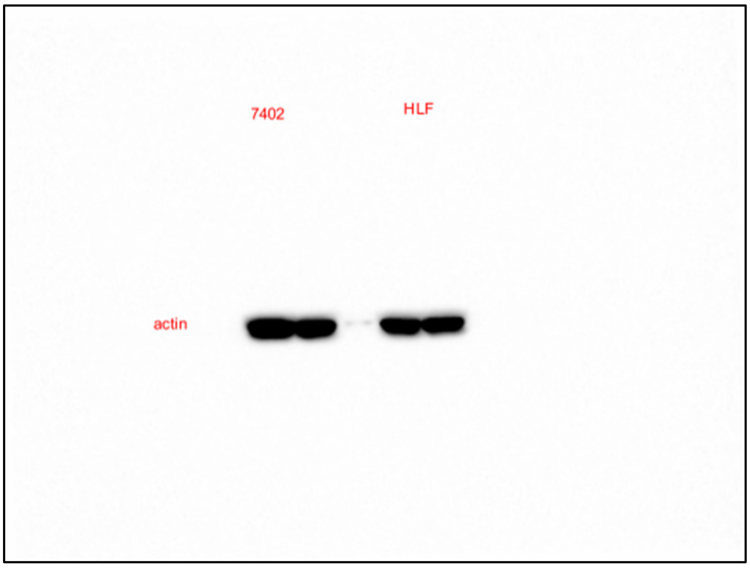

Figure 4G HLF actin

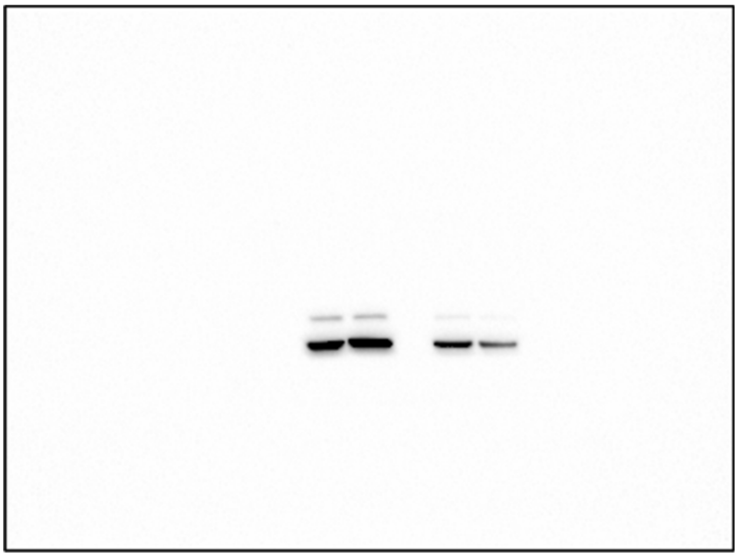

Figure 4G HLF PTEN

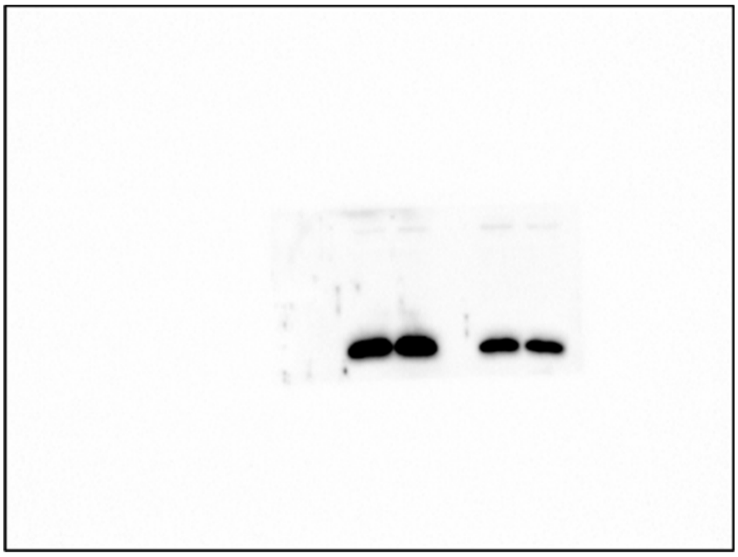

Figure 4G HLF Trx

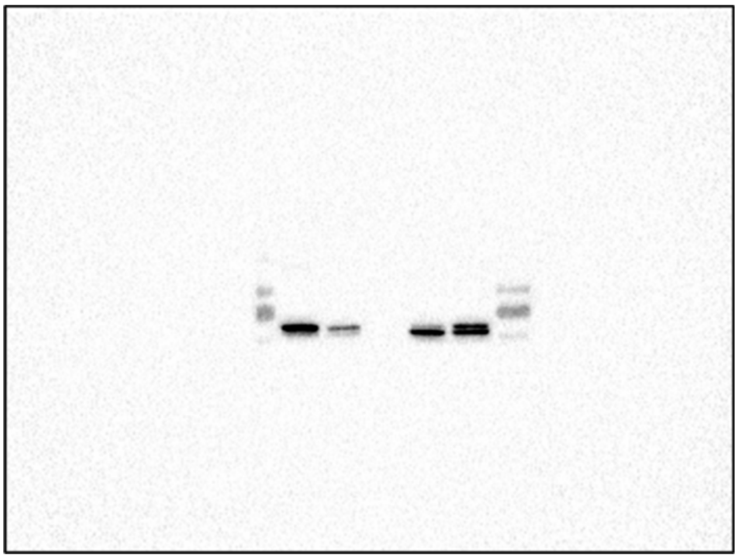

Figure 4G HLF TXNRD1

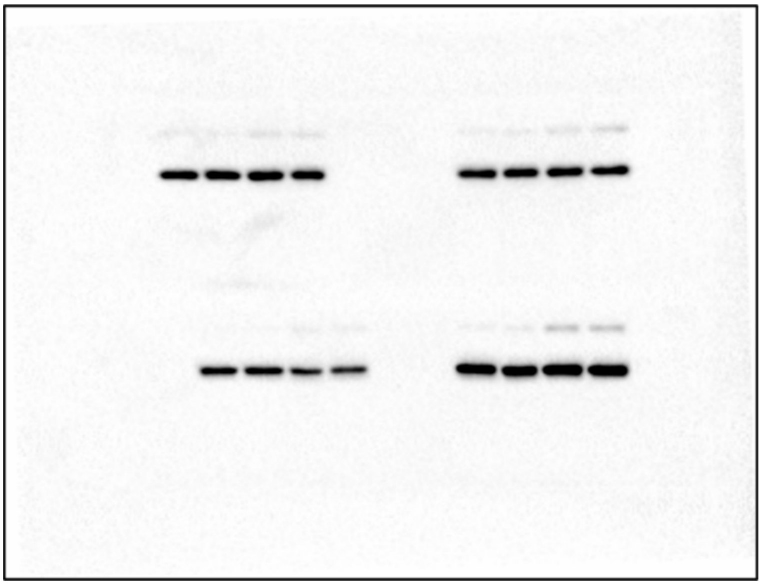

Figure 4H 7402 Input PTEN

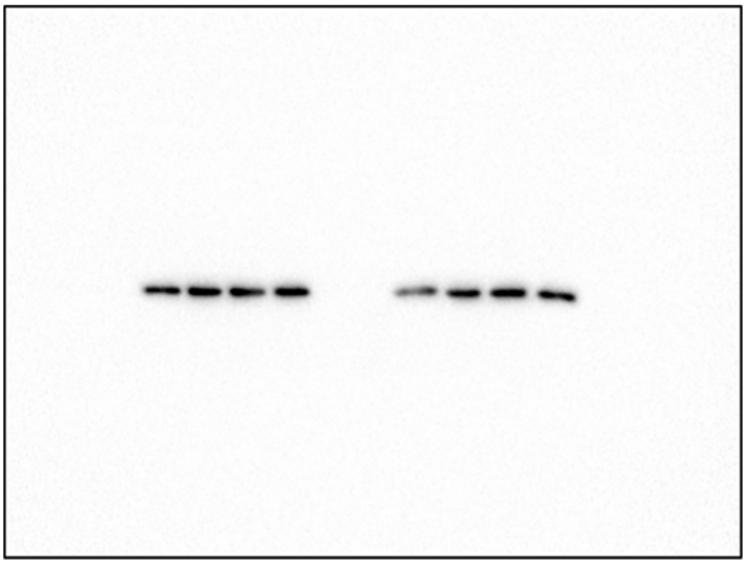

Figure 4H 7402 Input Trx1

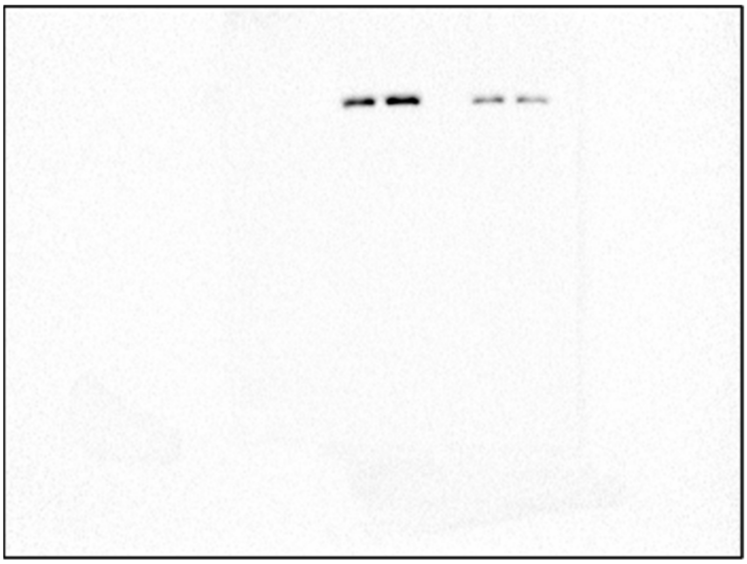

Figure 4H 7402 IP Trx1 PTEN

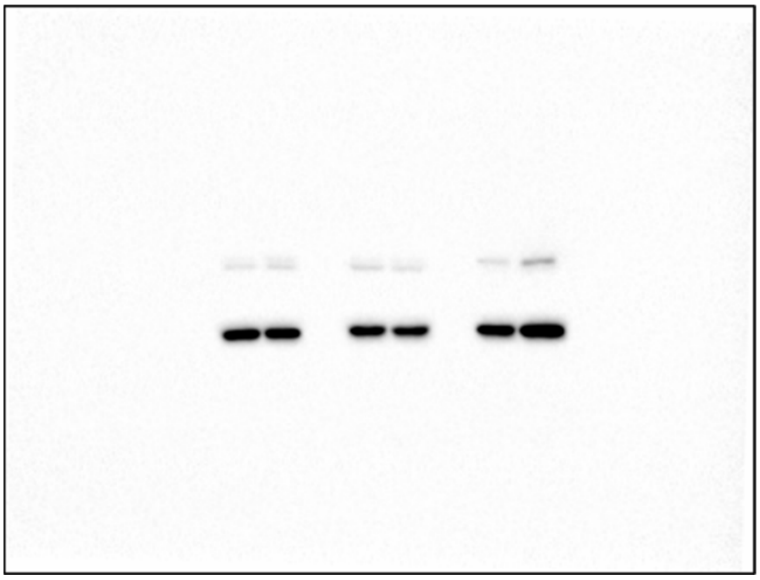

Figure 4H 7402 IP Trx1 Trx1

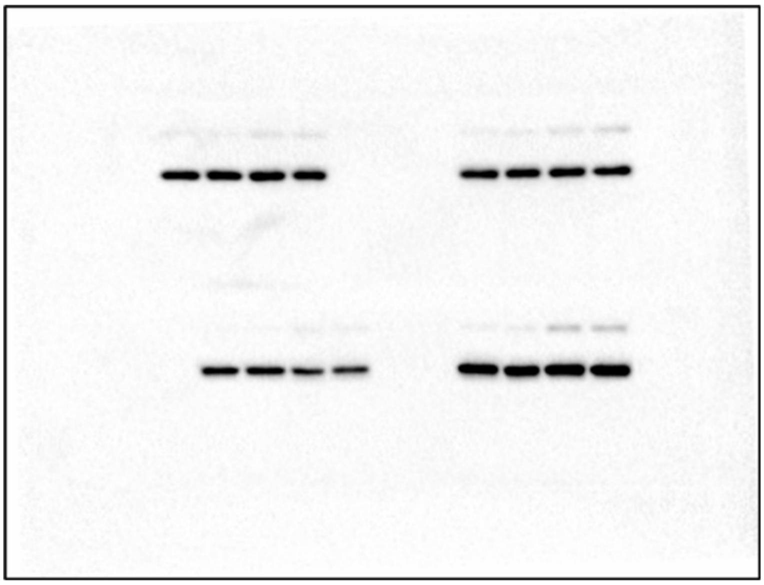

Figure 4H HLF Input PTEN

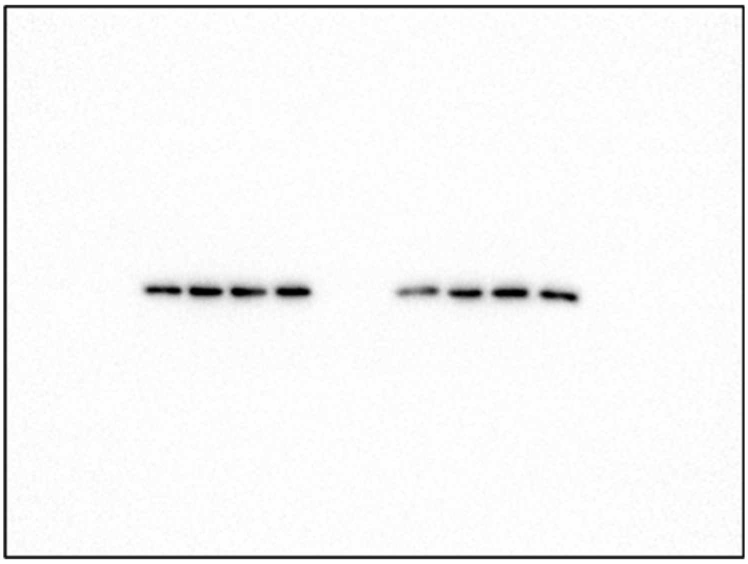

Figure 4H HLF Input Trx1

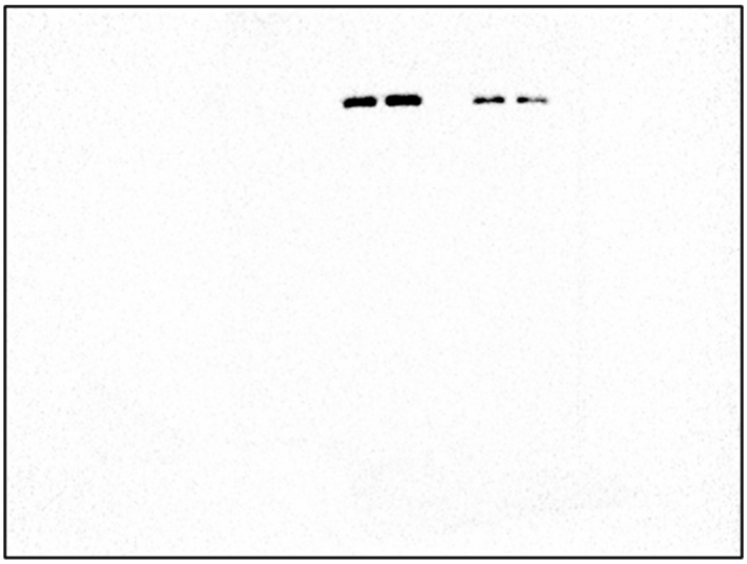

Figure 4H HLF IP Trx1 PTEN

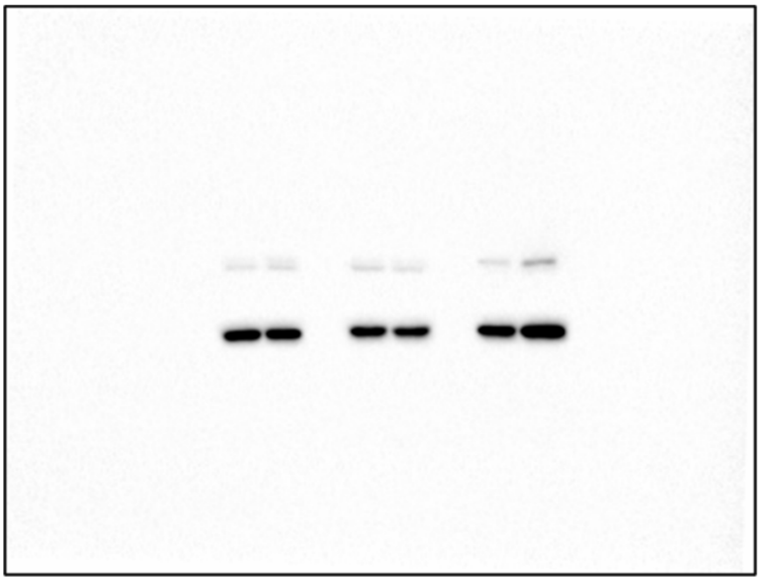

Figure 4H HLF IP Trx1 Trx1

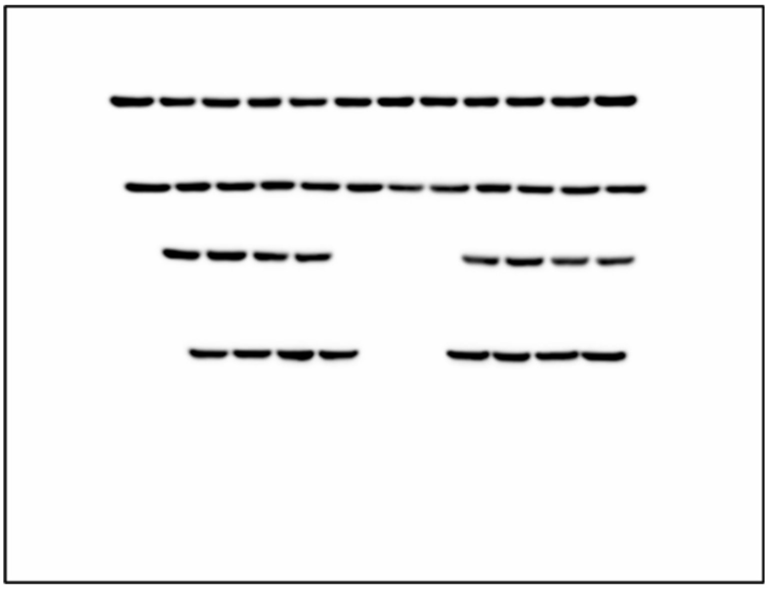

Figure 4I 7402shCon actin

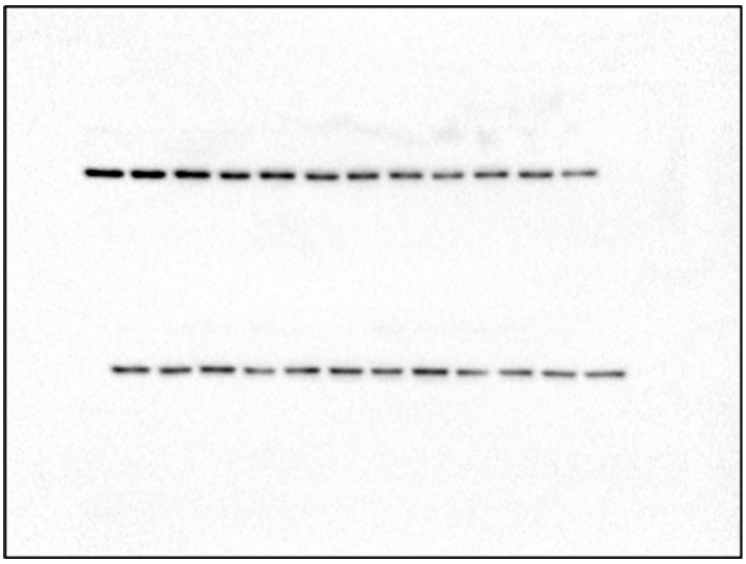

Figure 4I 7402shCon PTEN

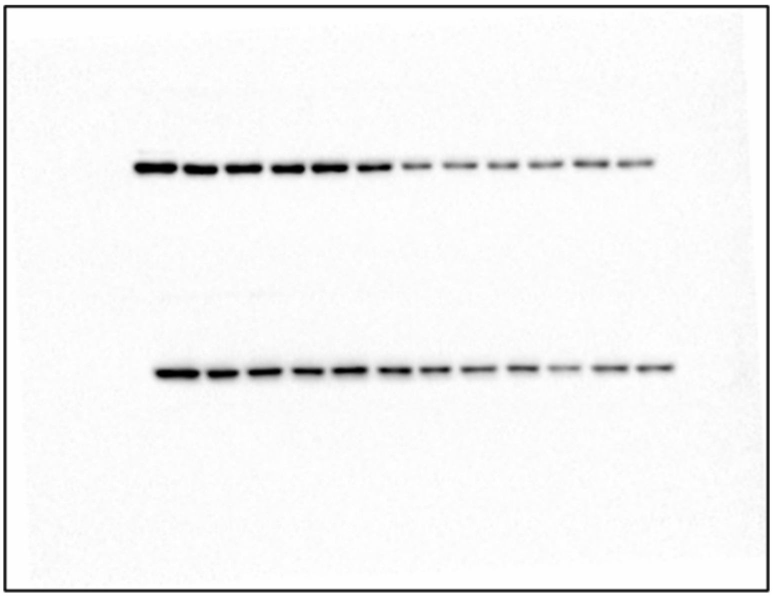

Figure 4I 7402shCon TXNRD1

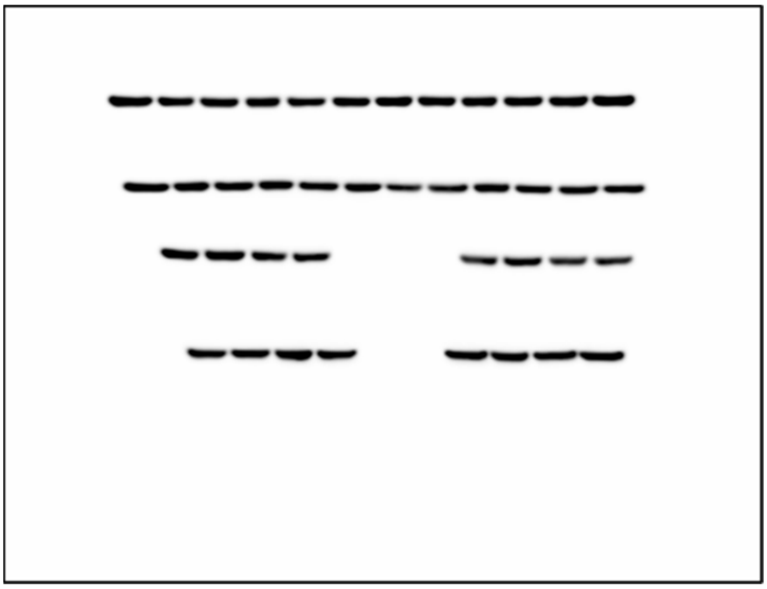

Figure 4I 7402shTXNRD1 actin

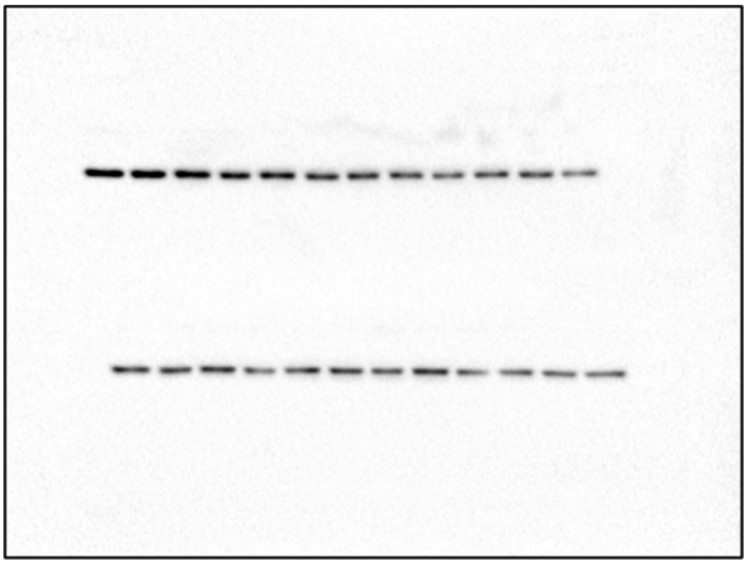

Figure 4I 7402shTXNRD1 PTEN

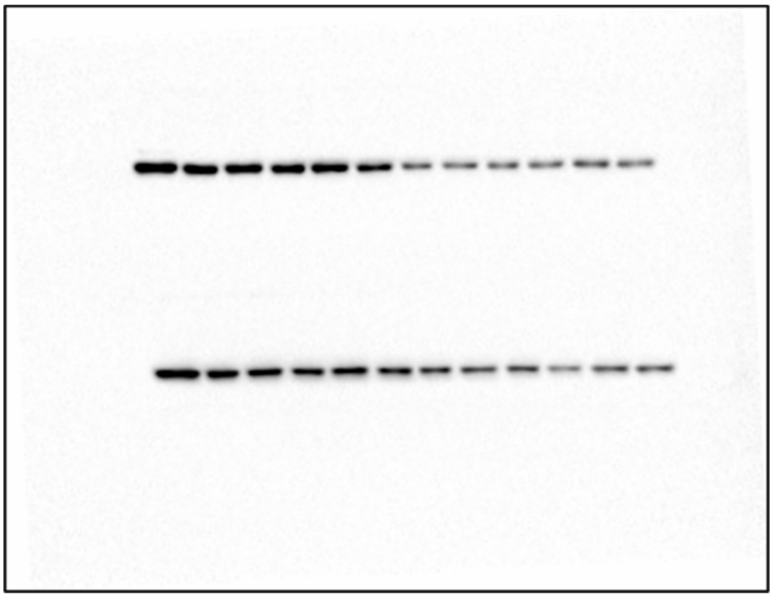

Figure 4I 7402shTXNRD1 TXNRD1

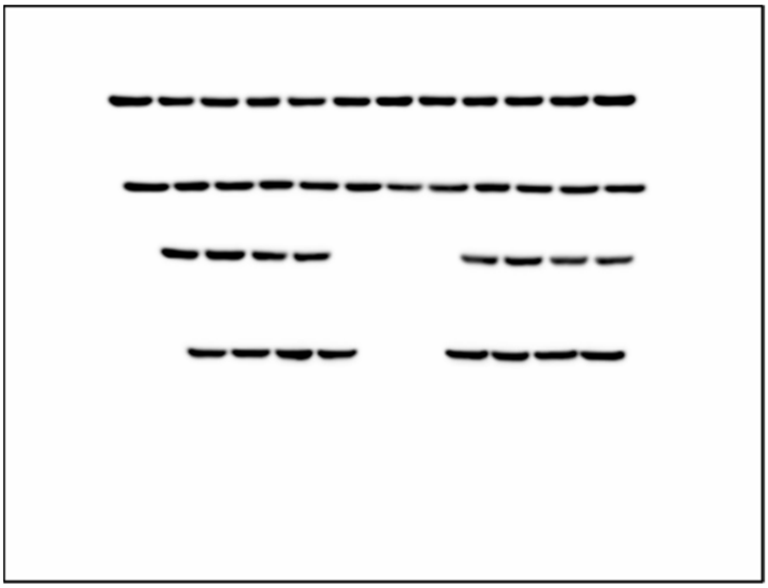

Figure 4I HLF TXNRD1 actin

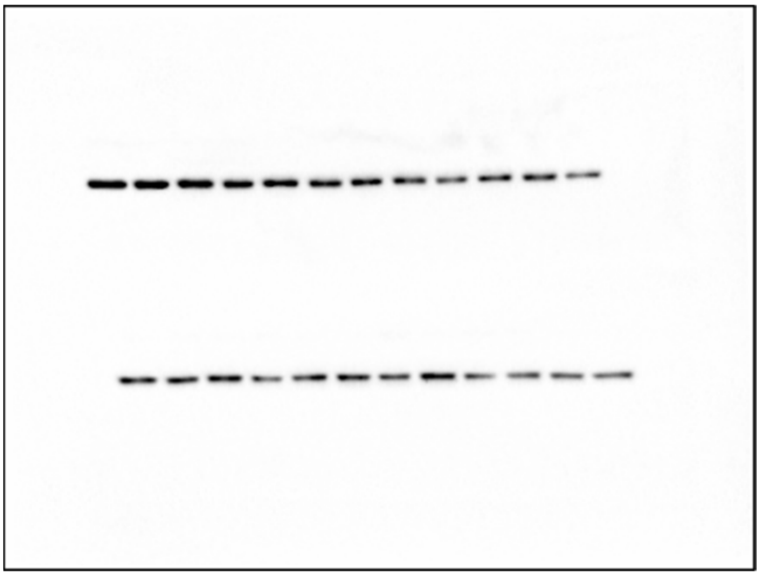

Figure 4I HLF TXNRD1 PTEN

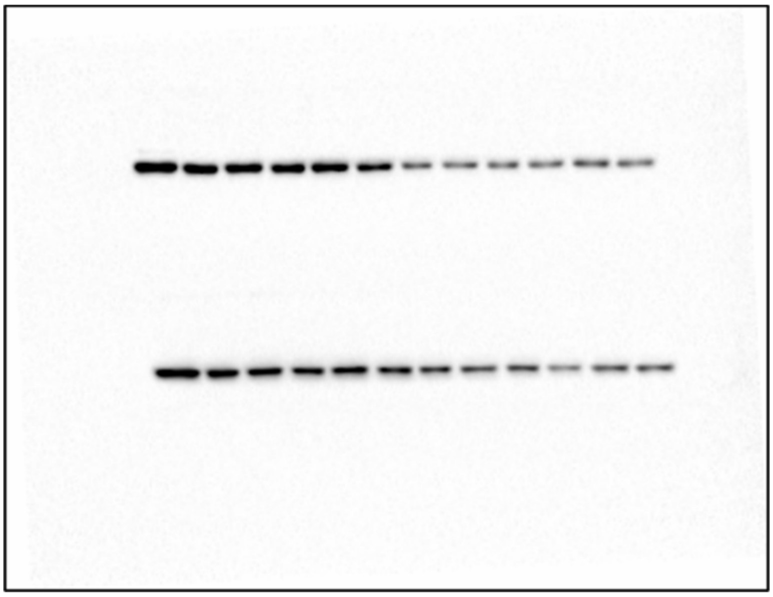

Figure 4I HLF TXNRD1 TXNRD1

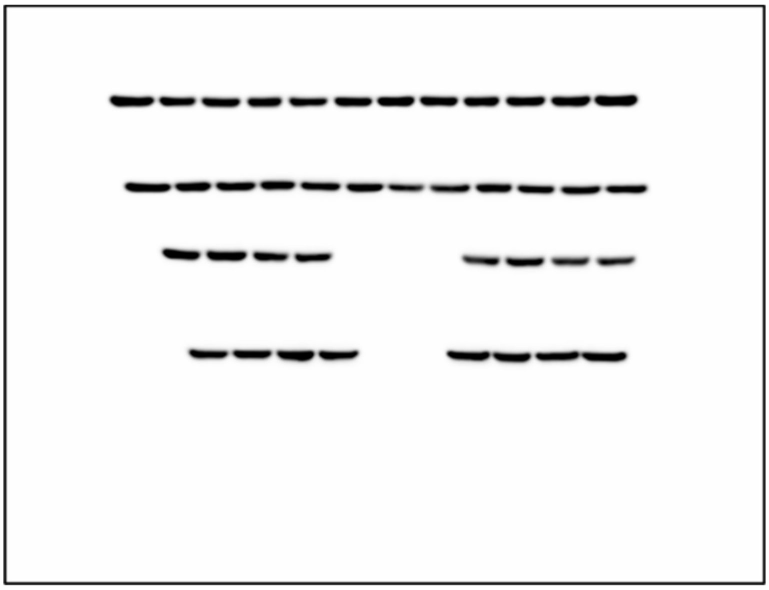

Figure 4I HLF vector actin

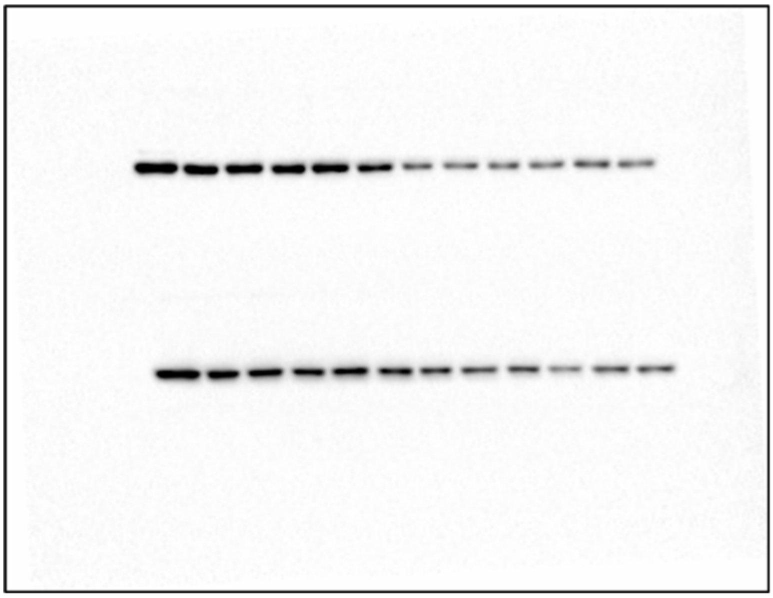

Figure 4I HLF vector TXNRD1

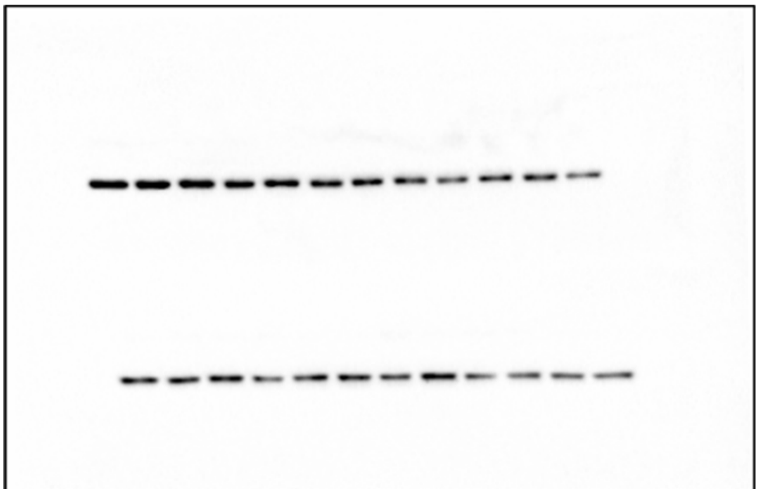

Figure 4I HLF vector PTEN

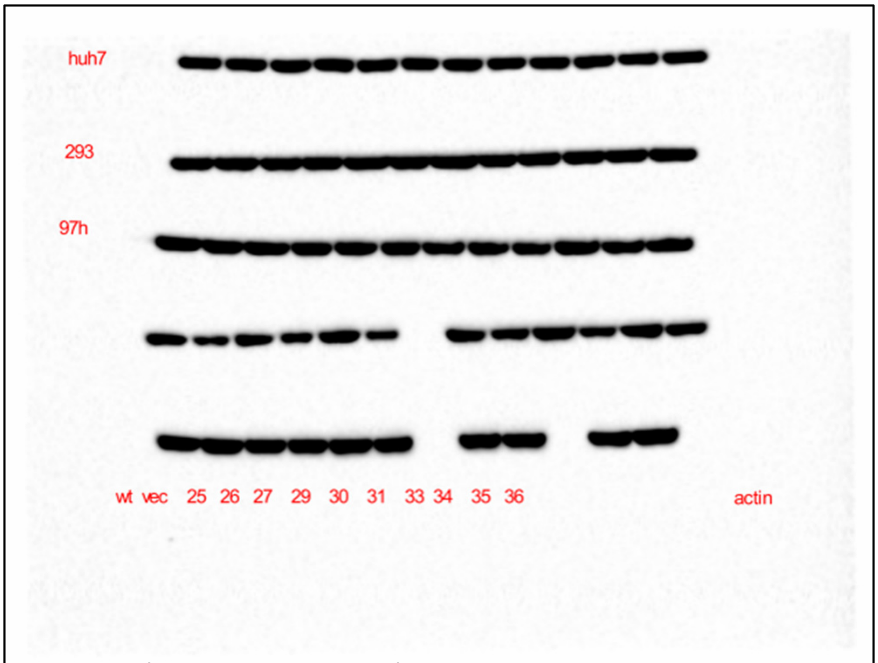

Figure 5C actin

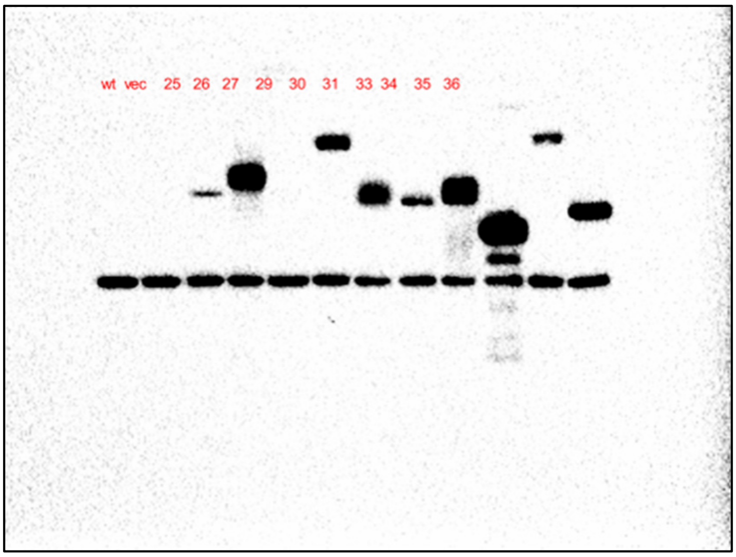

Figure 5C Flag

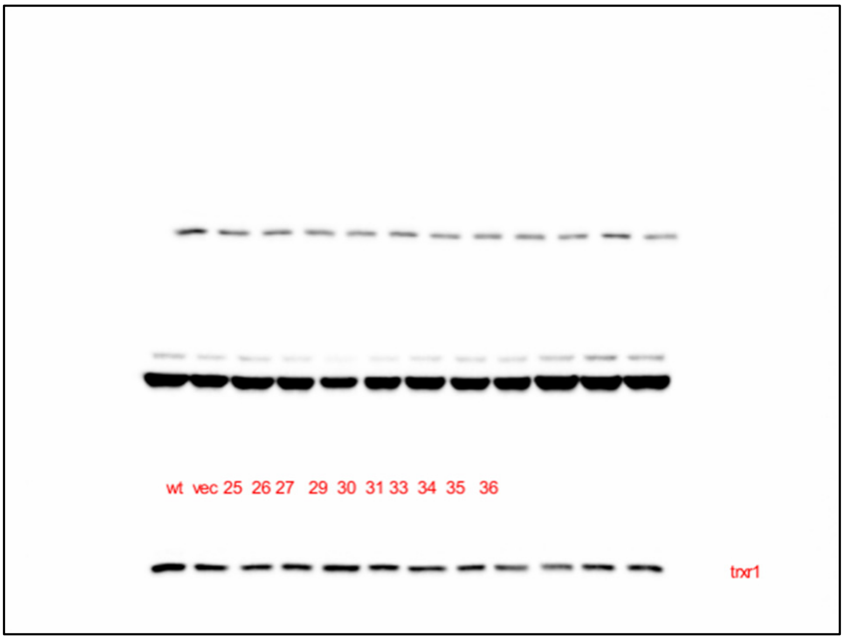

Figure 5C TXNRD1

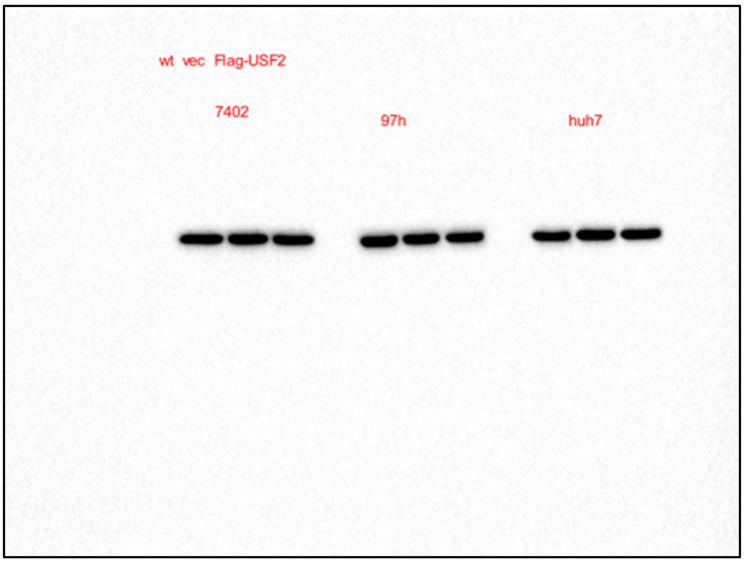

Figure 5D 7402 actin

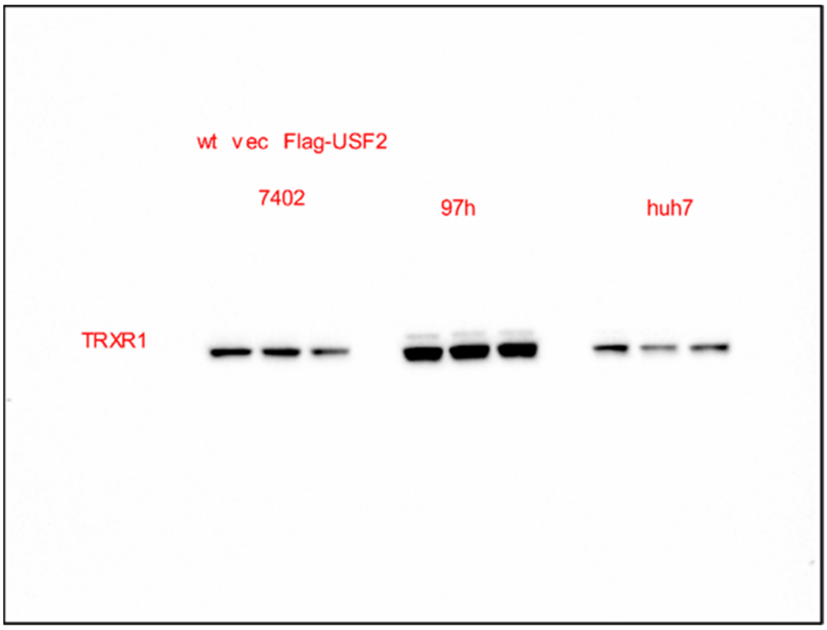

Figure 5D 7402 TXNRD1

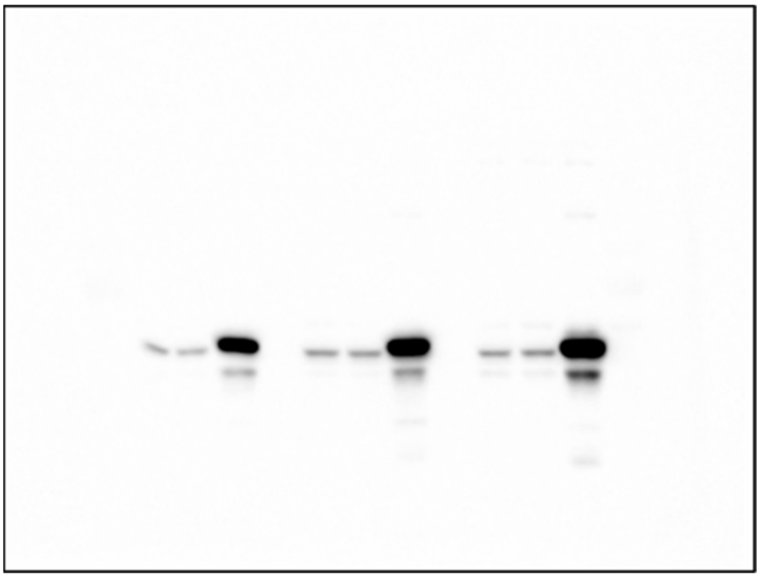

Figure 5D 7402 USF2

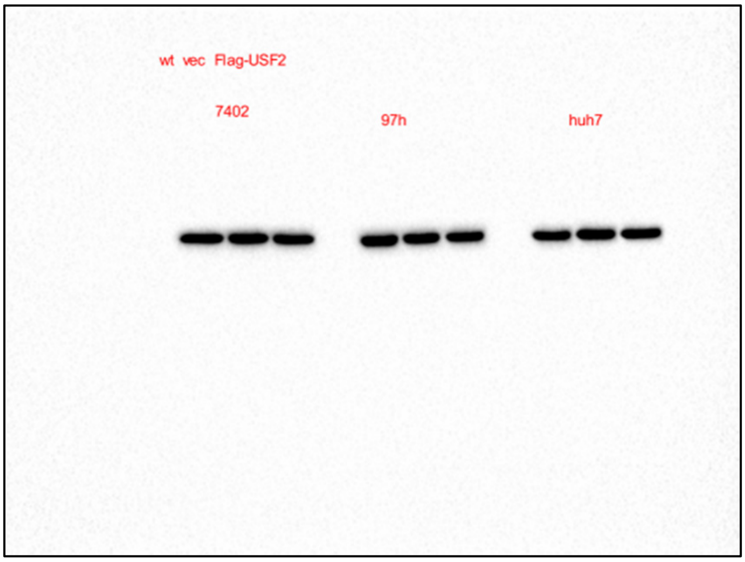

Figure 5D Huh7 actin

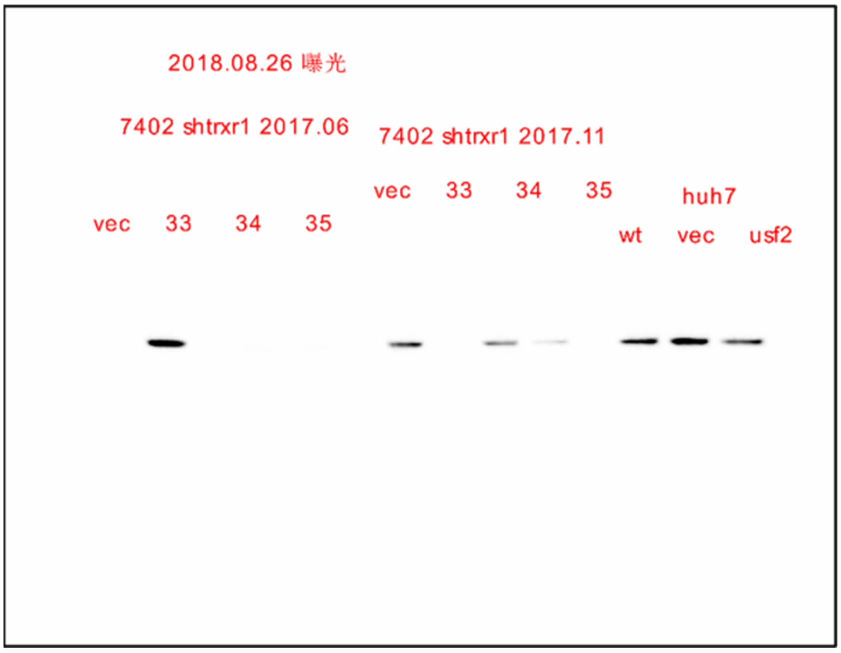

Figure 5D Huh7 TXNRD1

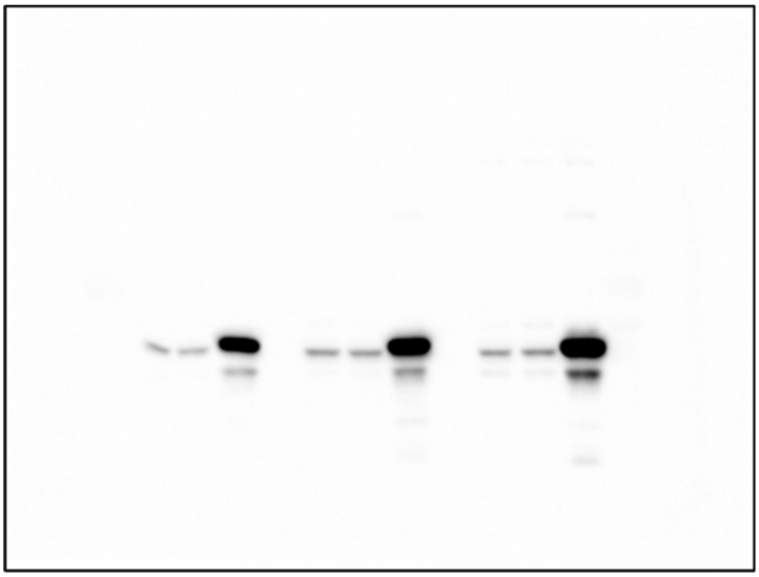

Figure 5D Huh7 USF2

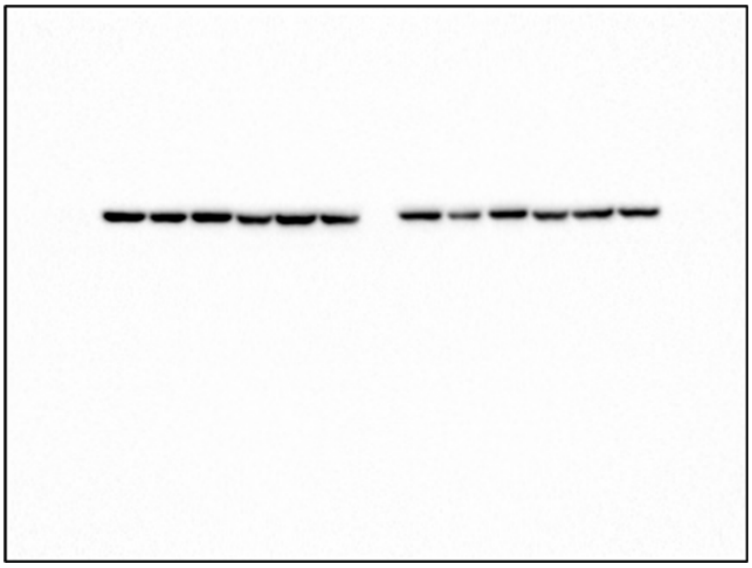

Figure 6E 7402 actin

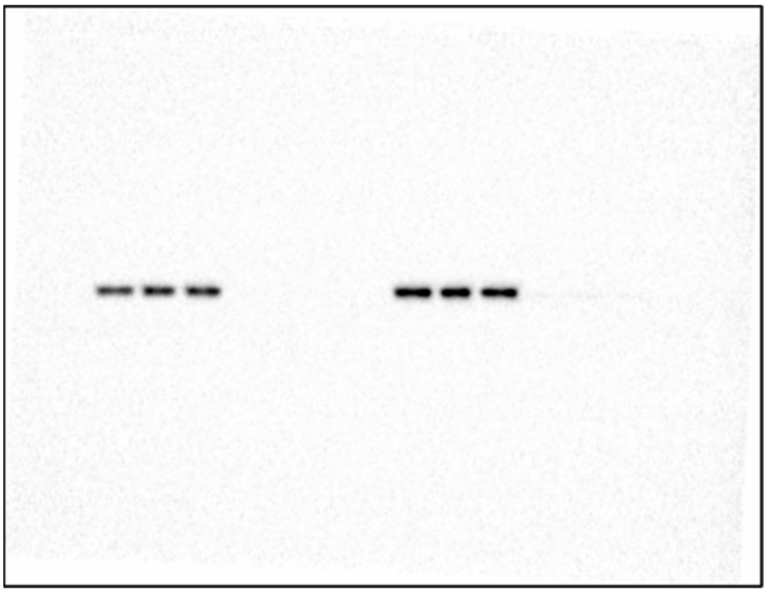

Figure 6E 7402 akt

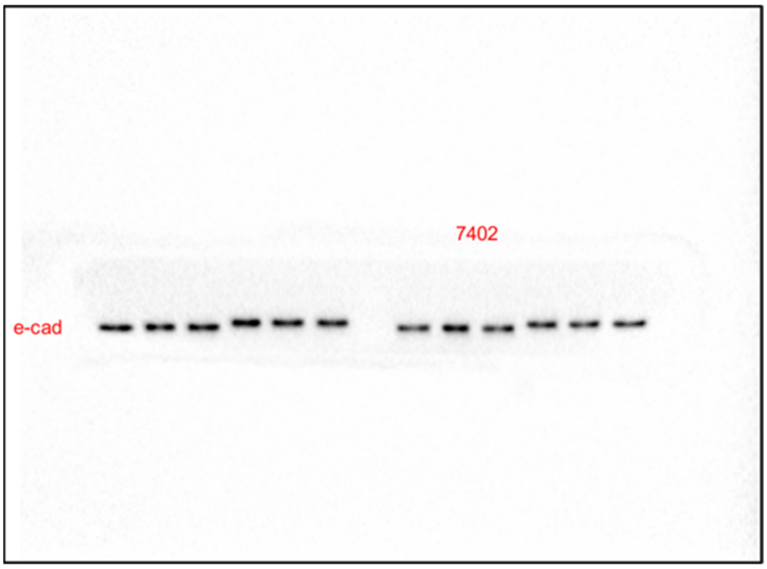

Figure 6E 7402 e-cad

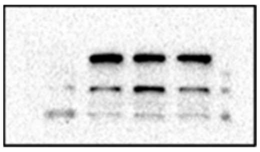

Figure 6E 7402 mTOR

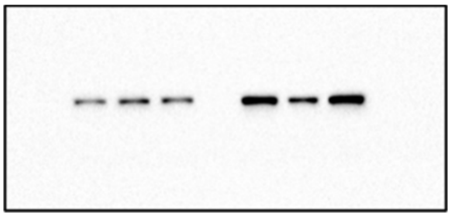

Figure 6E 7402 N-cad

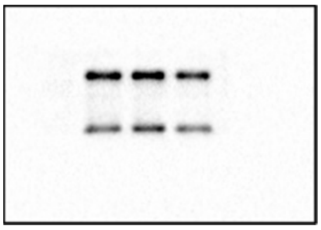

Figure 6E 7402 Occludin

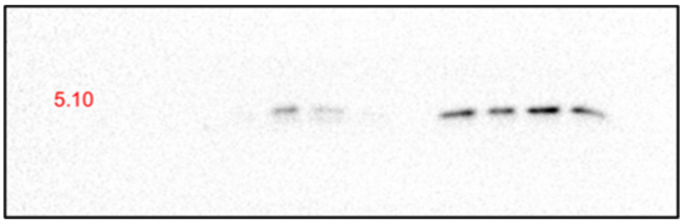

Figure 6E 7402 p21

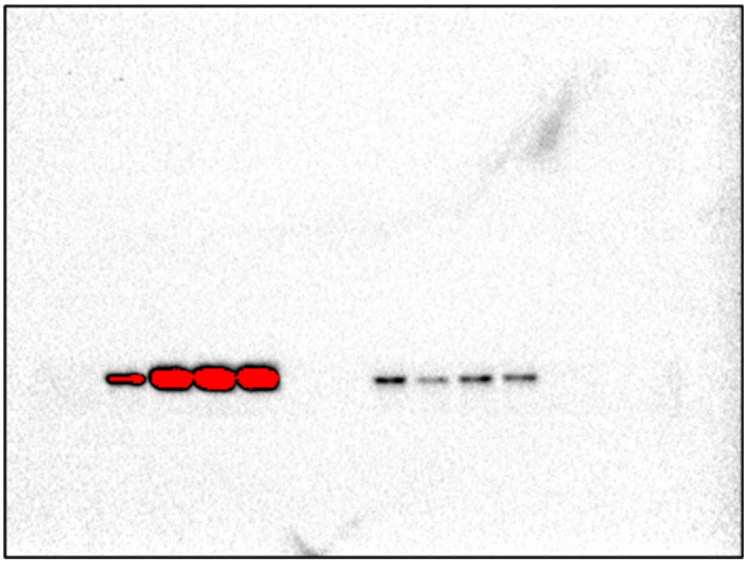

Figure 6E 7402 p-akt

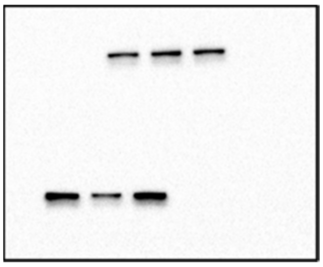

Figure 6E 7402 p-MTOR

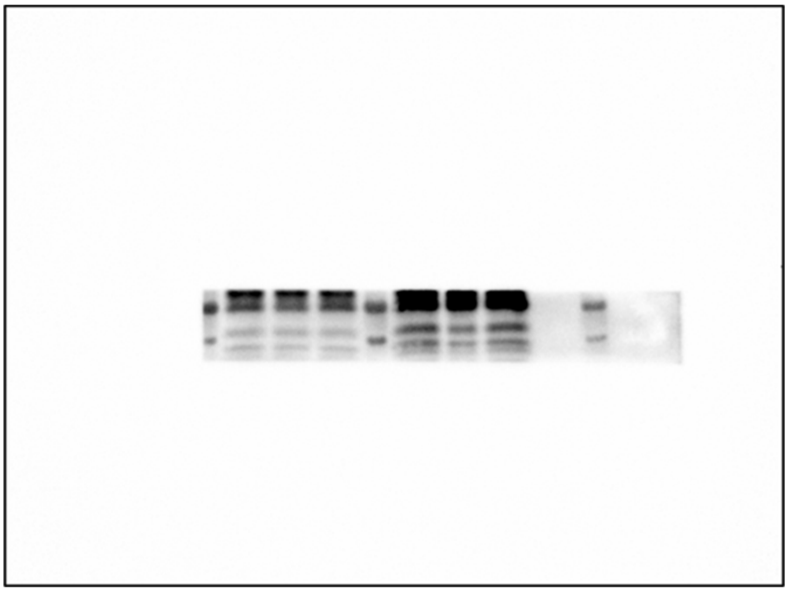

Figure 6E 7402 Snail

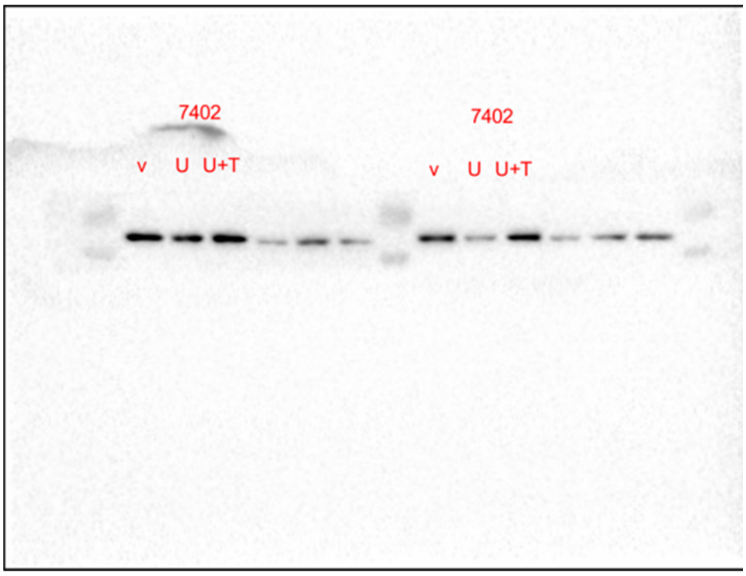

Figure 6E 7402 TXNRD1

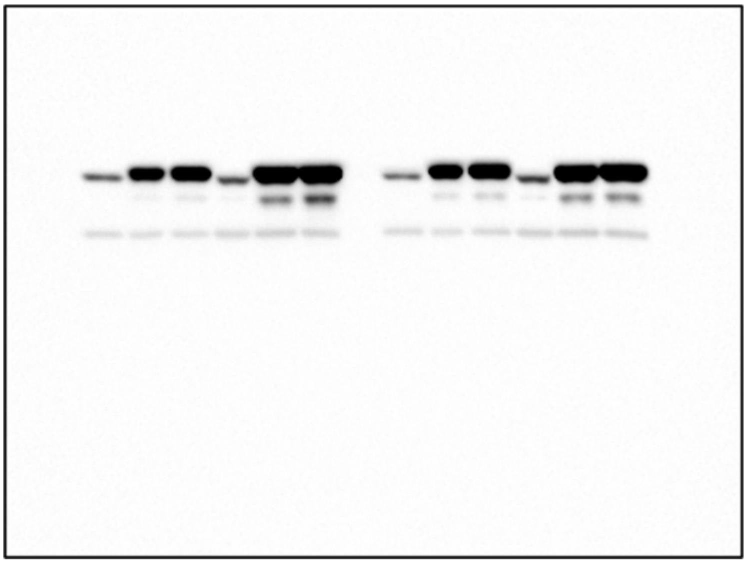

Figure 6E 7402 USF2

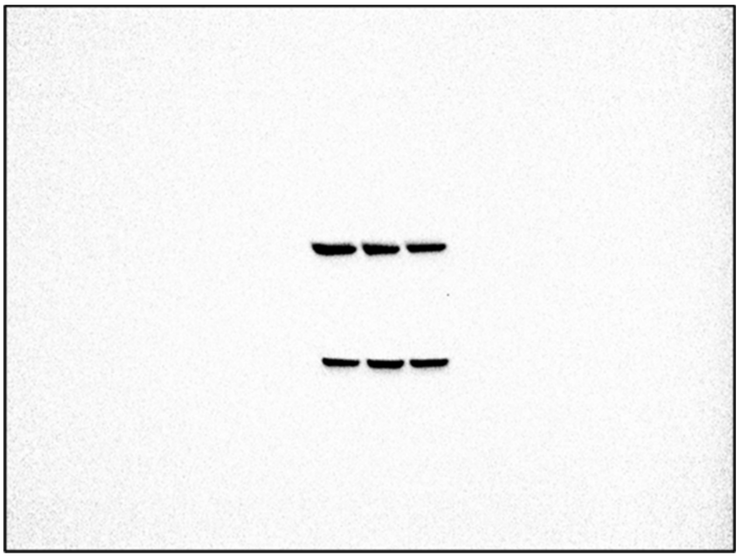

Figure 6E HLF actin

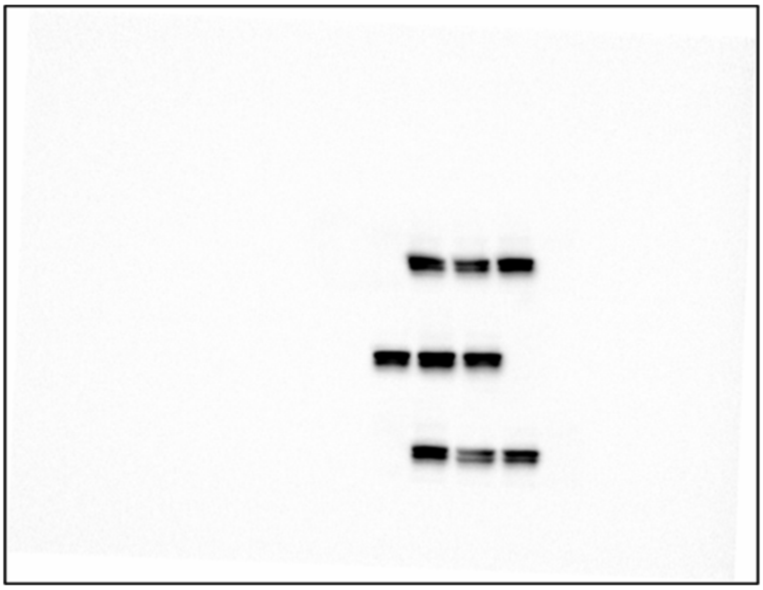

Figure 6E HLF Akt

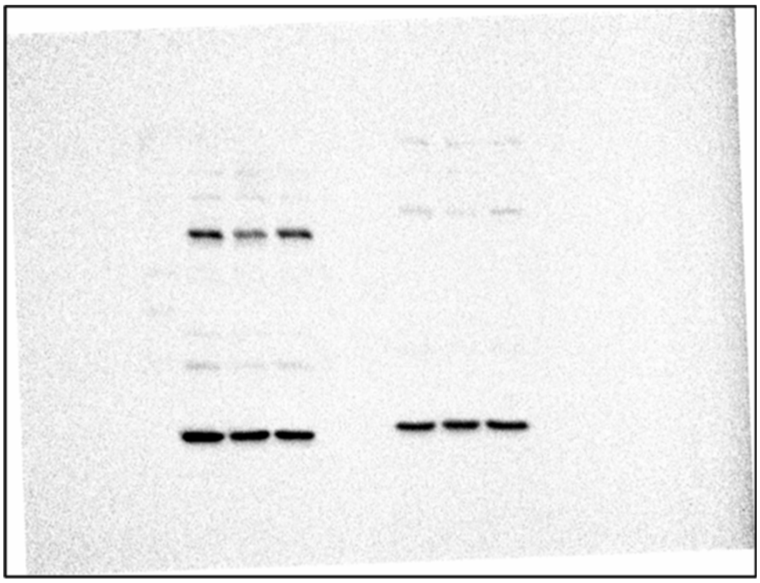

Figure 6E HLF E-cad

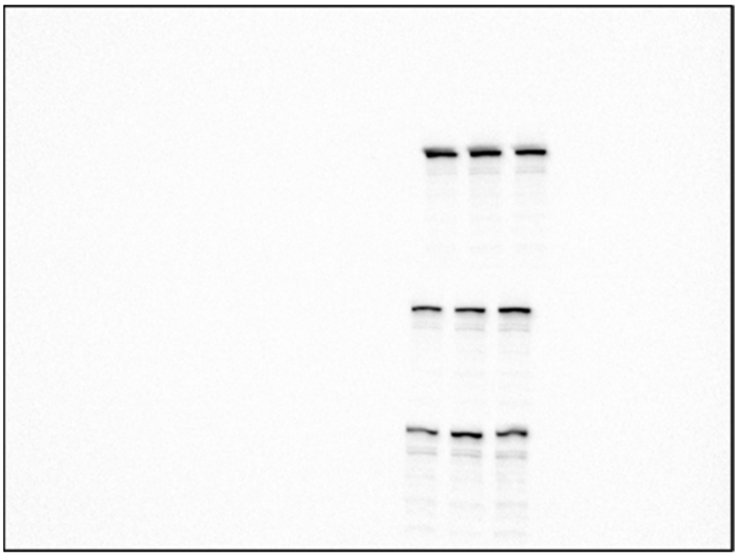

Figure 6E HLF MTOR

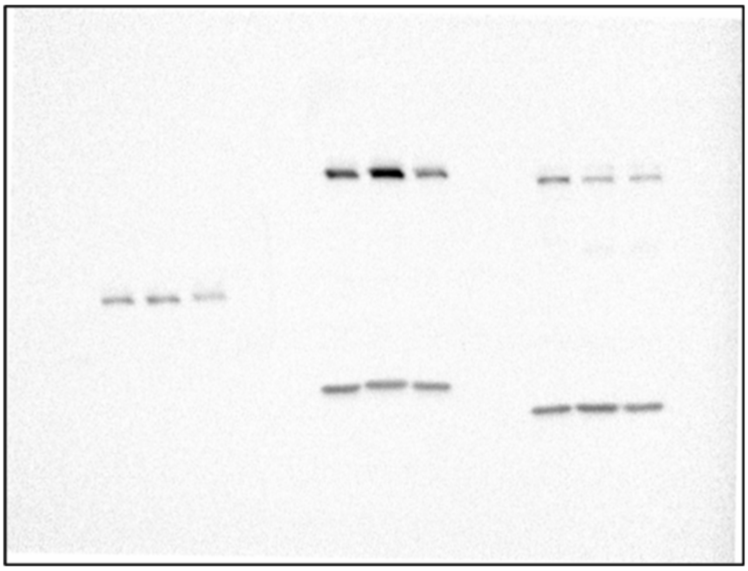

Figure 6E HLF N-cad

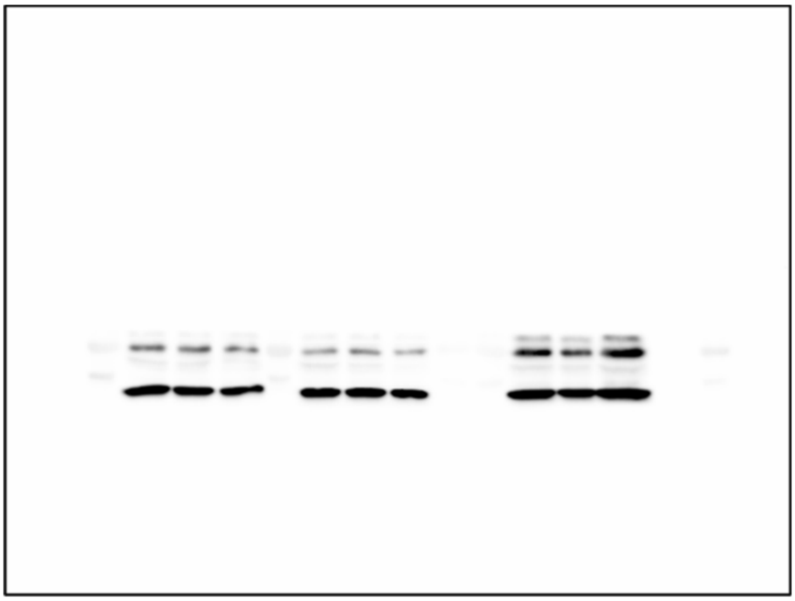

Figure 6E HLF Occludin

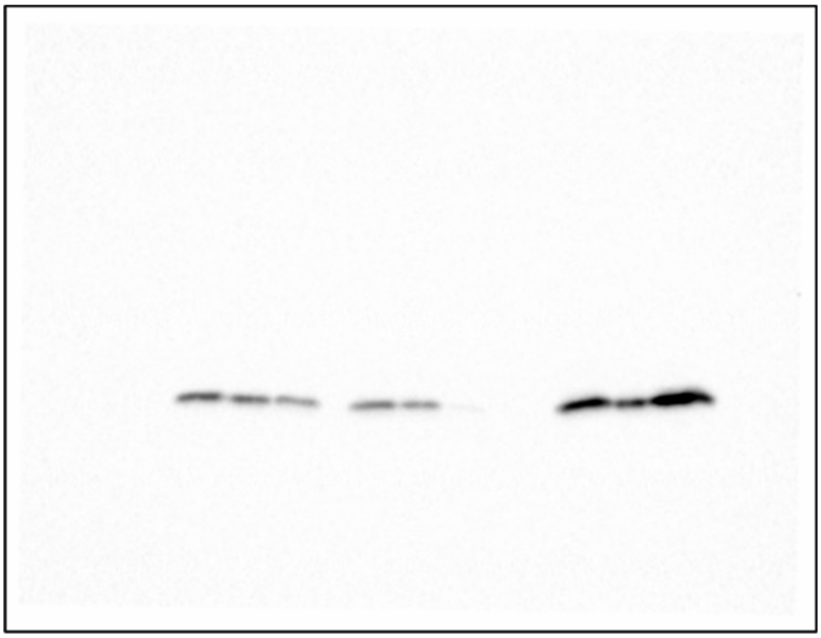

Figure 6E HLF P21

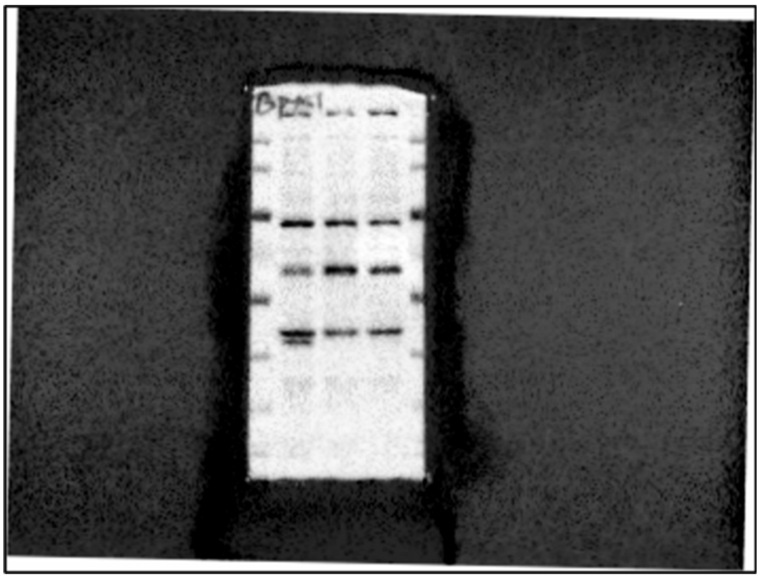

Figure 6E HLF p-Akt

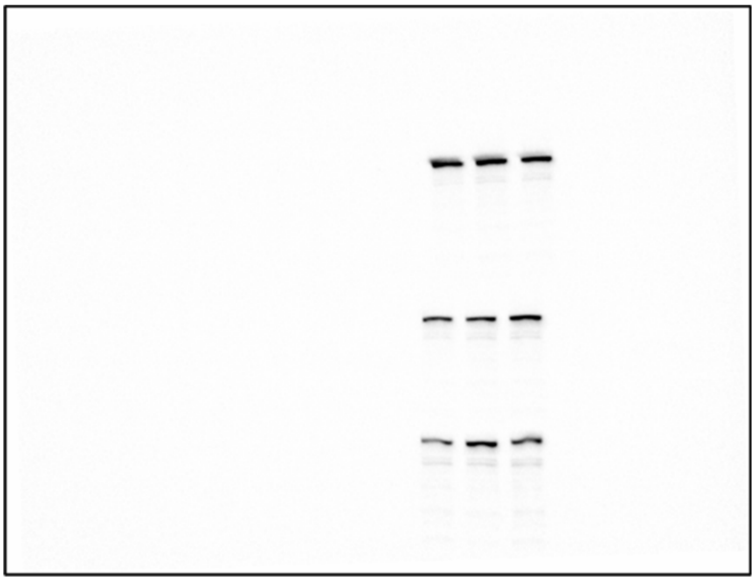

Figure 6E HLF P-MTOR

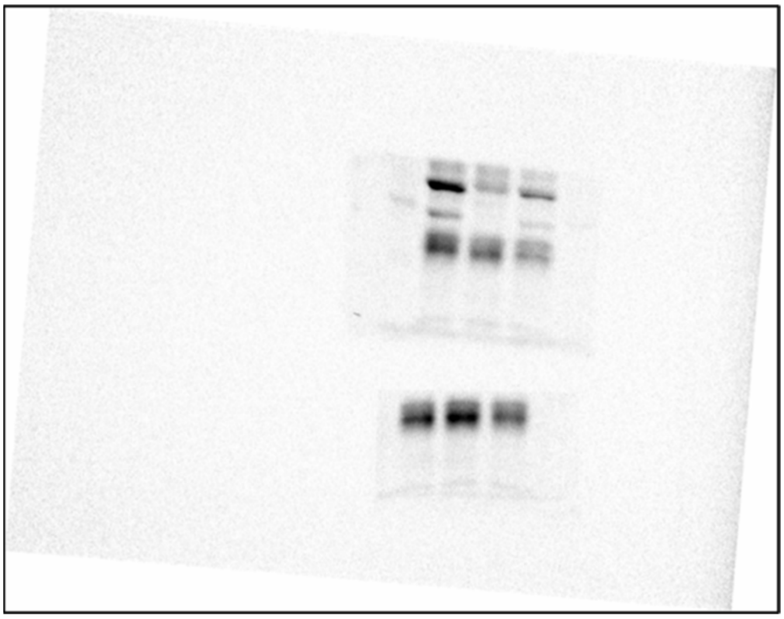

Figure 6E HLF Snail

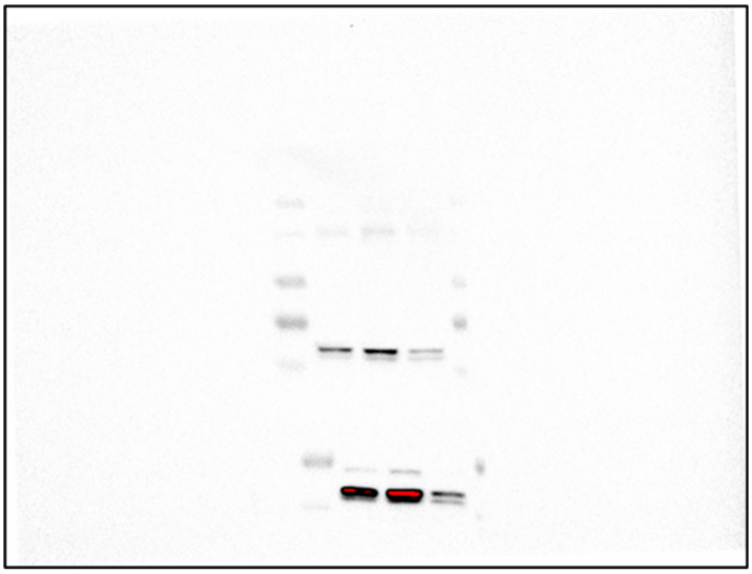

Figure 6E HLF TXNRD1

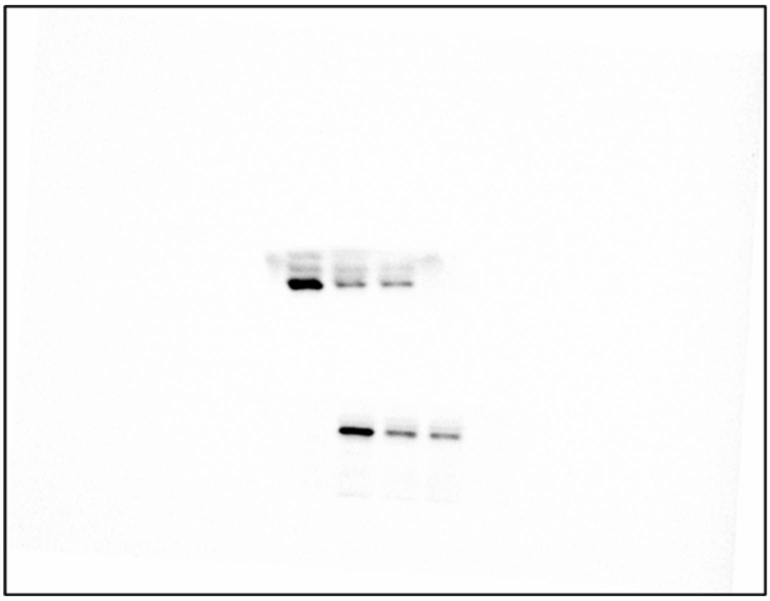

Figure 6E HLF USF2

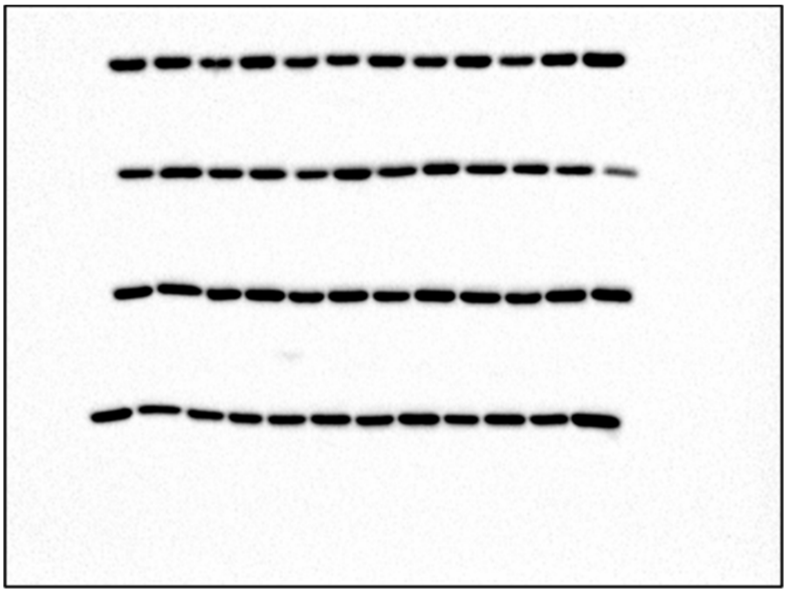

Figure 7D actin

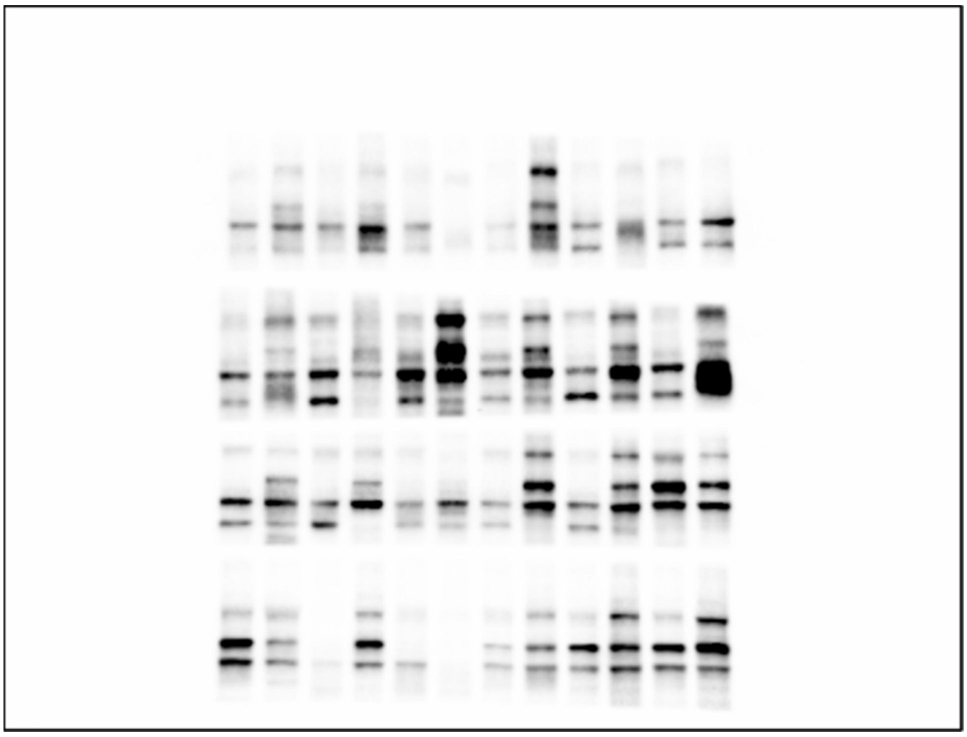

Figure 7D p-Akt

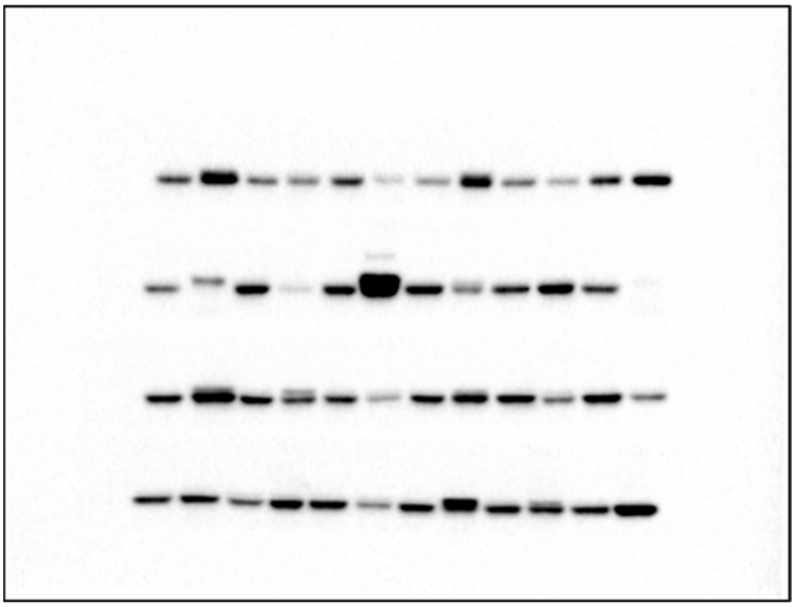

Figure 7D TXNRD1

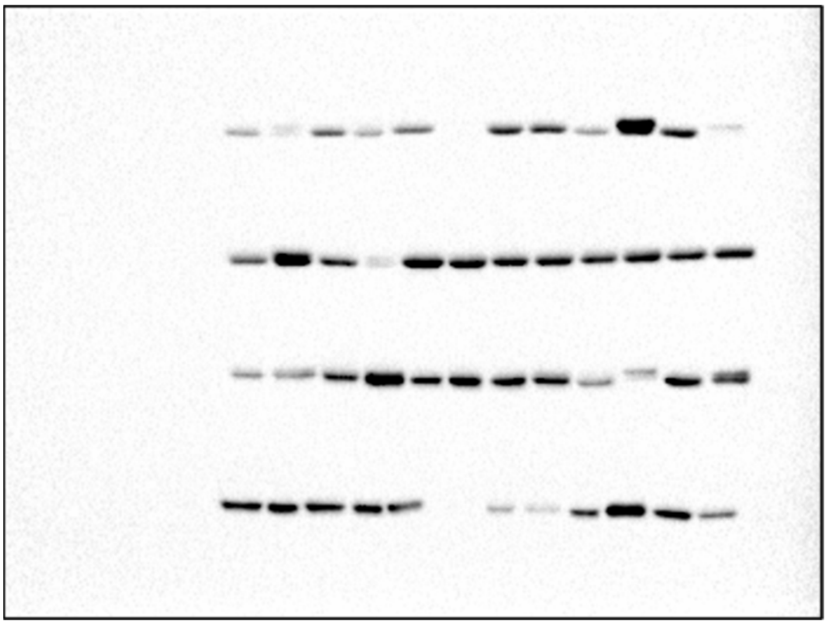

Figure 7D USF2

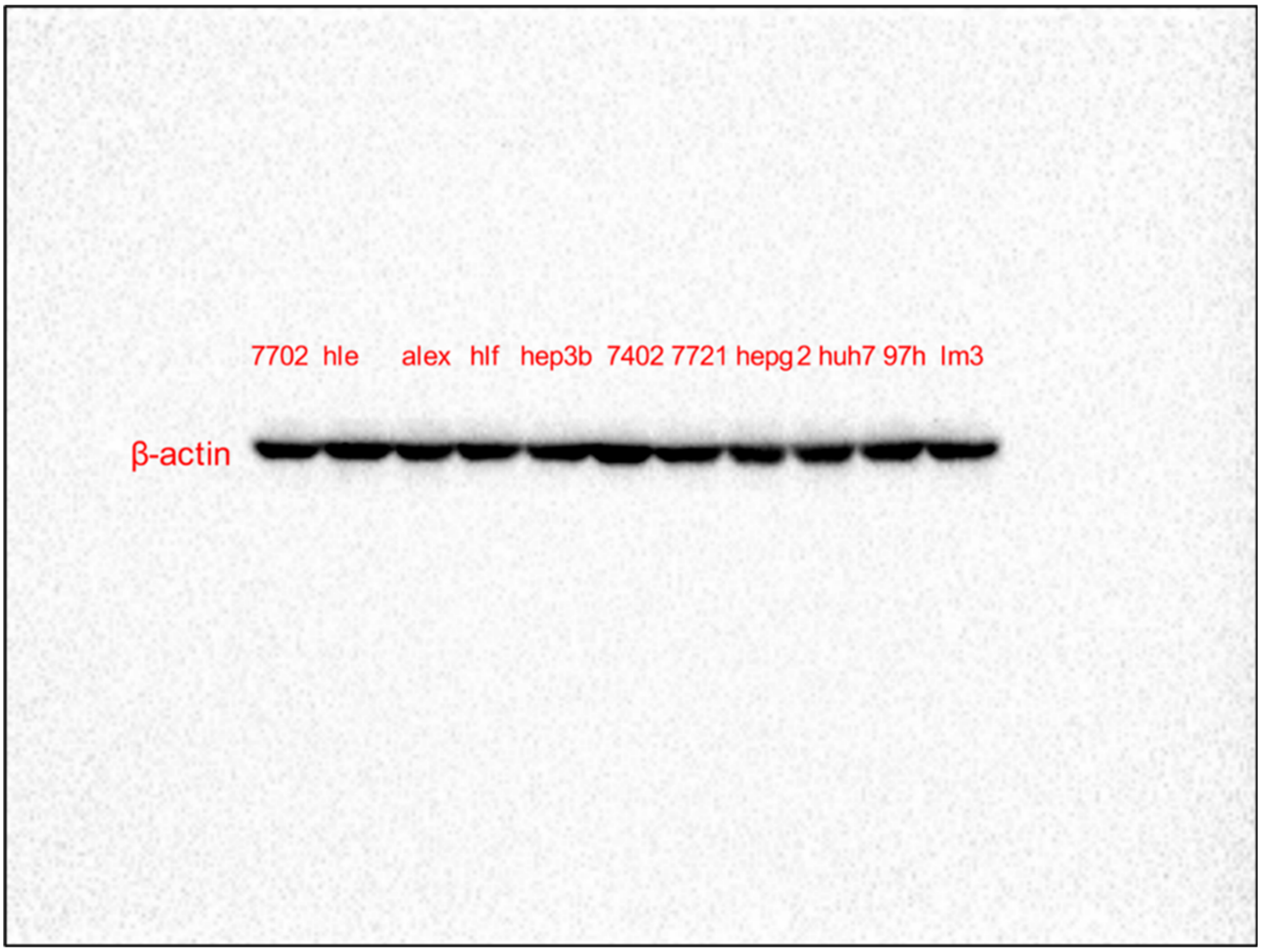

Figure S2A actin

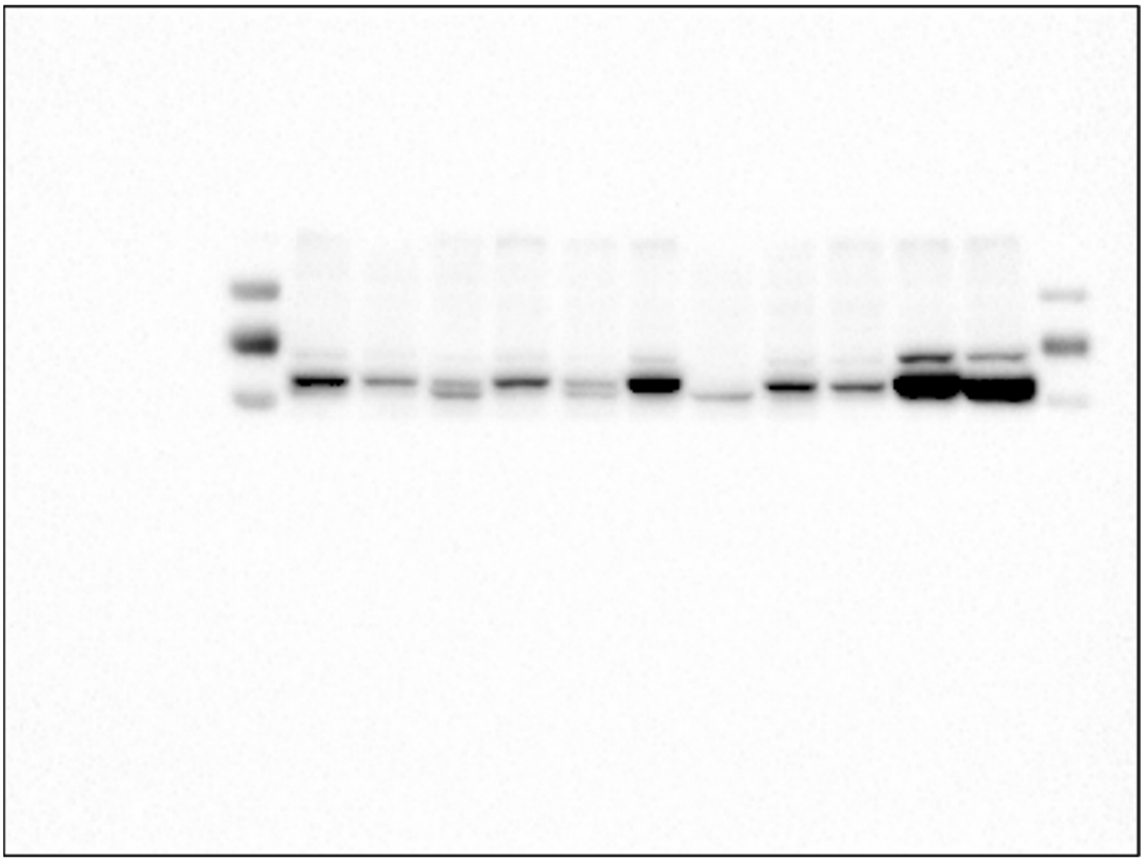

Figure S2A TXNRD1

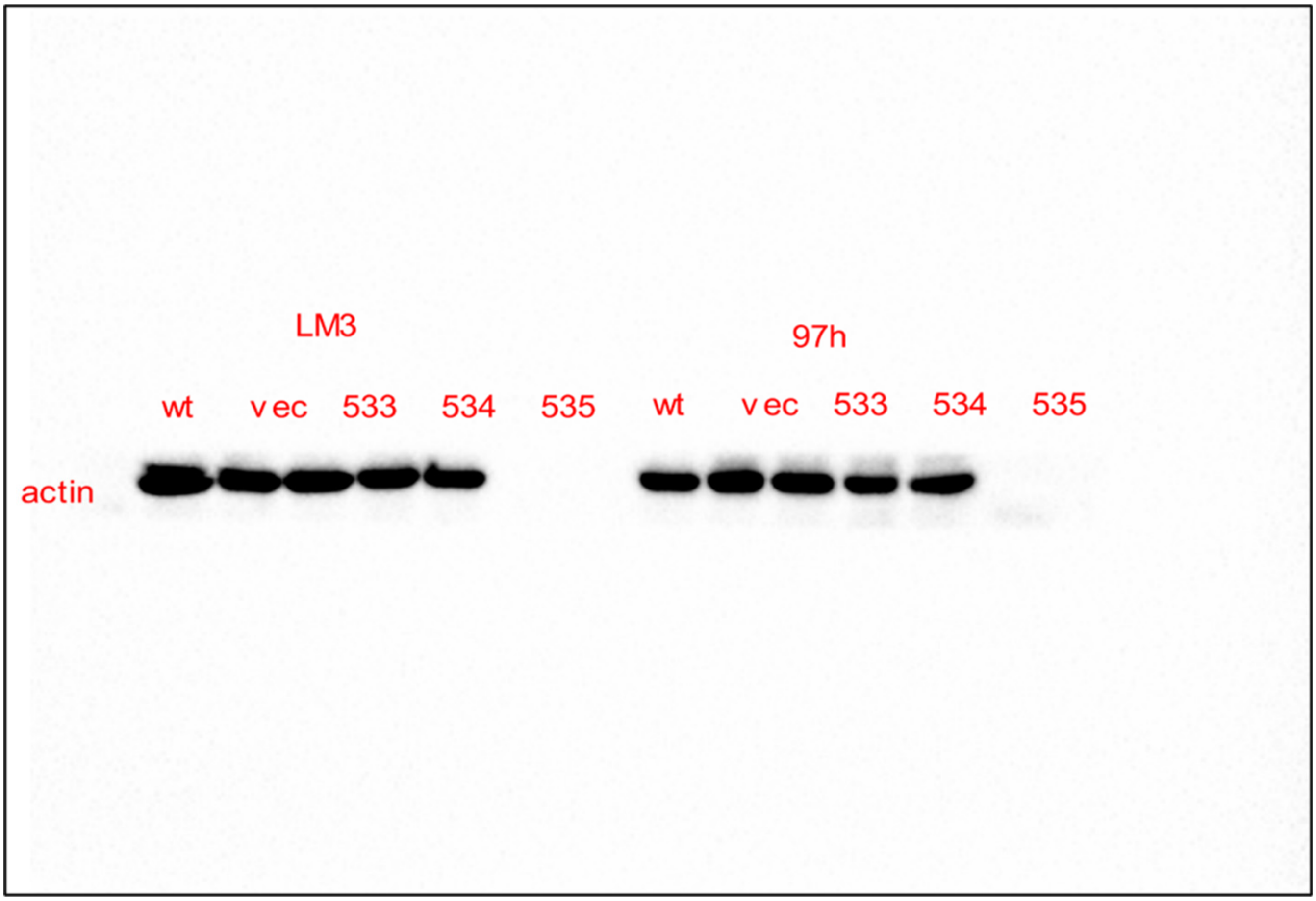

Figure S2B actin 97H

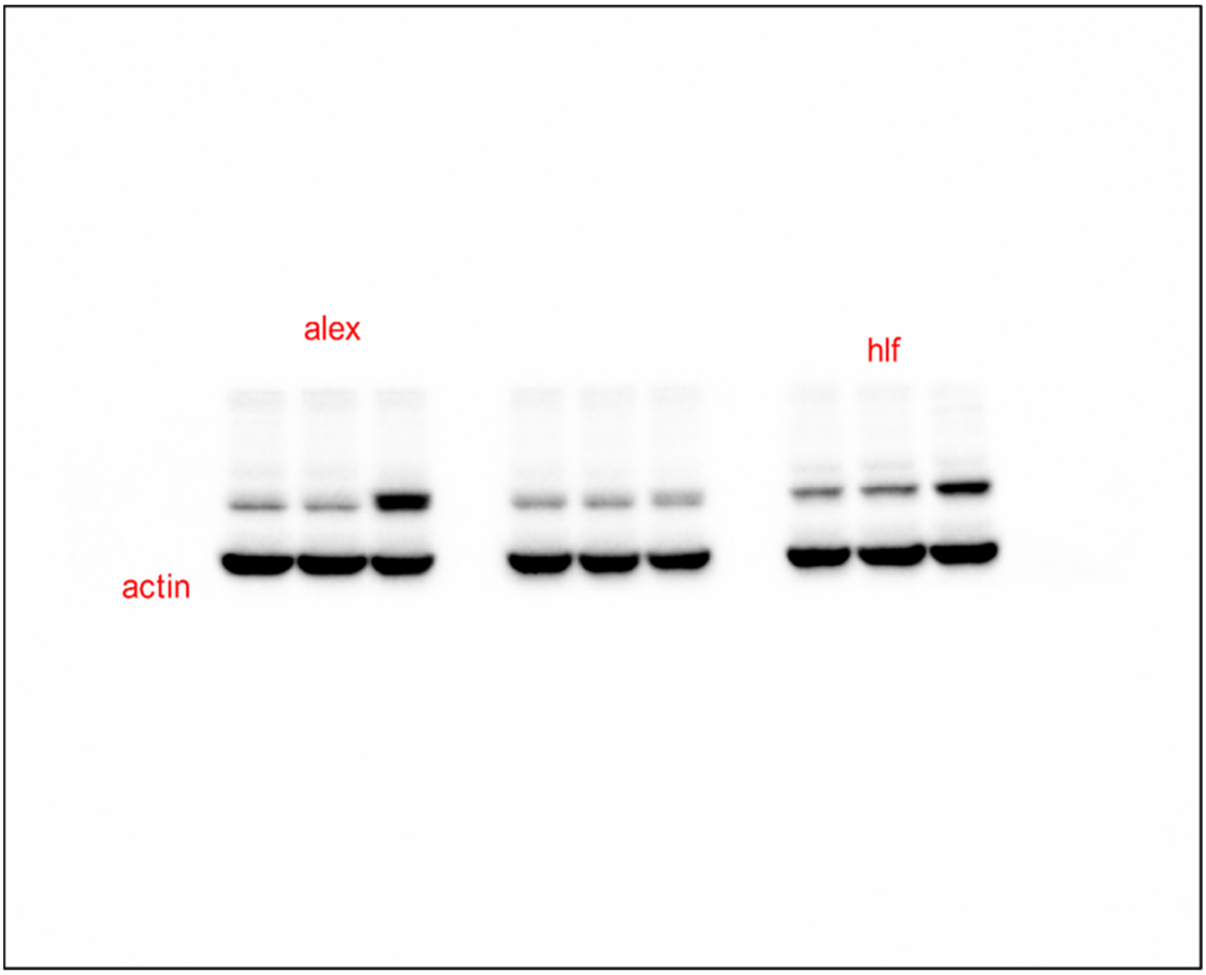

Figure S2B actin Alex

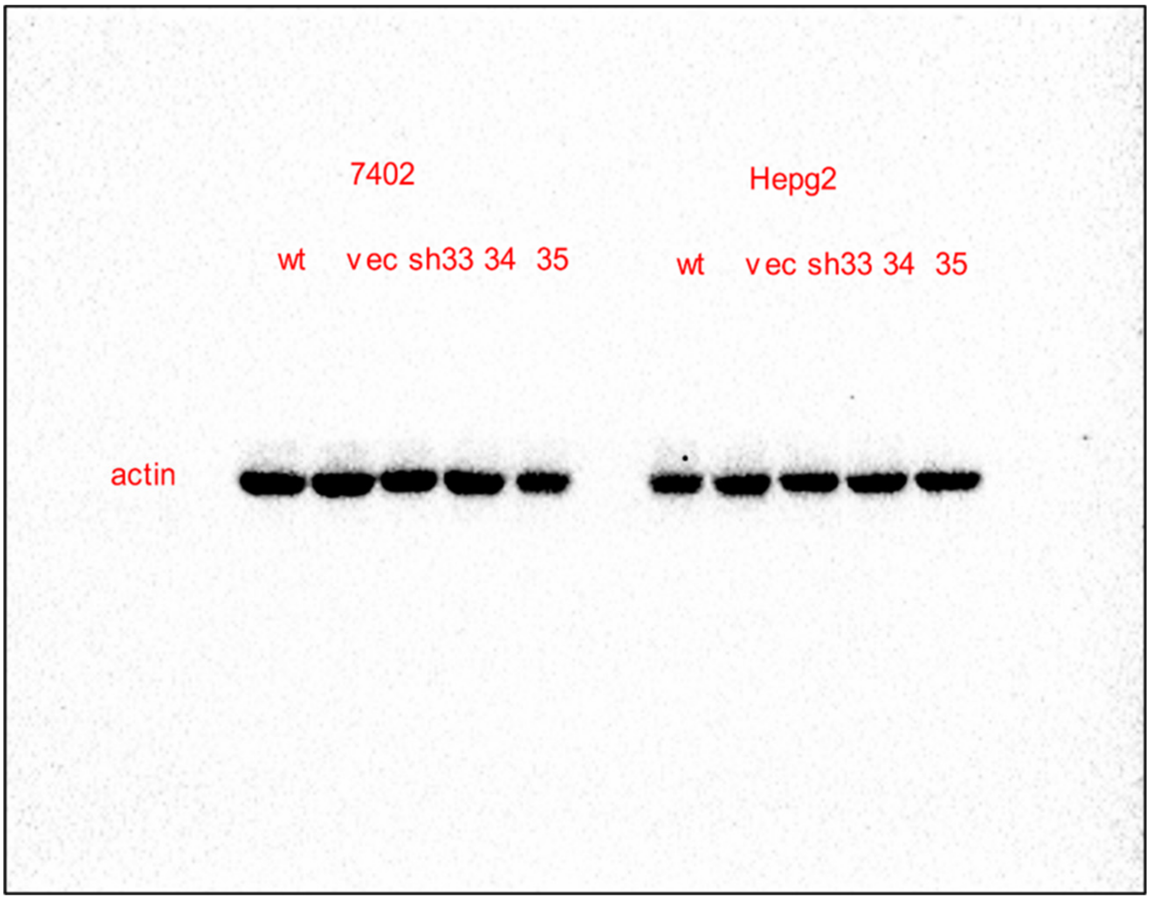

Figure S2B actin Bel-7402

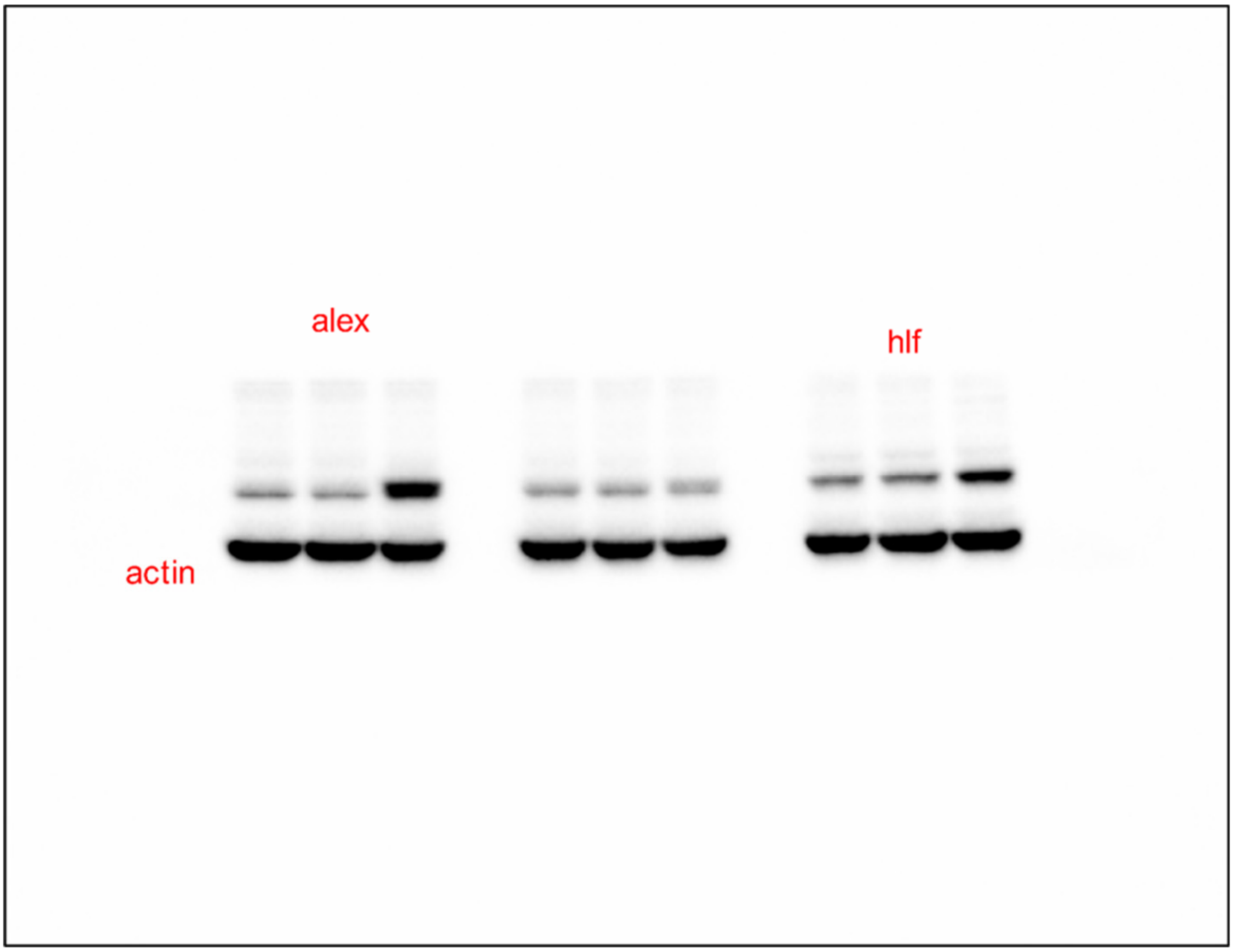

Figure S2B actin HLF

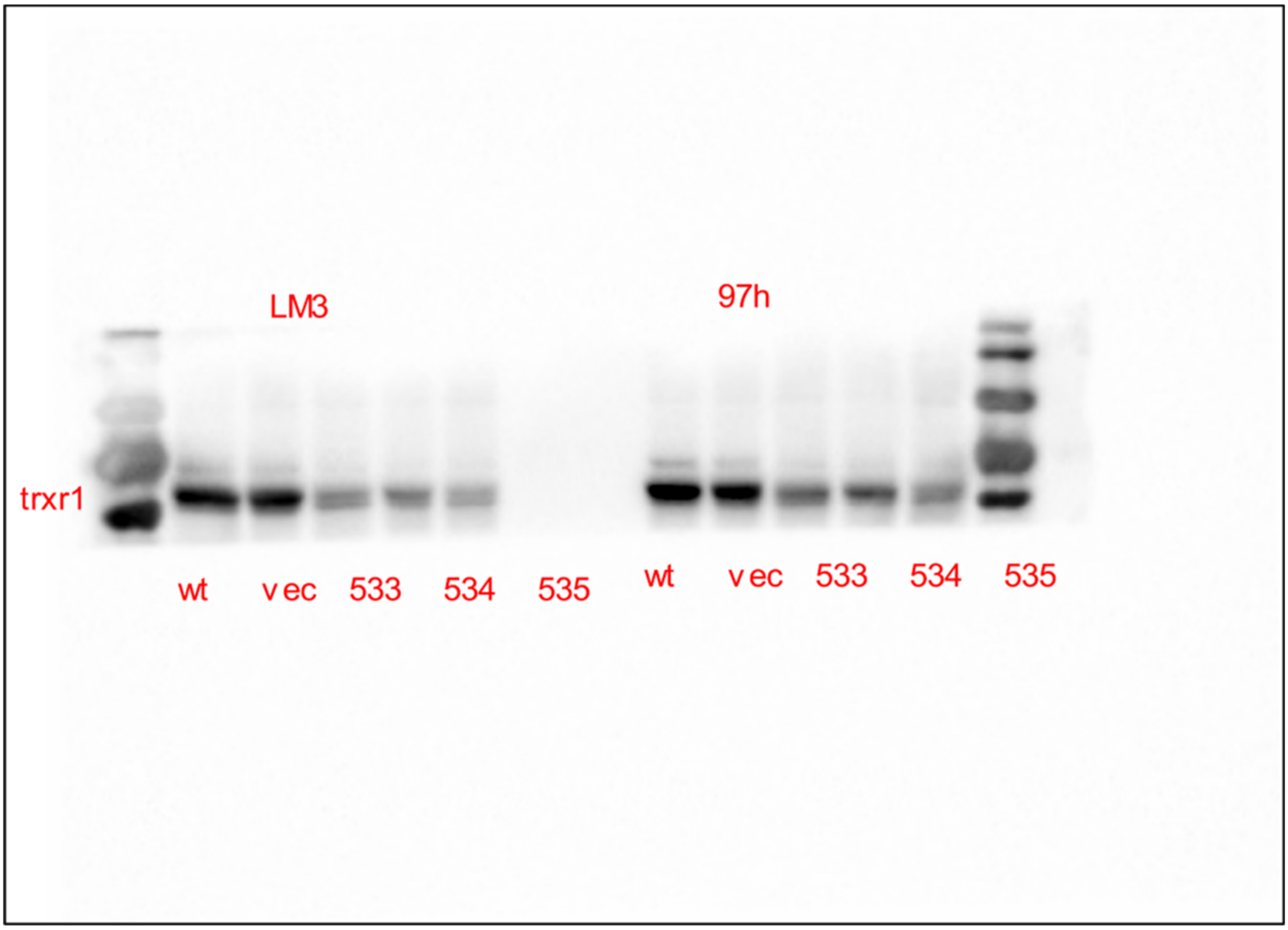

Figure S2B TXNRD1 97H

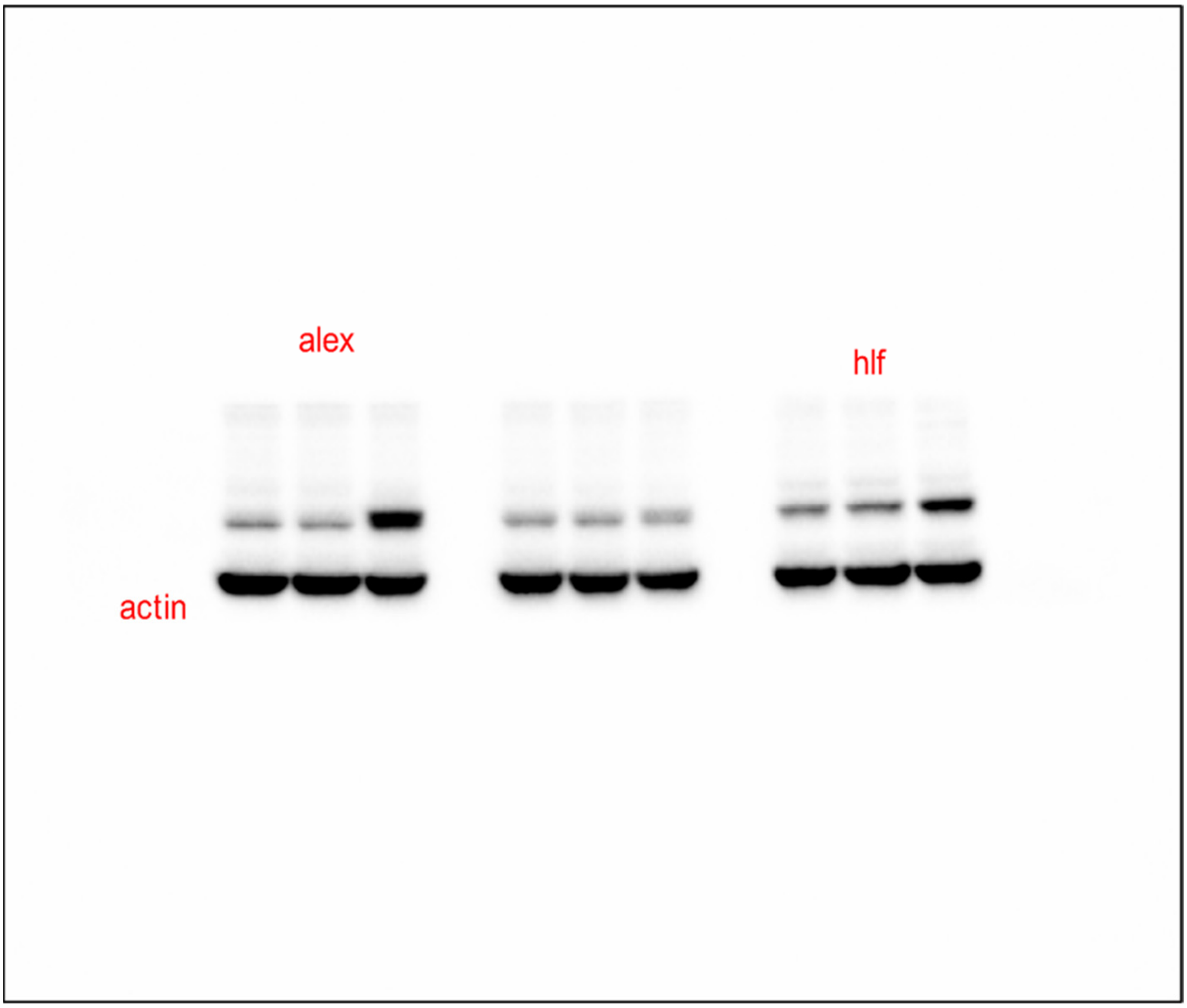

Figure S2B TXNRD1 Alex

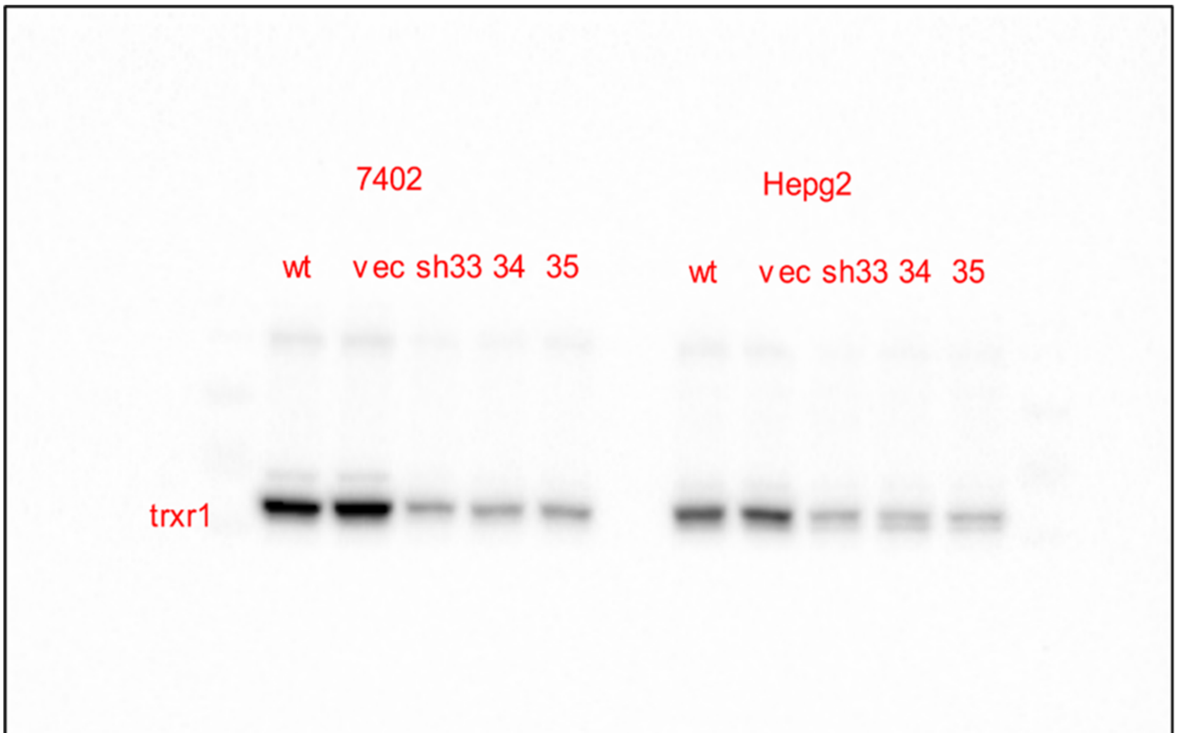

Figure S2B TXNRD1 Bel-7402

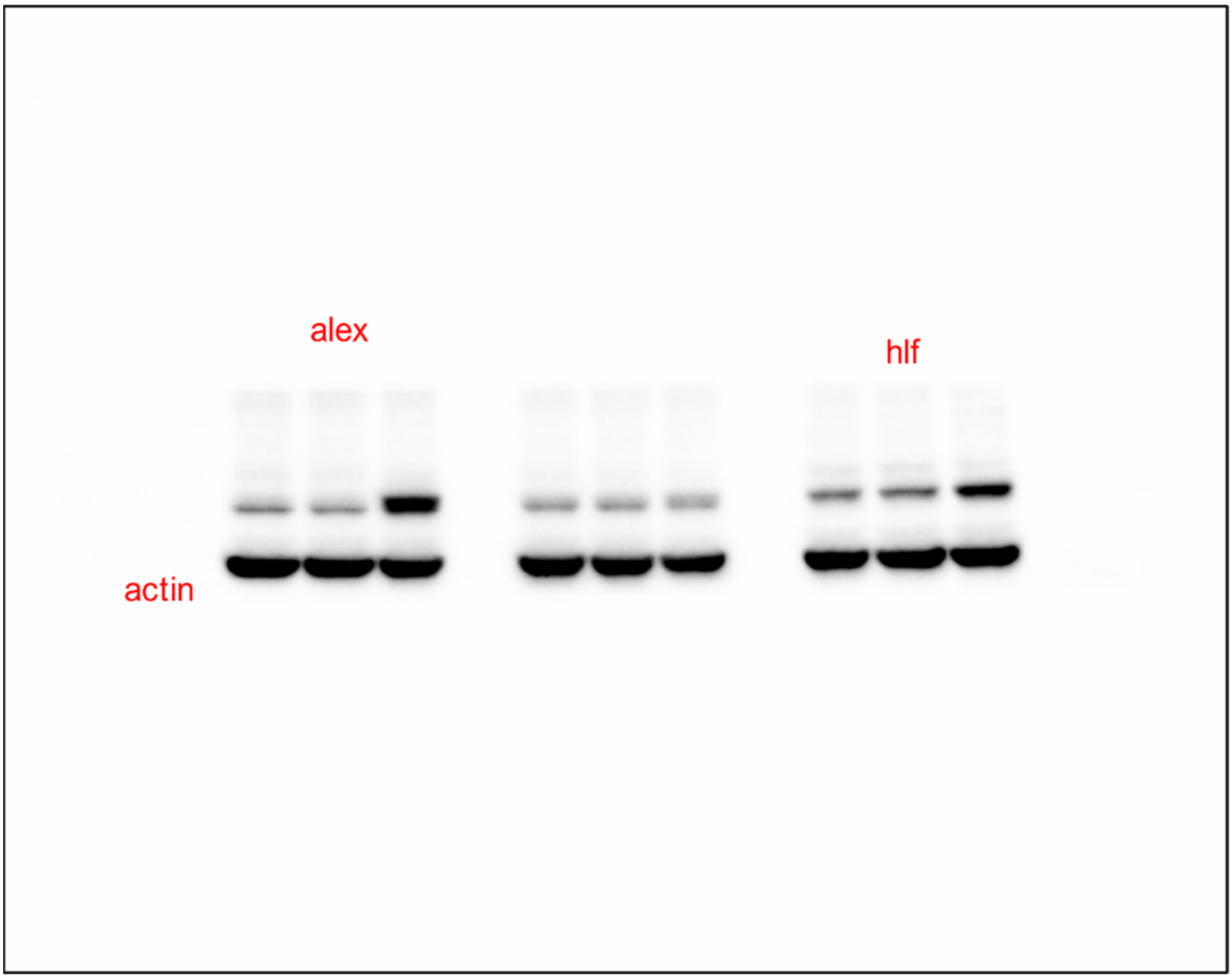

Figure S2B TXNRD1 HLF

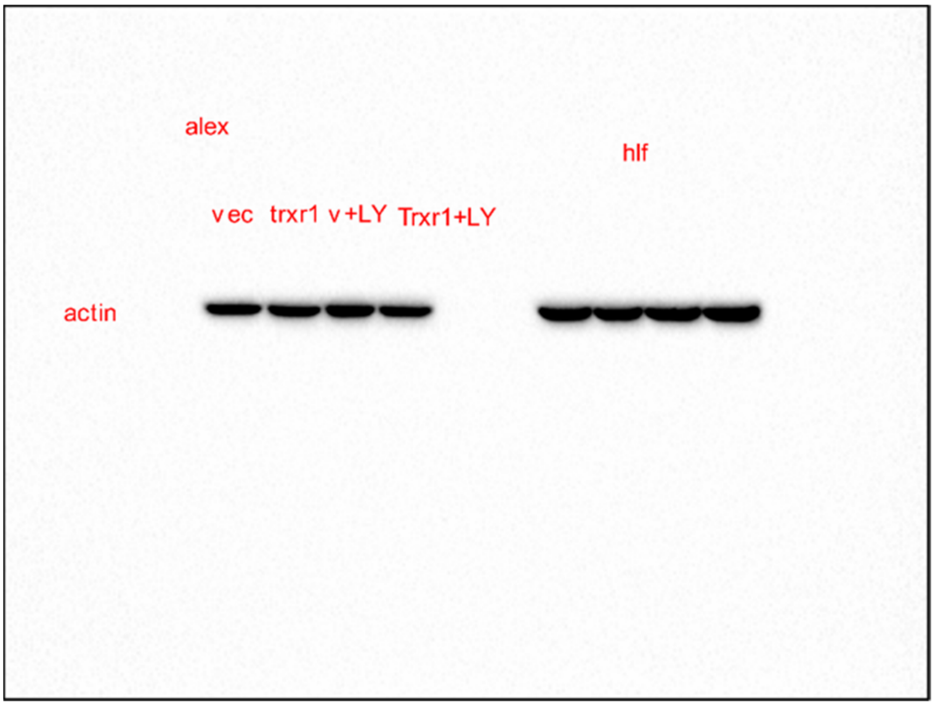

Figure S4E actin

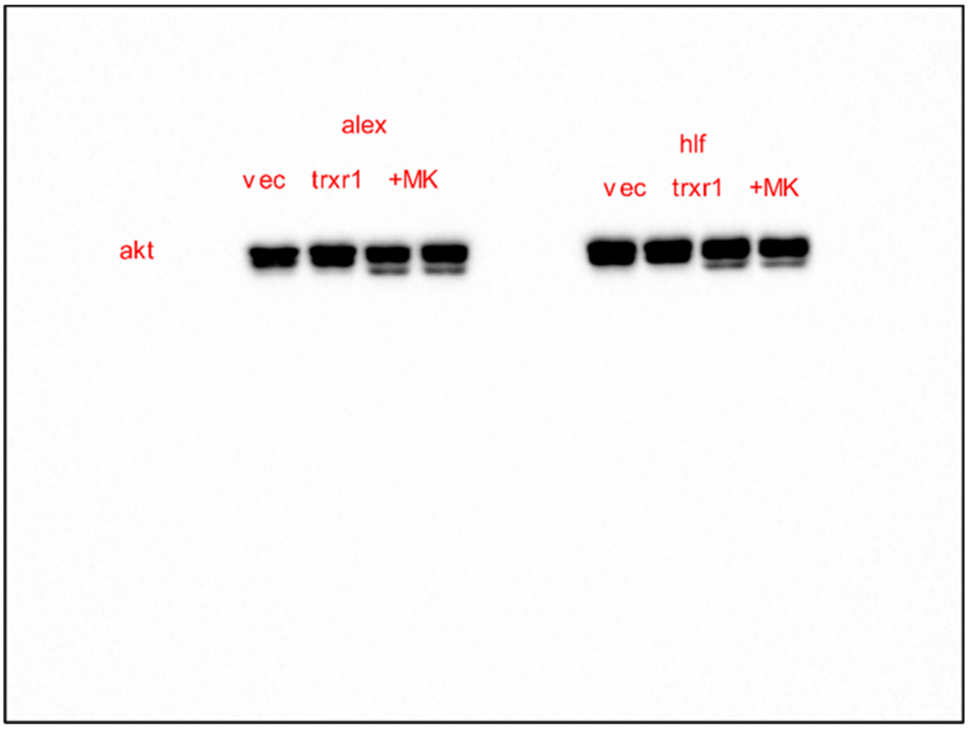

Figure S4E Akt

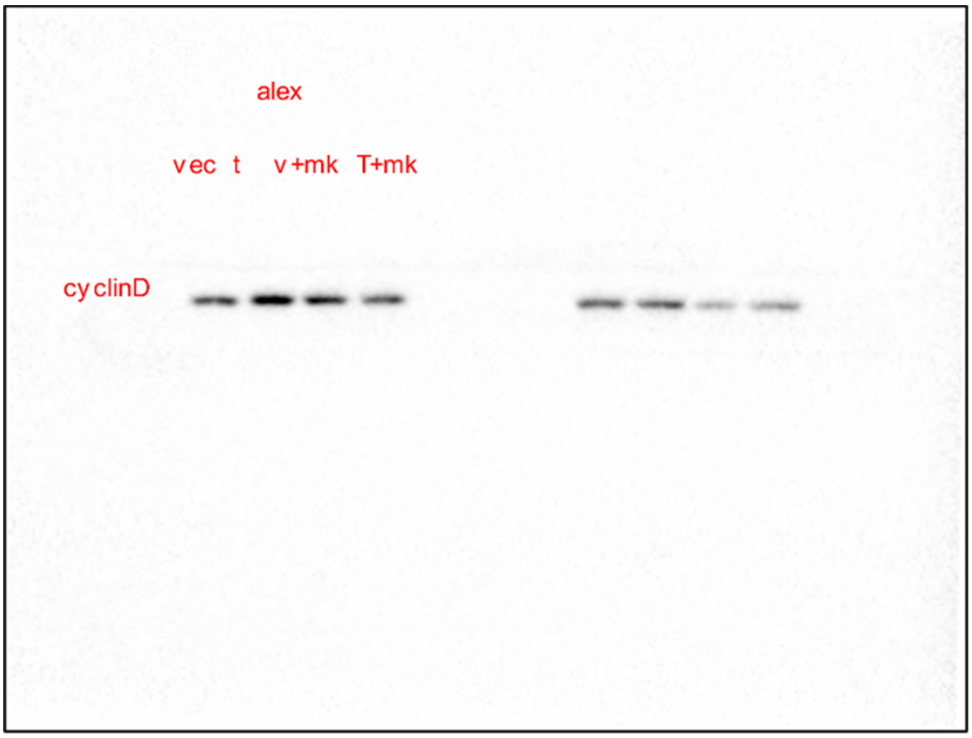

Figure S4E CyclinD

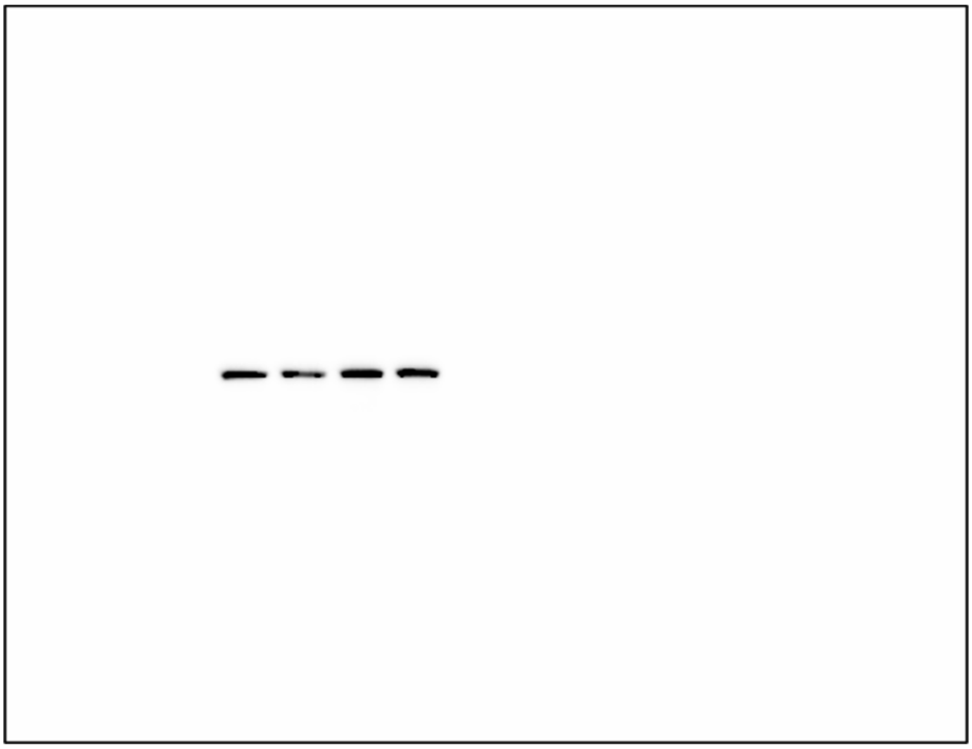

Figure S4E E-cad

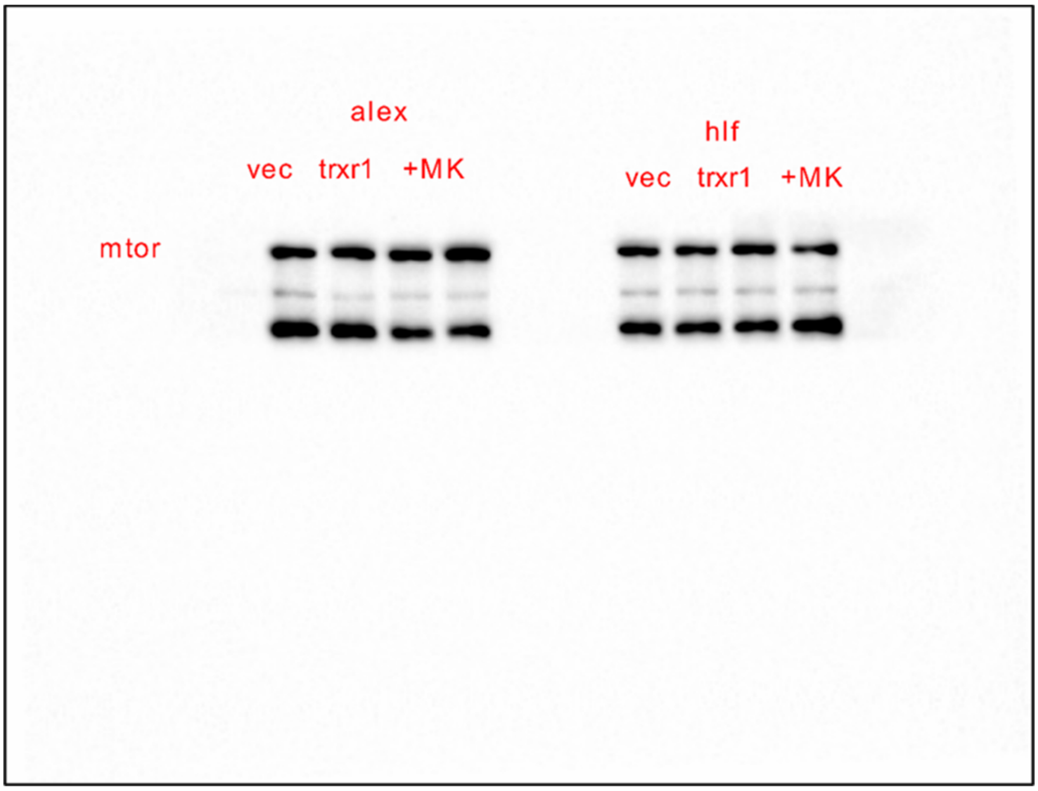

Figure S4E MTOR

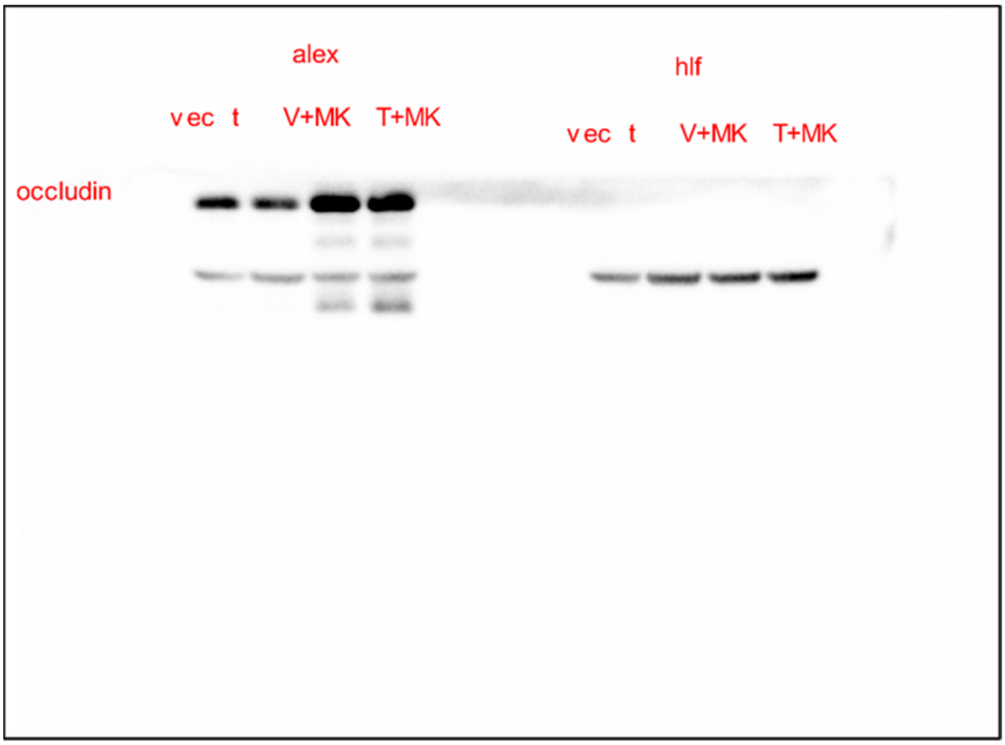

Figure S4E Occludin

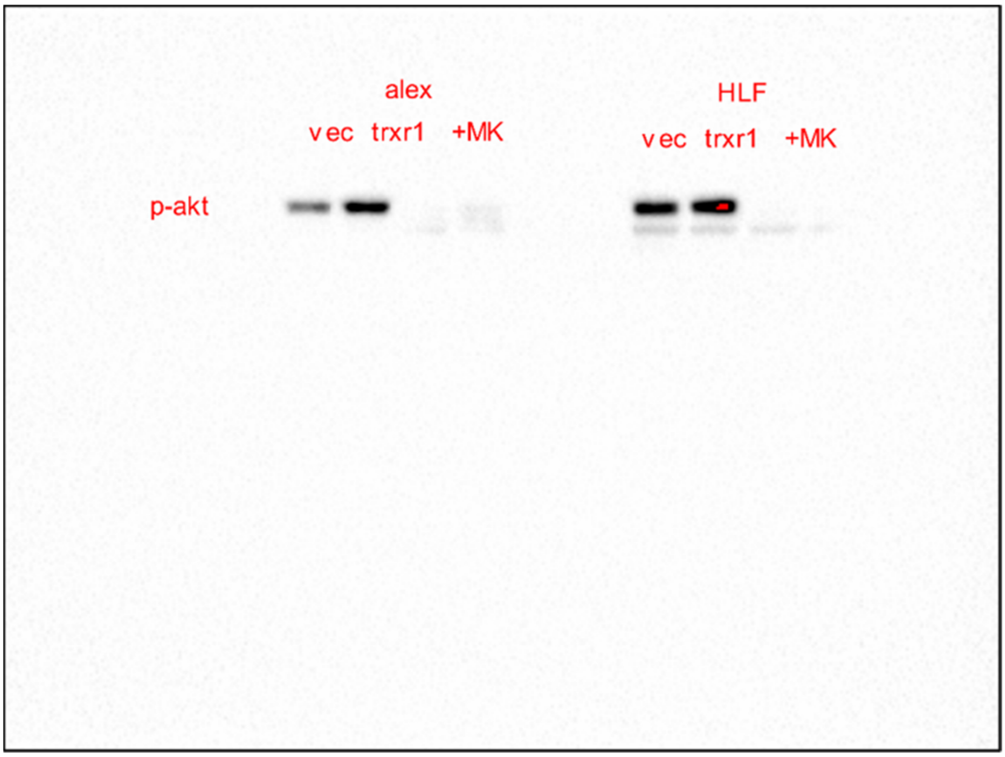

Figure S4E p-Akt

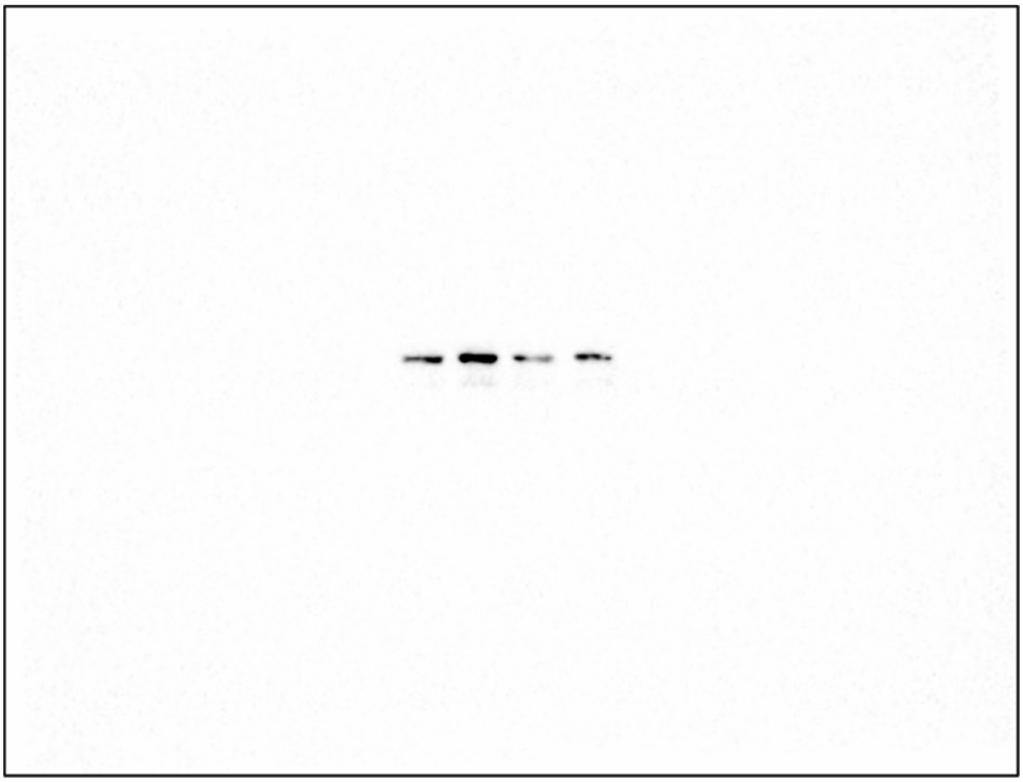

Figure S4E p-MTOR

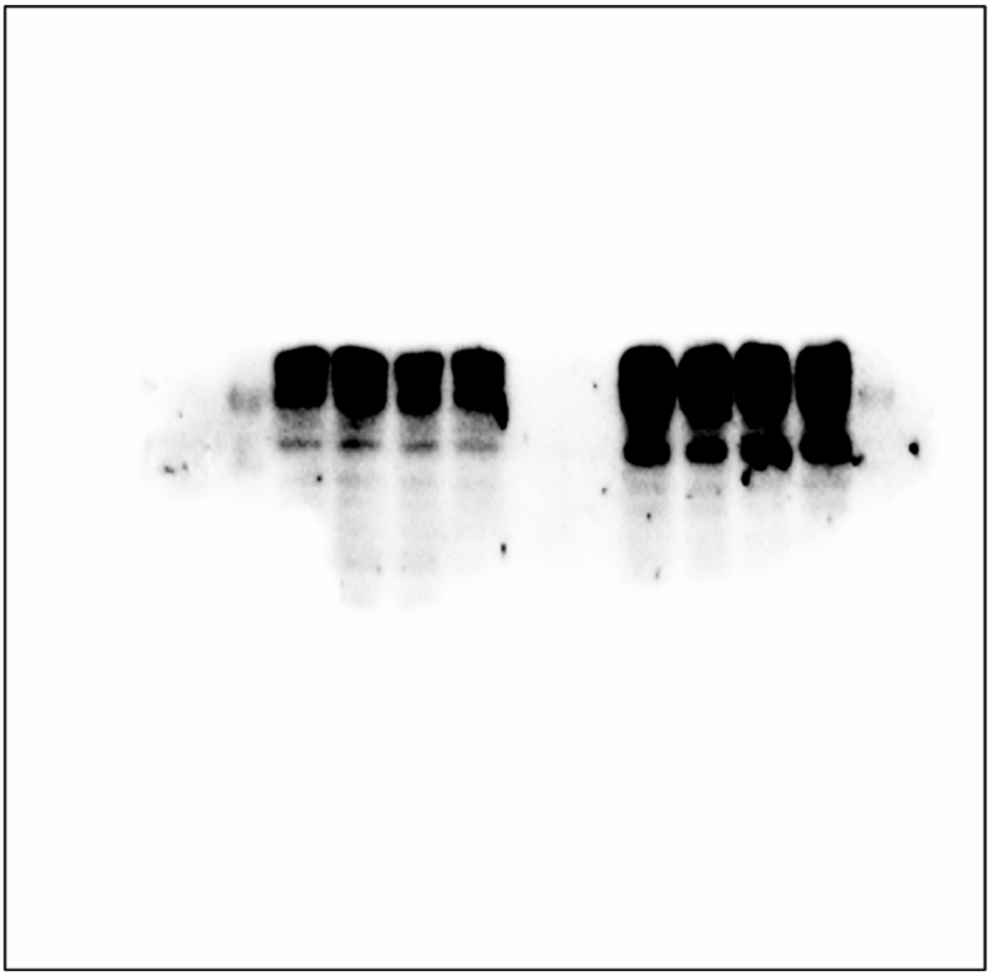

Figure S4E Snail

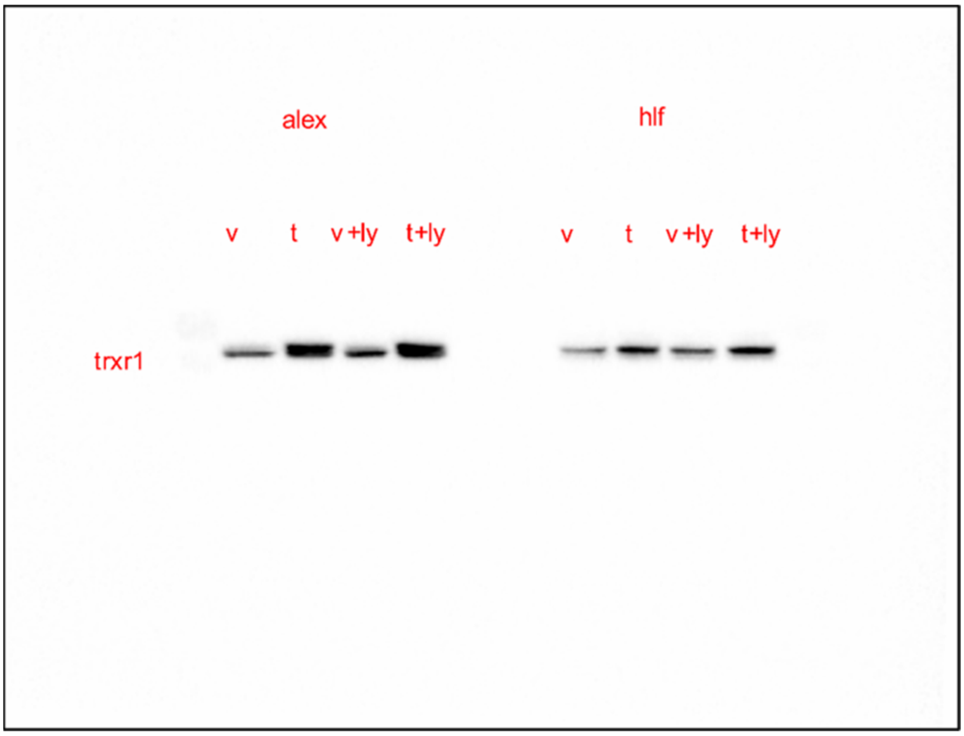

Figure S4E TXNRD1

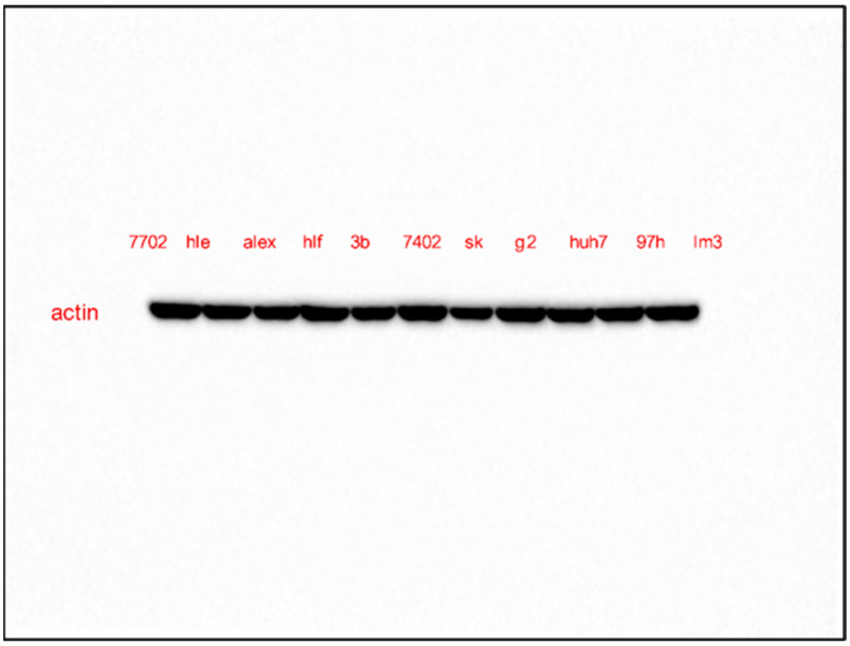

Figure S5A actin

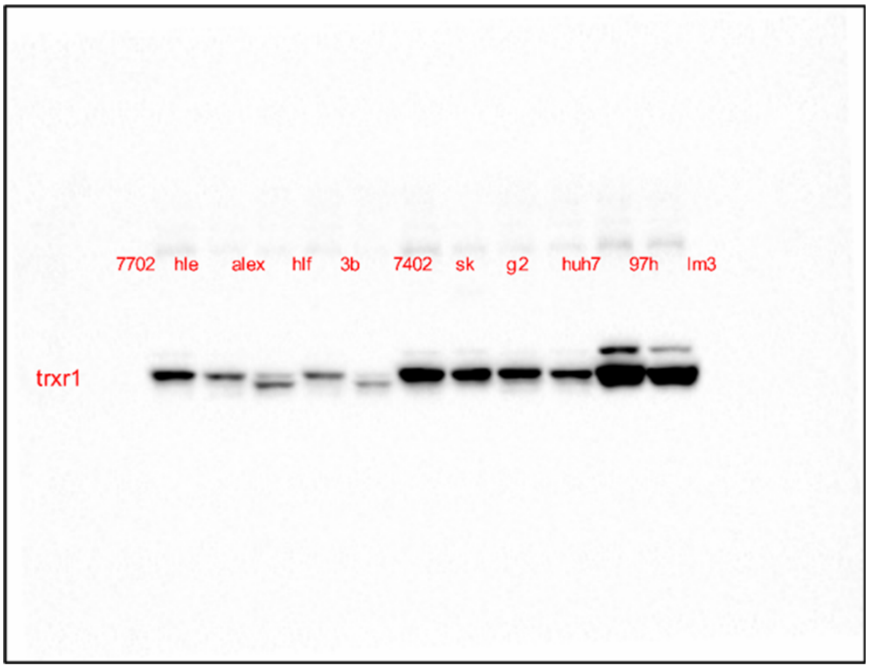

Figure S5A TXNRD1

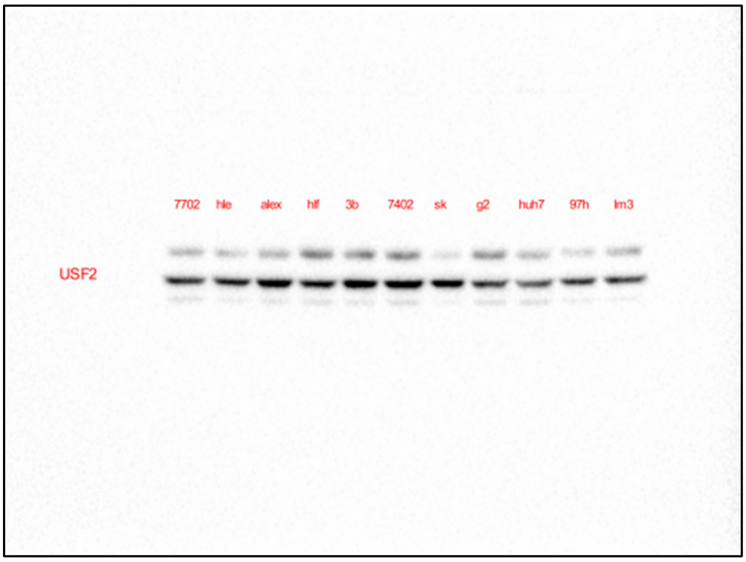

Figure S5A USF2

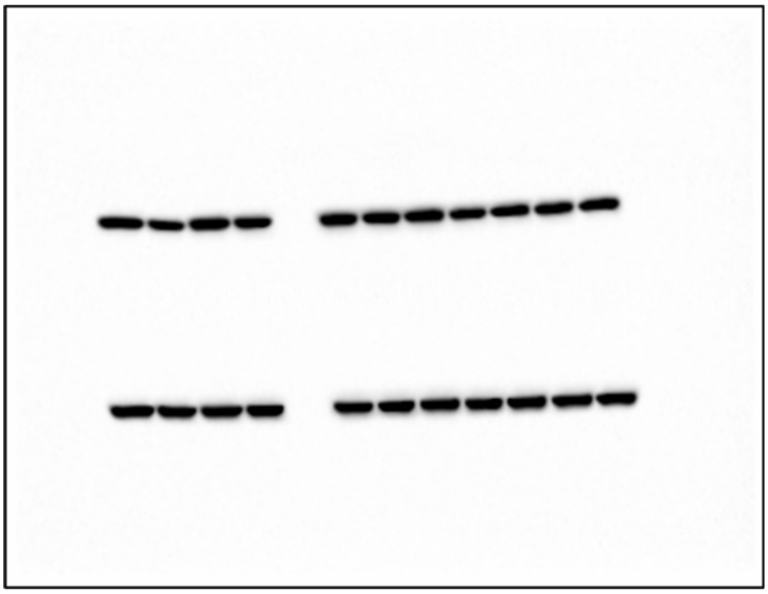

Figure S6A actin

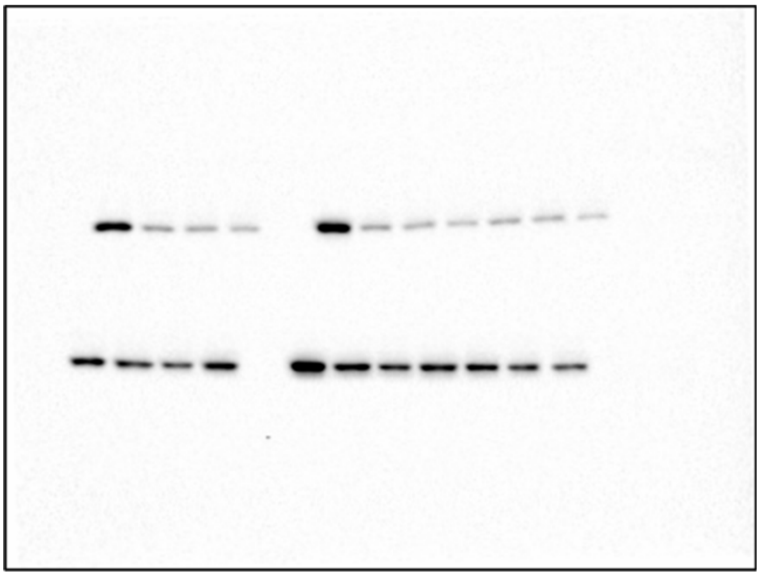

Figure S6A USF2

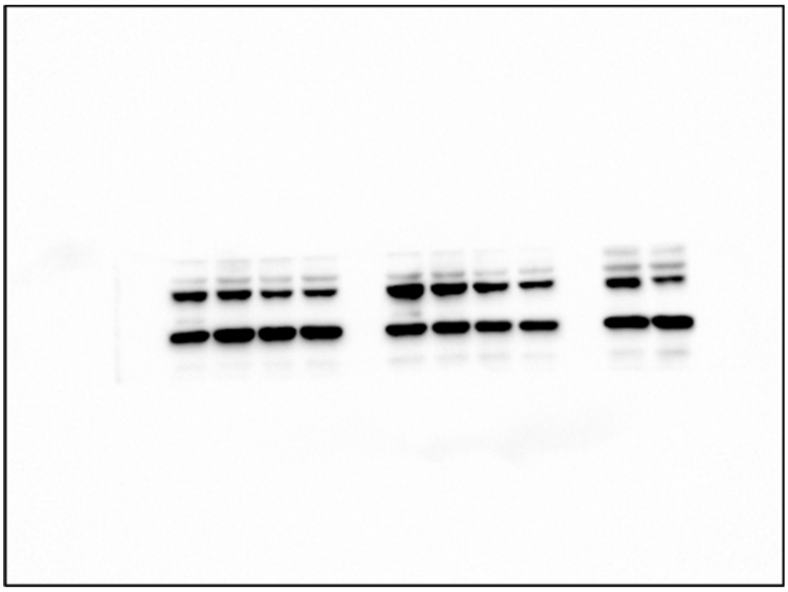

Figure S6B actin

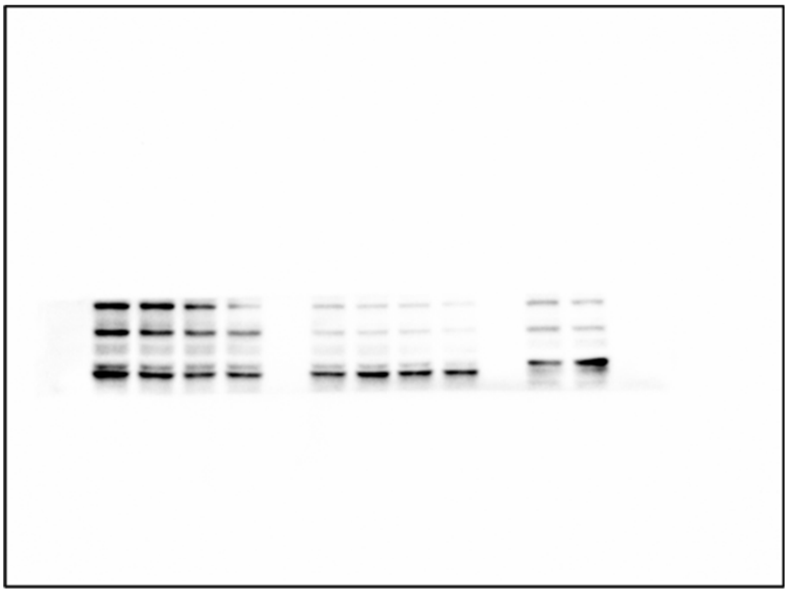

Figure S6B TXNRD1

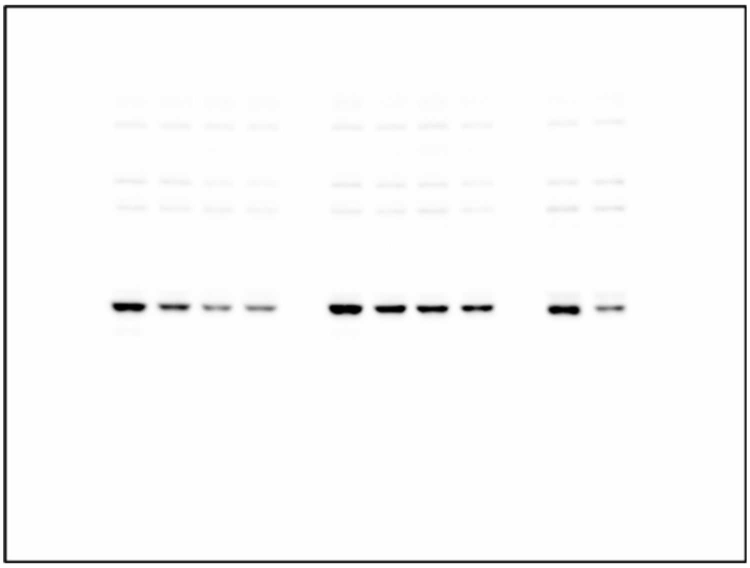

Figure S6B USF2
